# Control of cytokinin and auxin homeostasis in cyanobacteria and algae

**DOI:** 10.1093/aob/mcw194

**Published:** 2016-10-05

**Authors:** Eva Žižková, Martin Kubeš, Petre I. Dobrev, Pavel Přibyl, Jan Šimura, Lenka Zahajská, Lenka Záveská Drábková, Ondřej Novák, Václav Motyka

**Affiliations:** 1 Laboratory of Hormonal Regulations in Plants, Institute of Experimental Botany CAS, Rozvojová 263, CZ-165 02 Prague 6, Czech Republic; 2 Department of Chemical Biology and Genetics, Centre of the Region Haná for Biotechnological and Agricultural Research, Faculty of Science, Palacký University, Šlechtitelů 27, CZ-783 71 Olomouc, Czech Republic; 3 Centre for Phycology and Biorefinery Research Centre of Competence, Institute of Botany CAS, Dukelská 135, CZ-379 82 Třeboň, Czech Republic; 4 Isotope Laboratory, Institute of Experimental Botany CAS, Vídeňská 1083, CZ-142 20 Prague 4, Czech Republic; 5 Department of Taxonomy and Biosystematics, Institute of Botany CAS, Zámek 1, CZ-252 43 Průhonice, Czech Republic; 6 Laboratory of Growth Regulators, Centre of the Region Haná for Biotechnological and Agricultural Research, Faculty of Science of Palacký University and Institute of Experimental Botany CAS, Šlechtitelů 27, CZ-783 71 Olomouc, Czech Republic

**Keywords:** Cytokinin, auxin, cyanobacteria, algae, metabolism, cytokinin oxidase/dehydrogenase, cytokinin 2-methylthioderivatives, *trans*-zeatin, indole-3-acetic acid, tRNA

## Abstract

**Background and Aims** The metabolism of cytokinins (CKs) and auxins in vascular plants is relatively well understood, but data concerning their metabolic pathways in non-vascular plants are still rather rare. With the aim of filling this gap, 20 representatives of taxonomically major lineages of cyanobacteria and algae from Cyanophyceae, Xanthophyceae, Eustigmatophyceae, Porphyridiophyceae, Chlorophyceae, Ulvophyceae, Trebouxiophyceae, Zygnematophyceae and Klebsormidiophyceae were analysed for endogenous profiles of CKs and auxins and some of them were used for studies of the metabolic fate of exogenously applied radiolabelled CK, [^3^H]*trans*-zeatin (*trans*Z) and auxin ([^3^H]indole-3-acetic acid (IAA)), and the dynamics of endogenous CK and auxin pools during algal growth and cell division.

**Methods** Quantification of phytohormone levels was performed by high-performance or ultrahigh-performance liquid chromatography–electrospray tandem mass spectrometry (HPLC-MS/MS, UHPLC-MS/MS). The dynamics of exogenously applied [^3^H]*trans*Z and [^3^H]IAA in cell cultures were monitored by HPLC with on-line radioactivity detection.

**Key Results** The comprehensive screen of selected cyanobacteria and algae for endogenous CKs revealed a predominance of bioactive and phosphate CK forms while *O*- and *N*-glucosides evidently did not contribute greatly to the total CK pool. The abundance of *cis*-zeatin-type CKs and occurrence of CK 2-methylthio derivatives pointed to the tRNA pathway as a substantial source of CKs. The importance of the tRNA biosynthetic pathway was proved by the detection of tRNA-bound CKs during the course of *Scenedesmus obliquus* growth. Among auxins, free IAA and its oxidation catabolite 2-oxindole-3-acetic acid represented the prevailing endogenous forms. After treatment with [^3^H]IAA, IAA-aspartate and indole-3-acetyl-1-glucosyl ester were detected as major auxin metabolites. Moreover, different dynamics of endogenous CKs and auxin profiles during *S. obliquus* culture clearly demonstrated diverse roles of both phytohormones in algal growth and cell division.

**Conclusions** Our data suggest the existence and functioning of a complex network of metabolic pathways and activity control of CKs and auxins in cyanobacteria and algae that apparently differ from those in vascular plants.

## INTRODUCTION

Many aspects of plant growth and development are coordinated by plant hormones. Among them, cytokinins (CKs) represent one of the most important groups, playing a key role in cytokinesis and regulation of the cell cycle. In addition, CKs affect a number of other physiological processes, such as morphogenesis, apical dominance, leaf senescence, chloroplast development and seed dormancy (Miller *et al.*, 1956; Hwang *et al.*, 2012). Naturally occurring CKs are *N*^6^-substituted adenine derivatives with isoprenoid or aromatic side-chain functioning specifically at minute concentrations (10^−6^ to 10^−^^9^ m) in plant tissues (Santner *et al.*, 2009; Kieber and Schaller, 2014). [In this article, CKs are abbreviated as proposed and modified by Kamínek *et al.* (2000).] While *N*^6^-(Δ^2^-isopentenyl)adenine (iP), *trans*-zeatin (*trans*Z), *cis*-zeatin (*cis*Z), dihydrozeatin (DHZ) and their derivatives are typical representatives of isoprenoid CKs, *N*^6^-benzyladenine (BA) and its hydroxylated forms *ortho*-topolin and *meta*-topolin represent common aromatic CKs. According to their structure and physiological activity, CKs are categorized into (1) bioactive forms, including free bases and corresponding nucleosides and their precursors (nucleotides), and (2) non-active or weakly active forms, CK-*O*- and CK-*N*-glucosides (Sakakibara, 2006). In the plant kingdom, a wide spectrum of CK derivatives has been found to occur ubiquitously in vascular plants (Gajdošová *et al.*, 2011; Spíchal, 2012) as well as in bryophytes (Záveská Drábková *et al.*, 2015) and fungi (Morrison *et al.*, 2015). In contrast to vascular plants, none or only trace amounts of CK *N-*glucosylated forms and mostly rather low levels of CK *O*-glucosides have been reported in cyanobacteria or algae (Stirk *et al.*, 2003, 2013; Ördög *et al.*, 2004; Tarakhovskaya *et al.*, 2007; Hussain *et al.*, 2010).

Cyanobacteria as photosynthetic microorganisms exhibit beneficial effects on plant growth through their CK-like activity in processes of atmospheric nitrogen fixation, and thus are successfully utilized in agriculture (Stirk *et al.*, 1999, 2002; Abdel-Raouf *et al.*, 2012). A relatively simple CK metabolism in cyanobacteria was predicted based on a search of CK-related homologous genes involved in CK biosynthesis and degradation pathways. Frébort *et al.* (2011) and Kakimoto (2003) confirmed that isopentenytransferases (IPTs) catalysing the first step in CK biosynthesis in cyanobacteria have a high level of similarity with bacterial tRNA isopentenyltransferases (tRNA-IPTs) and adenylate isopentenyltransferases (AMP/ADP/ATP-IPTs). Recently, the function of gene encoded adenylate-IPT in the cyanobacterium *Nostoc* sp. PCC 7120 has been reported, although it clusters to plant tRNA-IPT (Frébortová *et al.*, 2015). Interestingly, a putative CK oxidase/dehydrogenase (CKX) homologous gene sequence involved in CK degradation was discovered in *Nostoc* sp. PCC 7120 (*NsCKX1*), but the predicted NsCKX1 similarity to plant CKX proteins was very low (Schmülling *et al.*, 2003). Moreover, functional analysis of the recombinant CKX protein named in the study as NoCKX1 revealed no detectable activity for CK downregulation (Frébortová *et al.*, 2015). In addition, no matching CKX sequences were detected in *Synechocystis* sp. PCC 6803 and *Prochlorococcus marinus*, in contrast to some other cyanobacterial species (Schmülling *et al.*, 2003; Frébort *et al.*, 2011). On the other hand, the regulatory effect of CKs on cyanobacteria metabolism has been reported for *Synechocystis* sp. PCC 6803 strain, where BA and *trans*Z enhanced RNA synthesis *in vitro*. Strongly activated RNA transcription in the presence of *trans*Z and CK-binding protein suggested the existence of a potential system of CK signal recognition, which might be transferred to the plant cell in cyanobacteria (Selivankina *et al.*, 2006). Although a gene sequence with high similarity to CK membrane receptor CRE1 in *Synechocystis* sp. PCC 6803 was found, there are still no details concerning gene expression and function (Anantharaman and Aravind, 2001; Selivankina *et al.*, 2006).

Algae are a highly diverse, non-monophyletic group of photosynthetic eukaryotes occurring in marine, freshwater and land habitats, where sufficient photosynthetic light is available (Lewis and McCourt, 2004). Variable profiles of both isoprenoid and aromatic CKs have been assigned to various algal taxa with some general trends, including *cis*Z-type prevalence and low or undetectable contents of DHZ forms and CK conjugates (Stirk *et al.*, 2003, 2013; Tarakhovskaya *et al.*, 2007). In addition, variation of endogenous CK levels was demonstrated during the cell division cycle of *Chlorella minutissima* in response to light/dark treatment, suggesting a potential requirement of CKs for algal growth (Stirk *et al.*, 2011, 2014). Even though genes coding for CK metabolic pathways have been identified in several algal species, most of them occur sporadically in comparison with vascular plants (Pils and Heyl, 2009; Kiseleva *et al.*, 2012; Lu *et al.*, 2014). Like cyanobacterial IPTs, algal IPTs are related to tRNA-IPTs rather than to adenylate ones, supporting the origin of CKs from tRNA (Lu and Xu, 2015). Taking these data together, the full set of proteins participating in CK metabolism has apparently evolved in particular in green plants (Pils and Heyl, 2009; Lu *et al.*, 2014), although as yet unknown mechanisms controlling CK homeostasis probably exist in evolutionarily older organisms, such as cyanobacteria and algae.

The phytohormone auxin is well known for its key role in the regulation of plant growth and development, especially for its impact on cell polarity and cell patterning during embryogenesis and postembryonic development, plant tropic responses, phyllotaxis, floral organs, leaf and vascular tissue formation, root development and *de novo* organogenesis (Friml *et al.*, 2003; Blilou *et al.*, 2005; Woodward and Bartel, 2005; Cheng *et al.*, 2006, 2007; Benková *et al.*, 2009; Pernisová *et al.*, 2011). In recent years our knowledge about auxin biosynthetic pathways has increased dramatically. Tryptophan-dependent biosynthesis is believed to be the main route of IAA synthesis, and currently four individual pathways are proposed, each named after the intermediate immediately downstream of tryptophan: the indole-3-acetaldoxime (IAOx), indole-3-acetamide (IAM), tryptamine and indole-3-pyruvic acid (IPyA) pathways (reviewed by Ljung, 2013; Tivendale *et al.*, 2014). A tryptophan-independent indole-3-glycerol phosphate (IGP) pathway has been described in *Arabidopsis* (Normanly *et al.*, 1993; Ouyang *et al.*, 2000; Tivendale *et al.*, 2014; Wang *et al.*, 2015).

Based on physiological activity and chemical structure, naturally occurring auxins and its derivatives can be classified to: (1) biologically active forms such as indole-3-acetic acid (IAA), 4-chloroindole-3-acetic acid (4-Cl-IAA) and indole-3-butyric acid (IBA); (2) proposed precursors of IAA biosynthetic pathways such as IAOx, IAM, tryptamine, IPyA, indole-3-acetonitrile (IAN) and indole-3-acetaldehyde (IAAld); and (3) auxin metabolites such as methyl-IAA (MeIAA) with a proposed storage role, amino acid conjugates as proposed metabolites of a degradation pathway [indole-3-acetic acid-aspartate (IAA-Asp); indole-3-acetic acid-glutamate (IAA-Glu) or inhibitors of auxin action (indole-3-acetic acid-tryptophan, IAA-tryptophan)]. Moreover, some auxin conjugates can also be hydrolysed back to free IAA via activity of amino acid conjugate hydrolases [indole-3-acetic acid-alanine, (IAA-Ala); indole-3-acetic acid-leucine (IAA-Leu); indole-3-acetic acid-phenylalanine (IAA-Phe)], auxin catabolite 2-oxindole-3-acetic acid (OxIAA) ensuring rapid inactivation of IAA via oxidation, and conjugates with sugars such as indole-3-acetyl-1-glucosyl ester (IAA-GE) and oxindole-3-acetic acid-glucosyl ester (oxIAA-GE) (Ludwig-Müller, 2011; Korasick *et al.*, 2013).

Endogenous IAA has been detected in cyanobacteria (Sergeeva *et al.*, 2002; Hussain *et al.*, 2010; Mazhar *et al.*, 2013) as well as in brown algae (Le Bail *et al.*, 2010), red algae (Ashen *et al.*, 1999; Yokoya *et al.*, 2010) and green algae (Mazur *et al.*, 2001; Cooke *et al.*, 2002; Stirk *et al.*, 2013). Likewise, other auxin precursors and metabolites represented by tryptophan, anthranilate, IAM, indole-3-ethanol and IAOx were reported in substantial amounts in algae (Yokoya *et al.*, 2010; Stirk *et al.*, 2013, 2014), while in some cyanobacteria the species IBA was found as a predominant metabolite (Hashtroudi *et al.*, 2013). Similarly, the presence of endogenous phenylacetic acid (PAA) has been detected in red and green algae (Abe *et al.*, 1974; Rocha *et al.*, 2011) and the effect of PAA application in comparison with IAA on the levels of metabolically active compounds and growth of the green alga *Chlorella vulgaris* has been described by Piotrowska-Niczyporuk and Bajguz (2014). Moreover, Sugawara *et al.* (2015) have recently shown the basic characteristics of PAA transport and metabolism and its role in auxin signalling in vascular as well as non-vascular plants.

Positive effects of IAA exogenous application have been reported, e.g. for improvement of algal growth rate (Park *et al.*, 2013), oil content increase (Maor, 2010; Jusoh *et al.*, 2015) and induction of tolerance of higher salinities and temperatures (Nowak *et al.*, 1988; Piotrowska-Niczyporuk and Bajguz, 2014). Additionally, the enhancement of growth parameters and biomass production after inoculation with some cyanobacterial strains due to their auxin-like activity were observed in wheat (Mazhar *et al.*, 2013) and sunflower (Varalakshmi and Malliga, 2012), for instance.

The goal of this study was to characterize CK and auxin metabolism in cyanobacteria and algae as the ancestors of vascular plants. In order to get insight into potential metabolic pathways involved in the control of the homeostasis of the two phytohormones, we quantified the endogenous profiles of CKs and auxins and determined their levels following exogenous application of radiolabelled *trans*Z and IAA in distinct taxa of cyanobacteria and algae. Last but not least, endogenous CK and auxin pools were determined during the culture of *Scenedesmus obliquus* with the aim of demonstrating the roles of both phytohormones in algal growth and cell division.

## MATERIALS AND METHODS

### Chemicals

All CKs were supplied by Olchemim, Ltd (Olomouc, Czech Republic); other chemicals were purchased from Sigma-Aldrich. (St Louis, MO, USA). [2-^3^H]*trans*-zeatin ([^3^H]*trans*Z; specific radioactivity 29·7 Ci mmol^−1^), [2-^3^H]*cis*-zeatin ([^3^H]*cis*Z; specific radioactivity 29·7 Ci mmol^−1^) and [2-^3^H]*N*^6^-(Δ^2^-isopentenyl)adenine ([^3^H]iP; specific radioactivity 35·1 Ci mmol^−1^) were supplied by the Isotope Laboratory, Institute of Experimental Botany CAS (Prague, Czech Republic). [5-^3^H]indole-3-acetic acid ([^3^H]IAA), [5-^3^H]2,4-dichlorophenoxy acetic acid ([^3^H]2,4-D) and [4-^3^H]naphthalene-1-acetic acid ([^3^H]NAA) (speciﬁc radioactivity 20·0 Ci mmol^−1^ each) were supplied by American Radiolabeled Chemicals (St Louis, MO, USA).

### Experimental material

**Fig. 1.**
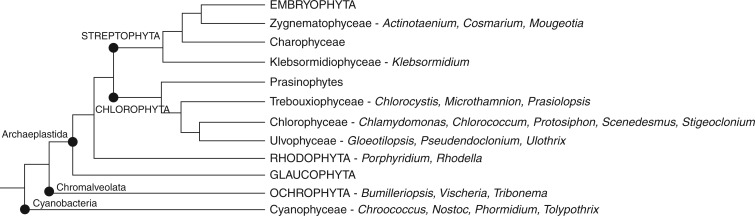
The position of selected cyanobacterial and algal taxa within a simplified phylogenetic tree. The phylogenetic tree is based on different data sources from the whole chloroplast genome and nuclear rDNA (Riisberg *et al.*, 2009; Ruhfel *et al.*, 2014). For a complete list of analysed species see Table S1.

Twenty representatives of taxonomically major lineages of cyanobacteria and algae belonging to nine classes (Cyanophyceae, Xanthophyceae, Eustigmatophyceae, Porphyridiophyceae, Chlorophyceae, Ulvophyceae, Trebouxiophyceae, Zygnematophyceae and Klebsormidiophyceae) were provided by the Culture Collection of Autotrophic Organisms (CCALA, http://ccala.butbn.cas.cz/index.php). An overview of the selected species used in the study as well as their taxonomic classification is given in Supplementary Data Table S1, and their position within a simplified phylogenetic tree is shown in Fig. 1.

### Culture conditions for cyanobacteria and algae

In CCALA, cyanobacterial and algal strains have been maintained on agar slants under controlled light and temperature conditions, i.e. light intensity 23 μmol photons m^−^^2^ s^−1^ of photosynthetically active radiation (PAR), a 12-h light/12-h dark photoperiod and temperature 12–15 °C. In order to adapt them for culture experiments, the cells were transferred to flasks containing 100 mL of 1/2 S&Scaron; medium (Přibyl *et al.*, 2015) and pre-cultured at 80 µmol m^−^^2^ s^−1^ PAR at room temperature for a few days until appropriate amounts of biomass were reached. Cultures were shaken manually several times a day. Strains for experiments with exogenously applied phytohormones were then transferred into bubble columns of 3·8 cm inner diameter (Kavalierglass, Prague, Czech Republic) at a continuous incident light intensity of 230 µmol m^−^^2^ s^−1^ PAR at room temperature and bubbled with 2 % CO_2_ (v/v) in air. Cultures were collected in the early stationary growth phase (after 10–14 d) and subsequently used for further experiments. Strains for phytohormone profiling were cultured in the same flasks and under the same culture conditions as given above for another 3–4 weeks to reach the early stationary growth phase. Before identification and quantification of CKs and auxins, cyanobacterial and algal suspension cultures were centrifuged for 20 min at 20 000 *g* and 4 °C (Beckman Coulter, Palo Alto, CA, USA). Subsequently, supernatants were removed by pipetting and pellets were immediately frozen in liquid nitrogen.

### Endogenous cytokinin and auxin profiles in cyanobacterial and algal species

Endogenous CKs and auxins were extracted from homogenized samples of cyanobacteria and algae (0·108–0·251 g fresh weight) according to a previously described method (Dobrev and Kamínek, 2002). Determination and quantification of CKs and auxins were performed with a high-performance liquid chromatography (HPLC) system (Ultimate 3000, Dionex) coupled to a hybrid triple quadrupole/linear ion trap mass spectrometer (3200 Q TRAP, Applied Biosystems) using a multilevel calibration graph with [^2^H]-labelled internal standards, as described previously (Dobrev *et al.*, 2005; Djilianov *et al.*, 2013; Žižková *et al.*, 2015). Detection of 2-methylthio derivatives of iP, *trans*Z and their ribosides was set up in a selected reaction-monitoring mode.

### Metabolism of exogenously applied [^3^H]*trans*Z, [^3^H]IAA, [^3^H]NAA and [^3^H]2,4-D in cyanobacterial and algal cells

Metabolic profiles of exogenously applied radiolabelled *trans*Z ([^3^H]*trans*Z, 1·5 × 10^6^ d.p.m. µL^−1^) added to the culture medium at final concentration 1 µm or 20 nm were determined in one cyanobacterial strain (*Chroococcus minutus*, CCALA 55) and three strains of algae (*Chlorococcum elbense*, CCALA 282; *Klebsormidium flaccidum*, CCALA 786; *Scenedesmus obliquus*, CCALA 454) cultured in liquid media as described previously and regenerated overnight in a culture room (16 h light/8 h dark, 20 °C) under continuous shaking (120 rpm, orbital diameter 20 mm). Cells and media (200 mg fresh weight and 10 mL per sample) were collected separately by filtering through Whatman GF/C glass fibre filters (5 cm diameter) at time points 0, 0·5, 1, 2, 4 and 24 h.

Similarly, radiolabelled [^3^H]IAA, [^3^H]NAA or [^3^H]2,4-D was added to the culture medium of four algal strains (*Chlorococcum ellipsoideum*, CCALA 283; *Stigeoclonium helveticum*, CCALA 868; *Scenedesmus obliquus*, CCALA 454; and *Microthamnion kuetzingianum*, CCALA 368) at a final concentration of 20 nm. Cells and media were collected separately by filtering through Whatman GF/C glass fibre filters (5 cm diameter) at time points 0, 1, 2 and 6 h.

Radiolabelled metabolites of *trans*Z and auxins were separately analysed by HPLC coupled to an on-line radioactivity detector under the same analytical conditions, with the exception of the different gradients. A Luna HPLC column C18(2) (150 × 4·6 mm, 3 µm; Phenomenex, Torrance, CA, USA), was used; mobile phase A was 40 mm CH_3_COONH_4_ (pH 4) and mobile phase B was CH_3_CN/CH_3_OH, 1/1 (v/v), and flow rate was 0·6 mL min^−1^. The linear gradient programme for *trans*Z metabolites was 10–40 % B for 12 min, 40–100 % B for 1 min, 100 % B for 2 min and 100–10 % B for 1 min. The linear gradient programme for auxin metabolites was 30–50 % B for 10 min, 50–100 % B for 1 min, 100 % B for 2 min and 100–30 % B for 1 min. The column eluate was monitored with a Ramona 2000 on-line radioactivity detector (Raytest, Straubenhardt, Germany) after on-line mixing with three volumes (1·8 mL min^−1^) of liquid scintillation cocktail (Flo-Scint III, Perkin Elmer Life and Analytical Sciences, Shelton, CT, USA). The radioactive metabolites were identified on the basis of comparison of their retention times with authentic standards. Results of auxin metabolic profiles are presented as total integrated area of chromatogram plots normalized to the equalization of total accumulated radiolabel.

### Cytokinin oxidase/dehydrogenase *in vitro* assay

The enzyme preparations were extracted and partially purified using the method described by Motyka *et al.* (2003). The CKX activity was determined by *in vitro* assays based on the conversion of [2-^3^H]-labelled CKs ([^3^H]*trans*Z, [^3^H]*cis*Z and [^3^H]iP) to [^3^H]adenine. Separation of the substrate from the product of the enzyme reaction was achieved by HPLC as described by Gaudinová *et al.* (2005). The CKX activity was determined in duplicate in two independent experiments.

### Growth of *Scenedesmus obliquus*

Pre-cultures of microalgae *Scenedesmus obliquus* were diluted with 1/2 S&Scaron; fresh medium (Přibyl *et al.*, 2015) to obtain the cell density of 1·0–1·5 × 10^6^ cells mL^−1^ (around 0·15–0·20 g L^−1^ dry weight). The cell density was determined using a Bürker counting chamber (Hecht-Assistent, Sondheim, Germany); at least 400 cells were counted. The culture was cultured in bubble columns of 3·8 cm inner diameter (Kavalierglass, Prague, Czech Republic) at a continuous incident light intensity of 500 µmol m^−^^2^ s^−1^ PAR at 30 ±0·5 °C and bubbled with 2 % CO_2_ (v/v) in air. The dilution procedure was repeated twice each 24 h and the resulting inoculum was used for batch-culture experiments in an initial volume of 150 mL under the same conditions as described above for 14 d. During culture, samples were taken regularly for analysis following the replenishment of water evaporated from the bubble columns. Growth was determined gravimetrically based on increased cell dry weight as follows: culture samples (1 5 mL) were centrifuged (10 000 *g*, 8 min) in pre-weighed microtubes and the sediment was dried at 105 °C. The cell density was quantified using a Bürker counting chamber (Hecht-Assistent, Sondheim, Germany); at least 600 cells were counted for each sample. Samples for CK analyses were collected at the same intervals; biomass was separated from the growth medium by centrifugation (10 000 *g*, 8 min) and both biomass and medium were immediately frozen in liquid nitrogen.

### Endogenous cytokinin profiles during *Scenedesmus obliquus* growth

The frozen pellets (as described above) used for determination of CKs were freeze-dried (Scanvac CoolSafe 110-4, Fisher Scientific) in a vacuum (Savant™ SPD 121P SpeedVac™ Concentrator, Thermo Scientific™) for 7 h. For analysis of free CKs, *Scenedesmus* *obliquus* samples (5 mg dry weight of each) were homogenized under liquid nitrogen, extracted in modified Bieleski buffer (methanol/water/formic acid, 15/4/1, v/v/v) containing 0·2 pmol of [^2^H]- or [^13^C]-labelled CK free bases/ribosides/*N*-glucosides and 0·5 pmol of [^2^H]-labelled CK-*O*-glucosides/nucleotides (Novák *et al.*, 2003, 2008), and then purified using two solid-phase extraction columns, the C18 octadecylsilica-based column and the MCX column (Dobrev and Kamínek, 2002). Analytes were eluted by two-step elution using a 0·35 m NH_4_OH aqueous solution and 0·35 m NH_4_OH in 60 % (v/v) MeOH. Levels of CKs were determined by ultrahigh-performance liquid chromatography-electrospray tandem mass spectrometry (UHPLC-MS/MS) with stable isotope-labelled internal standards as a reference (Svačinová *et al.*, 2012).

Extraction and purification of tRNA were performed according to a protocol described by Maass and Klämbt (1981) including modifications described by Stirk *et al.* (2011). Aliquots of extracted total tRNA were hydrolysed with 2 m KOH overnight and dephosphorylated using alkaline phosphatase. Addition of internal standards (0·2 pmol of each [^2^H]-labelled CK riboside), sample purification on a mixed-mode cation exchange (MCX) column and tRNA-bound CK quantification was performed by UHPLC-MS/MS as described above. 2-Methylthio derivatives of tRNA-bound isoprenoid CKs were analysed with an HPLC-MS/MS system as described previously (Tarkowski *et al.*, 2010). The extraction and purification of *Scenedesmus* *obliquus* samples were carried out in two technical replicates for each biological replicate.

### Presentation of results

Each evaluation was carried out in duplicate in two or three independent experiments. The results are expressed as mean values and standard deviations of the means in the figures and/or tables.

## RESULTS

### Selection of cyanobacterial and algal species

In order to extend our current knowledge concerning CK and auxin metabolism in non-vascular organisms, a search for suitable cyanobacterial and algal candidates was performed with respect to their distinct evolutionary history, taxonomic position and habitat requirements. The complete list and abbreviations of all representatives analysed for CK and auxin profiles, including cyanobacteria and the major lineages brown algae (Ochrophyta), red algae (Rhodophyta) and green algae (Chlorophyta and Streptophyta), is shown in Table S1.

The position of selected taxa within a simplified phylogenetic tree based on different data sources from the whole chloroplast genome and nuclear rDNA (Riisberg *et al.*, 2009; Ruhfel *et al.*, 2014) is demonstrated in Fig. 1. In addition to Prokaryota, represented by three cyanobacterial species (*Chroococcus minutus*, *Phormidium animale* and *Nostoc microscopicum*), three ochrophytes (*Tribonema aequale*, *Bumilleriopsis filiformis* and *Vischeria helvetica*), two red algae (*Porphyridium purpureum* and *Rhodella violacea*) and two major lineages of green algae referred to the chlorophyte and charophyte/streptophyte clades (see Table S1) were included in the analysed set of eukaryotic organisms. Nine Chlorophyta species belonging to three major groups (Chlorophyceae, Ulvophyceae and Trebouxiophyceae) and two Streptophyta species (*Actinotaenium curtum* and *Klebsormidium flaccidum*) as representatives of evolutionarily more advanced organisms closely related to vascular plants were selected within the green algae for analyses of CK and auxin spectra (Fig. 1, Table S1). Another Chlorophyta species, *Chlorococcum elbense*, was chosen for metabolic studies only. To summarize, the set of analysed samples was representative enough to enable a very comprehensive survey of the regulation of CK and auxin metabolism in photoautotrophic microorganisms of different phylogenetic origin.

### Cytokinin profiles in cyanobacteria and algae differ substantially from those in vascular plants

**Fig. 2.**
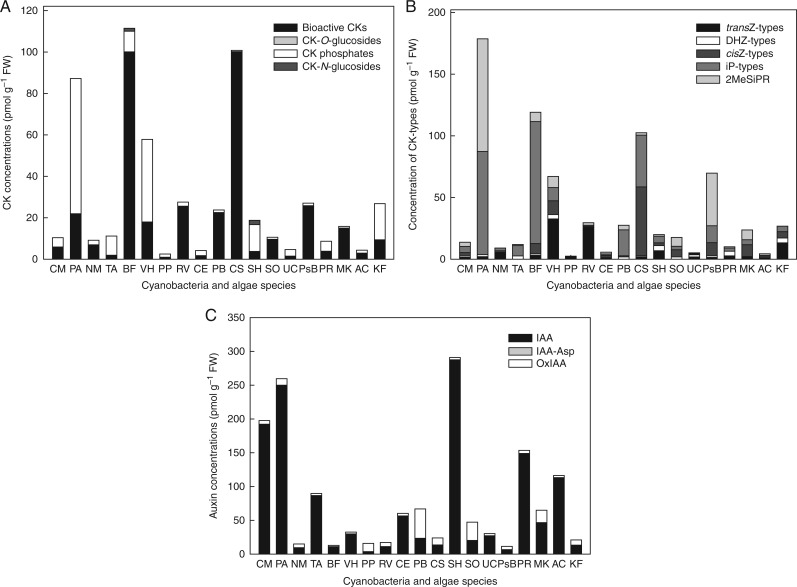
Endogenous cytokinin and auxin profiles in selected cyanobacterial and algal species. Cytokinin profiles were determined in the early stationary growth phase and are based on conjugation status/physiological function (A) and the chemical structure of the purine ring (B). Endogenous levels of both cytokinins and auxins (C) are expressed in pmol g^−1^ FW. Abbreviations of selected cyanobacterial and algal representatives are given in Table S1.

In analogy to vascular plants, a wide spectrum of isoprenoid CKs was detected in both cyanobacteria and algae. Total CK levels in different species varied from picomoles per gram fresh weight (FW) (e.g. *Porphyridium purpureum*, 2·53 pmol g^−1^ FW) to hundreds of picomoles (e.g. *Phormidium animale*, 178·55 pmol g^−1^ FW) (Fig. 2, Supplementary Data Table S2). Bioactive CKs (free bases and ribosides) and CK phosphates were the prevalent CK forms, being present in concentrations from 1·01 pmol g^−1^ FW (*Porphyridium purpureum*) to 100·19 pmol g^−1^ FW (*Chlamydomonas segnis*) and from 0·56 pmol g^−1^ FW (*Chlamydomonas segnis*) to 65·36 pmol g^−1^ FW (*Phormidium animale*), respectively. On the other hand, CK-*N*-glucosides were not detected or detected in only trace amounts throughout the set of analysed species. Similarly, CK-*O*-glucosides occurred only in minute concentrations or were absent in the tested samples (Fig. 2A, Table S2). In general, the iP-, *cis*Z- and *trans*Z-type CKs predominated in all analysed cyanobacteria and algae, while DHZ types contributed only insignificantly (with concentrations ranging from 0·35 to 3·95 pmol g^−1^ FW) to the total CK pool. Interestingly, whereas monophosphate forms of iP, *trans*Z and DHZ were relatively abundant in most of the species, *cis*Z was found only in biologically active free-base and riboside forms in all of the analysed taxa. In 13 (out of 19) analysed taxa, the levels of *cis*Z and its riboside exceeded those of corresponding *trans*Z counterparts, in most of them being more than at least 3-fold higher (Fig. 2B, Table S2). Interestingly, 2-methylthio-*N*^6^-(Δ^2^-isopentenyl)adenosine (2MeSiPR) was detected in moderate or high concentrations in almost all of the analysed samples, representing a predominant metabolite in some Cyanobacteria (*Chroococcus minutus*, *Phormidium animale*) and Chlorophyta (*Chlorococcum ellipsoideum*, *Pseudendoclonium basiliense*, *Scenedesmus obliquus*, *Microthamnion kuetzingianum*) species (Fig. 2B, Table S2). In summary, the profiles of endogenous CKs in selected cyanobacteria and algae revealed a predominance of biologically active and phosphate CK forms and relatively small amounts of CK-*O*- and *N*-glucosides. Moreover, it is demonstrated that *cis*Z-type CKs and 2MeSiPR substantially contribute to the overall CK pool, indicating the existence of diverse metabolic pathways in cyanobacteria and algae compared with vascular plants.

### Spectra of auxin metabolites in cyanobacteria and algae are rather narrow

In all of the selected cyanobacteria and algae, the screen of endogenous CKs was supplemented by analysis of endogenous indole auxin levels. In the whole spectrum of analysed species and biological samples, the auxins IAA, its primary catabolite OxIAA and an amino acid conjugate, IAA-Asp, were detected. The total auxin concentrations ranged from 10·93 to 290·69 pmol g^−1^ FW, with the lowest as well as the highest endogenous levels in Chlorophyta species *Pseudendoclonium basiliense* and *Stigeoclonium helveticum*, respectively (Fig. 2C, Supplementary Data Table S3). The main auxins were represented by free IAA (occurring in concentrations from 3·26 to 287·57 pmol g^−1^ FW; *Porphyridium purpureum* and *Stigeoclonium helveticum*, respectively) and OxIAA (ranging from 1·78 to 43·54 pmol g^−1^ FW; *Bumilleriopsis filiformis* and *Protosiphon botryoides*, respectively) whereas concentrations of IAA-Asp were close to the detection limit in all of the tested species (Fig. 2C, Table S3). Taking these results together, endogenous free IAA and OxIAA evidently represent predominant indole auxin forms in the selected cyanobacterial and algal samples.

### Cyanobacteria and algae metabolize exogenously applied [^3^H]*trans*Z

**Fig. 3.**
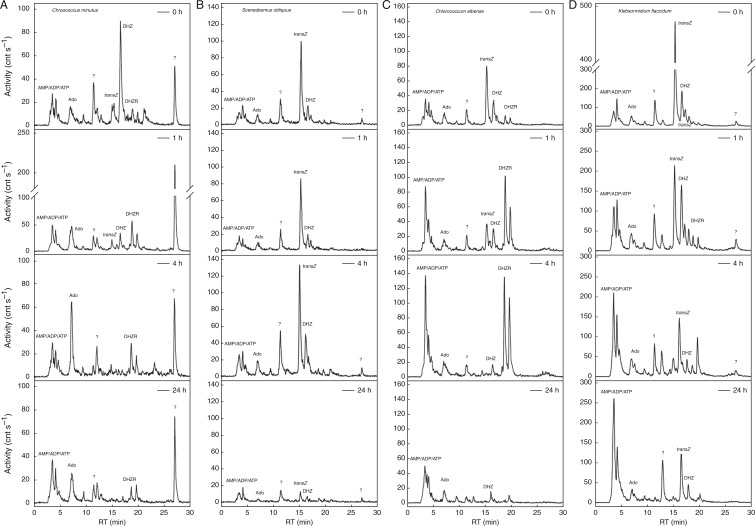
Metabolism of exogenously applied [^3^H]*trans*Z in cells of selected cyanobacterial and algal species. Radiolabelled [^3^H]*trans*Z was applied to cultures of cyanobacteria *Chroococcus minutus* (A) and algae *Scenedesmus obliquus* (B), *Chlorococcum elbense* (C) and *Klebsormidium flaccidum* (D) in the early stationary growth phase. The peaks represent distributions of radioactivity associated with individual metabolites in the cells 0, 1, 4 and 24 h after [^3^H]*trans*Z application. The products of [^3^H]*trans*Z metabolism were analysed by an HPLC system coupled to an online radioactivity detector. cnt, counts; RT, retention time; Ado, adenosine.

In order to study the regulation of CK levels in non-vascular plants and to compare it with regulation in vascular plants, radiolabelled *trans*Z was exogenously applied to cultures of cyanobacteria (*Chroococcus* *minutus*) and selected algae (*Chlorococcum elbense*, *Klebsormidium flaccidum* and *Scenedesmus obliquus*). Following [^3^H]*trans*Z treatment, some relatively rapid metabolic changes in cultured cells were observed as early as after 1 h of incubation (Fig. 3A–D). After 4 h of incubation, the degradation products adenine [retention time (RT) = 4·15 min] and adenosine (RT = 7·12 min) together with other substances, such as AMP/ADP/ATP (RT = 4·0 min), DHZ (RT = 16·5 min) and its riboside (RT = 18·5 min), were found, based on their retention times on HPLC. After 24 h, almost complete conversion of [^3^H]*trans*Z was apparent in *Chroococcus minutus*, *Scenedesmus obliquus* and *Chlorococcum elbense* (Fig. 3A–C), which was in contrast to *Klebsormidium flaccidum*, where a relatively high amount of [^3^H]*trans*Z persisted (about one-fourth of the initial amount; Fig. 3D). Interestingly, relatively high concentrations of rapidly formed, unknown metabolites were detected after [^3^H]*trans*Z treatment in all four analysed species (Fig. 3A–D).

Metabolism of [^3^H]*trans*Z was also followed in the culture medium of *Chroococcus minutus* and *Klebsormidium flaccidum.* After incubation (4 and 24 h), [^3^H]*trans*Z was largely converted in the *Klebsormidium* *flaccidum* medium with subsequent accumulation of DHZ and several unidentified metabolites (Supplementary Data Fig. S1). Similarly, a decline in [^3^H]*trans*Z 24 h after its supply was recorded and some unidentified metabolites were detected in the medium of *Chlorococcus minutus* (data not shown). Altogether, our data strongly suggest a potential intra- as well as extracellular regulation of CK status in both cyanobacterial and algal cells.

The intense *in vivo* formation of adenine and adenosine as products of [^3^H]*trans*Z metabolism in selected cultures raises the question of the potential involvement of CKX activity in the degradation of CKs in cyanobacteria and algae. To investigate this subject, degradation of [^3^H]*trans*Z, [^3^H]*cis*Z and [^3^H]iP by the CKX activity isolated from crude protein preparations of eight species, including Cyanobacteria, Rhodophyta, Ochrophyta and Chlorophyta, was determined. *In vitro* enzymatic assays performed at two pH values, pH 7·0 and pH 8·5 (Supplementary Data Figs S2 and S3 for [3H]iP; data not shown for ^3^[H]*trans*Z and [^3^H]*cis*Z) revealed no CKX activity for any of the tested samples. Interestingly, an unknown metabolite (RT = 2·5 min) was formed *in vitro* as a product of radiolabelled CK substrates in all analysed samples (Figs S2 and S3; data not shown). To summarize our finding, in spite of the intense *in vivo* conversion of [^3^H]*trans*Z to adenine and/or adenosine, the assumed CKX activity was not detected.

### Exogenously applied [^3^H]IAA is gradually metabolized in algal cell cultures

**Fig. 4.**
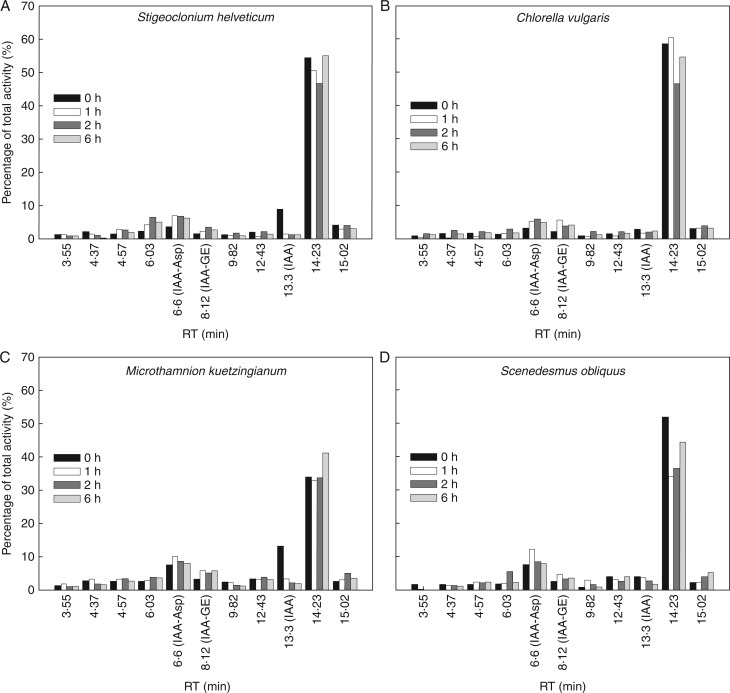
Metabolism of exogenously applied [^3^H]IAA in the cells of selected algal species. Radiolabelled [^3^H]IAA was applied to cultures of Chlorophyta species *Stigeoclonium helveticum* (A), *Chlorella vulgaris* (B), *Microthamnion kuetzingianum* (C) and *Scenedesmus obliquus* (D) in the early stationary growth phase. Bars represent distribution of radioactivity associated with individual metabolites in cells 0, 1, 2 and 6 h after [^3^H]IAA application. Products of [^3^H]IAA metabolism were analysed by HPLC coupled to an on-line radioactivity detector. Values are percentages of total extracted radioactivity in cells. RT, retention time.

**Fig. 5.**
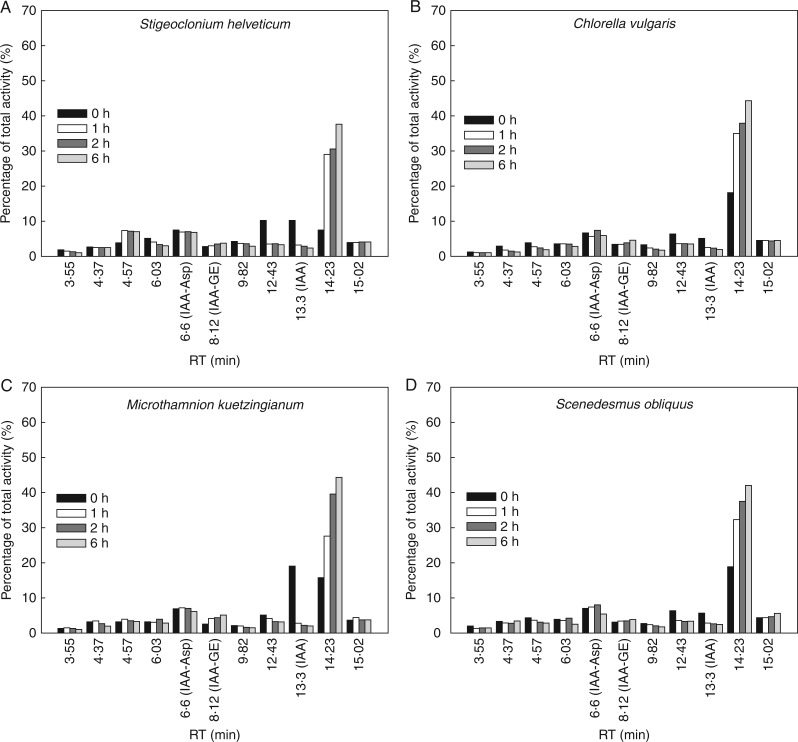
Metabolism of exogenously applied [^3^H]IAA in culture media of selected algal species. Radiolabelled [^3^H]IAA was exogenously applied to cultures of Chlorophyta species *Stigeoclonium helveticum* (A), *Chlorella vulgaris* (B), *Microthamnion kuetzingianum* (C) and *Scenedesmus obliquus* (D) in the early stationary growth phase. Bars represent distribution of radioactivity associated with individual metabolites in media 0, 1, 2 and 6 h after [^3^H]IAA application. Products of [^3^H]IAA metabolism were analysed by HPLC coupled to on-line radioactivity detector. Values are percentages of total extracted radioactivity in the media. RT, retention time.

In order to characterize more precisely the auxin metabolism in non-vascular plants, radiolabelled [^3^H]IAA was exogenously applied to cultures of the four Chlorophyta species *Stigeoclonium helveticum, Chlorella vulgaris*, *Microthamnion kuetzingianum* and *Scenedesmus obliquus.* In all of the analysed green algae, exogenously applied [^3^H]IAA was gradually metabolized in both cells (Fig. 4) and media (Fig. 5). During 6 h of [^3^H]IAA treatment, formation of IAA-Asp (RT = 6·6 min), IAA-GE (RT = 8·12 min) and eight other unidentified metabolites with a major product with RT 14·23 min was detected. To compare auxin metabolic profiles in algal cells after addition of synthetic radiolabelled auxin compounds, [^3^H]NAA and [^3^H]2,4-D were applied. Surprisingly, in comparison with numerous conversions of exogenously applied IAA, no significant effect on substrate metabolism in selected algal cells and media was observed (data not shown), probably due to missing metabolic pathway(s) for such unnaturally occurring substrates or an inability to transport them inside the cells.

### Cytokinin and auxin metabolite profiles are differently affected during *Scenedesmus obliquus* growth

**Fig. 6.**
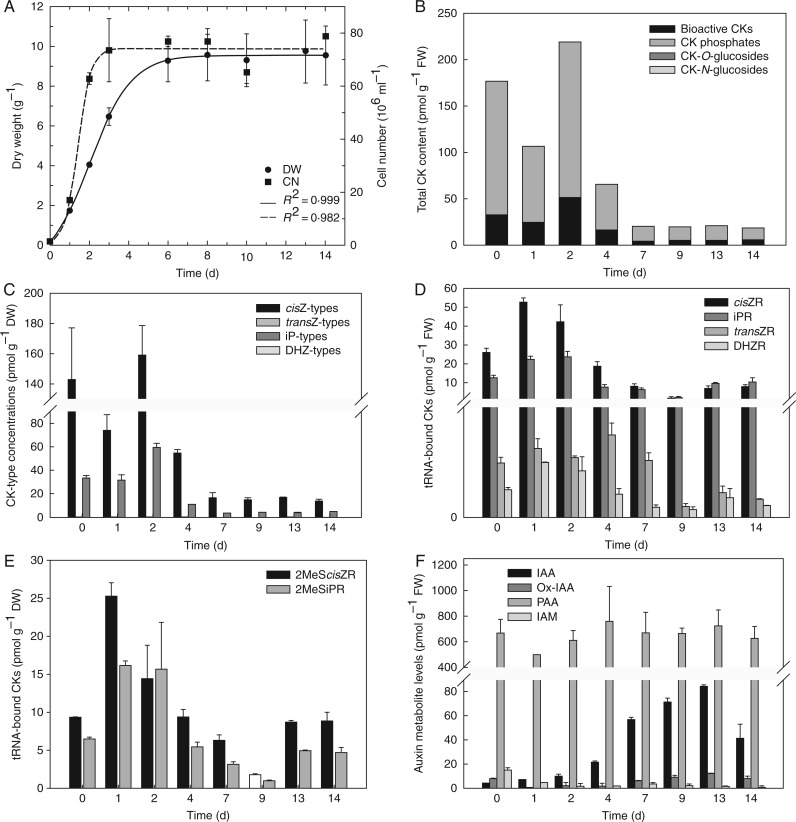
Growth characteristics and endogenous cytokinin and auxin profiles during the *Scenedesmus obliquus* growth cycle. The cycle was characterized by dry weight and cell number increase (A), variations in profiles of endogenous free cytokinins based on conjugation status/physiological function (B) and the chemical structure of the purine ring (C), as well as tRNA-bound cytokinins (D), including methylthio derivatives (E) and changes in the spectra and concentrations of endogenous auxins (F). Levels of free and tRNA-bound cytokinins and auxins are expressed in pmol g^−1^ dry weight.

With the aim of deciphering the regulation of CK and auxin homeostasis in non-vascular organisms, *Scenedesmus obliquus* was used as a representative green alga of the chlorophyte clade because of its relatively easy culture. The cellular growth and concentrations of CKs (both free and tRNA-bound) and auxins were recorded during the course of *Scenedesmus* *obliquus* culture (Fig. 6).

After a short lag phase, a rapid increase in cell number was detected, reaching the stationary growth phase after 2–3 d of culture. Biomass dry weight showed a similar pattern, but with a substantial delay in entering the stationary growth phase (Fig. 6A). Within the exponential and linear phases of cellular growth (0–4 d), the total free CK concentration was at least 3-fold higher than in the stationary phase (7–14 d). Among CK groups, CK phosphates represented the predominant CK forms (67–81 % of the total), whereas the rest of the CK pool comprised mainly bioactive free bases and ribosides. CK-*O*-glucosides were found at very low concentrations, close to the detection limit (ranging from 0·09 to 0·58 pmol g^−1^ FW) and CK-*N*-glucosides were not detected at all (Fig. 6B). Remarkably, the highest levels of CK phosphates and bioactive CKs were reached at day 2 (168·48 ± 12·56 and 50·50 ±3·06 pmol g^−1^ FW, respectively) while at least 9-fold decreased contents of both CK groups (12·38 ± 1·90 and 5·29 ±0·26 pmol g^−1^ FW, respectively) were observed in the stationary phase of *Scenedesmus obliquus* growth (Fig. 6B). The CK profiling also revealed that endogenous levels of *cis*Z and its metabolites considerably exceeded concentrations of other CK types during *Scenedesmus* *obliquus* growth and that iP types represented the second most abundant CK forms (Fig. 6C).

To characterize the contribution of the tRNA biosynthetic pathway to the CK pool in the course of *Scenedesmus* *obliquus* growth, tRNA-bound CKs were further assessed. The profile of individual CK ribosides derived from tRNA revealed a dominant proportion of *cis*Z-9-riboside (*cis*ZR) and *N*^6^-(Δ^2^-isopentenyl)adenosine (iPR) during the first 7 d of *Scenedesmus* *obliquus* culture (Fig. 6D). The highest concentration of *cis*ZR (52·45 ± 2·42 pmol g^−1^ dry weight) was reached at day 1 of the exponential phase of growth, whereas its lowest level was found at the beginning of the stationary phase (day 9; 2·11 ± 0·56 pmol g^−1^ dry weight). Interestingly, very low concentrations of *trans*Z-9-riboside (*trans*ZR) and DHZ-9-riboside (DHZR), varying from 0·07 ± 0·03 to 0·75 ± 0·10 pmol g^−1^ dry weight, were detected during the whole *Scenedesmus* *obliquus* growth cycle (Fig. 6D). Consistently, a prevalence of *cis*ZR and iPR was found also when concentrations of tRNA-bound CKs were recalculated in relation to milligrams of isolated tRNA instead of dry weight (Supplementary Data Table S4). In addition, determination of tRNA-bound 2-methylthio derivatives revealed the presence of 2-methylthio-*cis*Z-9-riboside (2MeS*cis*ZR) and 2MeSiPR during the whole *Scenedesmus obliquus* growth cycle (Fig. 6E). Both 2MeS*cis*ZR and 2MeSiPR were detected at the highest concentrations after day 1 of the algal cell growth cycle (reaching 26·10  ± 1·84 and 16·02  ± 0·55 pmol g^−1^ dry weight, respectively), followed by a continuous decline until day 9 and subsequently by an obvious increase at the end of the stationary phase (Fig. 6E). When related to pmol mg^−1^ tRNA, the contents of 2MeS*cis*ZR and 2MeSiPR showed variations, especially during the exponential phase of growth (Table S4).

To get insight into the complexity of phytohormone regulation during 14 d of *Scenedesmus* *obliquus* cell growth, the auxin metabolite profile was also determined (Supplementary Data Table S5). The content of IAA gradually increased from the start of the experiment, reaching its maximum after 13 d of culture (84·9 ± 0·96 pmol g^−1^ FW) and then decreasing rapidly and noticeably to approximately one-half of this value (14 d; 41·4 ± 11·65 pmol g^−1^ FW) (Fig. 6F). Concentrations of auxin precursors IAM and IAN were considerably lower than those of free IAA, exhibiting moderate variation in their levels during *Scenedesmus* *obliquus* growth. The auxin catabolite OxIAA reached its maximum at a stage similar to IAA (at 13 d), whereas the endogenous conjugated forms IAA-Asp and OxIAA-GE were detected at very low concentrations within the exponential and linear growth phases (Table S5). Surprisingly, the main auxin metabolite was PAA, with an amount about one order of magnitude higher than IAA, but without showing any significant dynamic changes during the culture period (Fig. 6F).

Taken together, our data demonstrate a dynamic regulation of CK and auxin homeostasis during the *Scenedesmus* *obliquus* cell growth cycle. The enhanced concentration of CKs in cells during the phase of their intensive growth is mainly due to an enormous amount of CK phosphates, especially those of *cis*ZR (*cis*ZRMP) and iPR (iPRMP). Substantial amounts of *cis*ZR, iPR and their 2-methylthioderivatives seem to be delivered mainly through the tRNA biosynthetic pathway (Fig. 6). In contrast, concentrations of auxins (mainly of biologically active IAA) gradually increase from the exponential to the linear growth phase. In summary, the distinct proportions of CK and auxin profiles during *Scenedesmus* *obliquus* growth may indicate their different functioning and physiological consequences in algal cells.

## DISCUSSION

It has been postulated that physiological and structural changes in the metabolism of vascular plants developed progressively with the transition from an aqueous to a gaseous environment (Kenrick and Crane, 1997). Thus, the components of the CK and auxin metabolic pathways identified in vascular plants probably arose from pre-existing elements of bacterial primary metabolism via endosymbiosis and horizontal gene transfer (Spíchal, 2012; Yue *et al.*, 2014). However, in contrast to the relatively well-characterized regulation of CK and auxin homeostasis via different metabolic pathways in vascular plants (e.g. Normanly, 2010; Korasick *et al.*, 2013; Kieber and Schaller, 2014), data concerning the regulatory mechanisms of both phytohormone levels in evolutionarily older non-vascular organisms are rather limited. To attempt to fill this gap, selected cyanobacterial and algal species belonging to divergent evolutionary lineages (Fig. 1, Table S1) were screened and analysed for CK and auxin profiles and metabolic pathways involved in their homeostasis control.

For all cyanobacterial and algal species used in this study, some common traits regarding the profiles of isoprenoid CK derivatives were found. In general, bioactive CKs and CK phosphates represented the prevailing CK forms, in contrast to CK-*O*- and *N*-glucosides, which occurred only in moderate or hardly detectable concentrations (Fig. 2A, Table S2). Cytokinin spectra similar to those found here have been reported previously by Stirk *et al.* (2013) and Yokoya *et al.* (2010), but there are also a few exceptions demonstrating higher proportions of CK-*O*-glucosides in some microalgal and macroalgal strains than shown in our study (Stirk *et al.*, 2003; Ördög *et al.*, 2004; Lu *et al.*, 2014). Among the CK derivatives detected, 2MeSiPR was present in most tested species (Table S2), suggesting its tRNA origin, as reported by e.g. Prinsen *et al.* (1997). Additionally, the enzyme catalysing the methiolation of CK derivatives was discovered in chloroplasts of the light-grown photosynthetic protozoan *Euglena gracilis* (Swaminathan and Bock, 1977). Thus, it can be assumed that tRNA degradation is an essential source of CKs, representing a predominant biosynthetic pathway of CKs in evolutionarily older non-vascular organisms, such as cyanobacteria and algae.

Among the bioactive CKs, free bases of iP, *cis*Z and in some species also *trans*Z were found as major CK forms. Their ribosides were detected mostly in lower amounts, and *trans*ZR was completely missing in cyanobacteria and just sporadically detected in algae (Table S2). Regarding zeatins, *cis* forms were more common than *trans* forms; the contents of *cis*Z and its riboside exceeded those of *trans*Z counterparts in 13 (out of 19) cyanobacterial and algal samples. This prevalence of *cis*Z over *trans*Z types corresponds well with findings reported by other authors (Ördög *et al.*, 2004; Stirk *et al.*, 2002, 2003, 2013). Based on these data, we suggest that the function of CK-*N*-glucosides (i.e. deactivation or reduction of biological activity) in cyanobacteria and algae may be, at least partially, substituted by *cis*Z types, which represent prevailing and generally less active forms compared with *trans*Z types (Gajdošová *et al.*, 2011).

As expected, CK status in cyanobacteria and algae was not steady and could be dramatically affected by internal as well as external factors. In our study, substantially lower concentrations of isoprenoid CKs than those published by Stirk *et al.* (2013) were found in the green algae *Chlorococcum ellipsoideum*, *Protosiphon botryoides* and *Klebsormidium flaccidum*. The differences could be mainly due to the distinct growth phases of the analysed species; whereas the measurements of our samples were performed in the early stationary growth phase, the analyses by Stirk’s group were done in the exponential phase of growth. Additionally, environmental factors might affect the CK profiles of cyanobacteria and algae. For instance, the CK spectra were found to be dependent on water temperature in the Chlorophyta seaweed *Ulva* sp. (Stirk *et al.*, 2003), and the effects of light during the cell cycle of *Chlorella minutissima* (Stirk *et al.*, 2011) and nitrogen depletion in *Nannochloropsis oceanica* (Lu *et al.*, 2014) on CK profiles have been reported.

The present study also showed a wide concentration range of auxins (free IAA, OxIAA and IAA-Asp) in selected cyanobacterial and algal species (Table S3). This is consistent with previously published results demonstrating wide variation in concentrations of IAA and other auxin metabolites, such as IAA-Glu, IAA-Leu and IAM, in cyanobacteria and algae (Tarakhovskaya *et al.*, 2007; Hussain *et al.*, 2010; Yokoya *et al.*, 2010; Yokoya and Yoneshigue-Valentin, 2011). The auxin derivatives OxIAA and IAA-Asp detected in our study represent metabolic products of two major catabolic pathways of IAA in plants. The first is IAA oxidation to the primary catabolite OxIAA (Kai *et al.*, 2007*a*) and the second is an amino acid conjugation of IAA with aspartate, leading to the formation of IAA-Asp (Ljung *et al.*, 2002; Ludwig-Müller, 2011). Our data revealed that free IAA and OxIAA were the main auxins, while IAA-Asp occurred only at concentrations close to the detection limit, if present at all (Table S3), indicating a higher relevance of the oxidative than the conjugative pathway in IAA catabolism in cyanobacteria and algae.

Fast regulation of bioactive CK and auxin pools after [^3^H]*trans*Z and [^3^H]IAA treatments strongly suggests that cyanobacteria and algae possess effective mechanism(s) controlling CK and auxin homeostasis in cells. Using exogenously applied [^3^H]*trans*Z, a swift conversion within 1 h of incubation, especially in *Chroococcus minutus* and *Chlorococcum elbense*, was observed (Fig. 3A, C). In spite of the extensive formation of radiolabelled adenine and/or adenosine following [^3^H]*trans*Z treatment, no CKX activity was detected *in vitro* for any of the species analysed (Figs S2 and S3). This is in accordance with recently published data demonstrating only a very sporadic detection of homologous CKX sequences in algae (Lu *et al.*, 2014) and no detectable enzymatic activity of NoCKX1 in cyanobacterium *Nostoc* sp. PCC 7120 (Frébortová *et al.*, 2015). Notably, the occurrence of [^3^H]*trans*Z metabolite peaks with retention times corresponding to DHZ and DHZR (Fig. 3) suggests a potential involvement of zeatin reductase activity, detected so far only rather rarely in some vascular plant species (Martin *et al.*, 1989; Gaudinová *et al.*, 2005).

It was previously found that feeding *Arabidopsis* seedlings with IAA led to increased accumulation of IAA-Asp, IAA-Glu, IAA-GE, OxIAA and OxIAA-GE (Kai *et al.*, 2007*b*), most likely due to the activation of multiple metabolic pathways in response to the exogenous IAA supply. Consistently with these results, our findings in selected algal species (*Scenedesmus obliquus*, *Chloroccocum elbense*, *Stigeoclonium helveticum* and *Microthamnion kuetzingianum*) revealed a gradual metabolization of exogenously applied [^3^H]IAA to IAA-Asp and IAA-GE as two major products. Detection of eight more unidentified metabolites in both cells and medium may indicate the existence of other, probably unknown IAA metabolic pathway(s) in green algae (Figs 4 and 5). Surprisingly, exogenous application of [^3^H]NAA or [^3^H]2,4-D did not have any significant effect on substrate metabolization (data not shown), although both of these synthetic auxins were shown to stimulate cell growth and to increase cell fresh and dry weight in *Chlorella pyrenoidosa* cultures (Czerpak *et al.*, 1994). It may be speculated that the absence of metabolization of [^3^H]NAA or [^3^H]2,4-D is due to non-functional transport mechanisms or absent metabolic pathways for rapid inactivation of these unnatural auxins.

The apparent correlation between *Scenedesmus obliquus* growth and levels of both CKs and auxins, as demonstrated in Fig. 6, suggests an indispensable role of the two phytohormones in algal cell division. Corresponding to the data of Stirk *et al.* (2014), *cis*Z and iP types mainly contributed to the total CK pool. Enhanced content of CK phosphates, particularly *cis*ZRMP and iPRMP, during the exponential and linear growth phases (0–3 d) indicated higher CK biosynthetic rates in algal cells in comparison with the stationary phase (Fig. 6B). Our data are fully compatible with the assumed roles of *cis*Z and iP nucleotides as immediate products in CK biosynthesis, in contrast to *trans*Z and DHZ nucleotides, formed particularly by side-chain modification (Takei *et al.*, 2004; Hwang and Sakakibara, 2006; Kieber and Schaller, 2014). Screening of tRNA-bound CKs during *Scenedesmus* *obliquus* growth also revealed a prevalence of *cis*ZR and iPR, contrary to *trans*ZR a DHZR (Fig. 6E). iPR and *cis*ZR have also been reported previously as predominantly tRNA-bound as well as free CK forms in several cyanobacterial and microalgal species by Šimura *et al.* (2014). The significance of *cis*Z-type CKs in the *Scenedesmus* *obliquus* growth cycle demonstrated in our observations and their potential origin from the tRNA pathway suggested by recent phylogenetic studies, in which higher similarity of IPTs from cyanobacteria and microalgae related to tRNA-IPTs was reported (Frébortová *et al.*, 2015; Lu and Xu, 2015), is thus likely. The prevalence of tRNA-bound *cis*ZR and iPR, indicating an important involvement of a tRNA-dependent CK biosynthetic pathway, was also described in the moss *Physcomitrella patens* (Yevdakova *et al.*, 2008). However, a comparison between total concentrations of free CK forms and tRNA-bound CKs during *Scenedesmus* *obliquus* growth indicated a more pronounced production of CKs through *de novo* biosynthesis (Fig. 6C, D). Likewise, free *cis*Z- and iP-type CK forms were found in considerably higher concentrations than tRNA-bound CKs in vascular plants, such as oat, lucerne and maize, during their germination and early seedling establishment (Stirk *et al.*, 2012).

A subsequent metabolization of CK nucleotides in *Scenedesmus* *obliquus* culture is not clear because there was no increase in production of CK metabolites in the cells and the medium after stopping cell divisions (Fig. 6B and data not shown). Moreover, the genes involved in CK conjugation (*UGT*) and degradation (*CKX*) have not been functionally characterized in algae yet (Lu *et al.*, 2014; Frébortová *et al.*, 2015). Thus, it can be hypothesized that CK homeostasis during algal growth is controlled basically by modulation of the pool of CK ribotides functioning at least partially as storage metabolites. However, a more detailed characterization of this type of regulation needs further investigation.

In contrast to the CK profile, a gradual enhancement of IAA levels during the subculture period of *Scenedesmus* *obliquus* correlated with an increase in cell division during the exponential growth phase (2–8 d). Subsequently, IAA concentrations reached their maxima at the stationary phase (13 d) and then declined (Fig. 6F). These findings are consistent with those of Mähönen *et al.* (2014), who showed that higher auxin levels inhibit cell division and expansion but not cell differentiation in *Arabidopsis*. The presence of IAM, a precursor of auxin biosynthesis (reviewed by Woodward and Bartel, 2005; Korasick *et al.*, 2013), indicated an involvement of tryptophan-dependent pathway(s) in *Scenedesmus* *obliquus*. Detection of OxIAA in *Scenedesmus* *obliquus* in our experiment pointed to the importance of this auxin metabolic product (Östin *et al.*, 1998) in regulating bioactive IAA levels in non-vascular organisms in addition to vascular plants. Although the biological activity of PAA is generally lower compared with IAA (Sugawara *et al.*, 2015), the endogenous levels of PAA considerably exceeded those of IAA in *Scenedesmus* *obliquus* cells, which corresponds to data reported for numerous vascular plants [e.g. *Avena sativa* coleoptiles (Wightman and Lighty, 1982) and *Pisum sativum* roots (Schneider *et al.*, 1985)]. Moreover, PAA and IAA seem to function similarly by regulating auxin-responsive genes through the TIR1/AFB pathway, as demonstrated for *Arabidopsis* (Löbler and Klämbt, 1985), although direct interaction of PAA with TIR1/AFB and Aux/IAA proteins has yet to be investigated both in vascular plants (Shimizu-Mitao and Kakimoto, 2014) and in non-vascular plants. A relatively steady PAA content without any significant dynamic changes during the *Scenedesmus* *obliquus* growth cycle, however, raises the question of its spatiotemporal regulation in algae. To conclude, our results clearly demonstrate a diverse involvement of CKs and auxins during algal cell growth (Fig. 6), indicating specificities of their functioning in analogy to processes known for vascular plants (Coenen and Lomax, 1997).

## Conclusions

In summary, we present here an insight into the control of CK and auxin homeostasis in evolutionarily older non-vascular organisms, such as cyanobacteria and algae. The comprehensive screen of selected representatives for endogenous CK and auxin profiles reveals the prevalence of CK phosphates and *cis*Z-type CKs in the total CK pool, while in the auxinome free IAA and its primary catabolite OxIAA predominate. For both CKs and auxins, the conjugated forms were not found or were detected only in very low concentrations in cyanobacteria and algae. In contrast to vascular plants, CK downregulation by CKX activity was not observed in any of the tested species. Our data also demonstrate the occurrence and significance of CK methylthio derivatives, which indicates the importance of the tRNA pathway as a substantial source of CKs in cyanobacteria and algae. In addition, we show here the metabolic fate of exogenously applied [^3^H]*trans*Z and [^3^H]IAA in the selected taxa as well as changes in endogenous CK and auxin pools during the course of *Scenedesmus obliquus* culture, when high concentrations of non-indole PAA, exceeding those of indole auxins, were detected. Our results suggest the existence and operation of a complex network of metabolic pathways and regulation of activities of CKs and auxins in cyanobacteria and algae, apparently differing from those in vascular plants, and reveal a whole range of not yet answered questions regarding the control of phytohormone homeostasis in non-vascular plants.

## SUPPLEMENTARY DATA

Supplementary data are available online at www.aob.oxfordjournals.org and consist of the following. Table S1: list and abbreviations of cyanobacterial and algal species analysed for endogenous cytokinin and auxin profiles in this study. Table S2: endogenous cytokinin spectra and concentrations (in pmol g^−1^ FW) in selected cyanobacterial and algal species in the early stationary growth phase. Table S3: endogenous auxin spectra and concentrations (in pmol g^−1^ FW) in selected cyanobacterial and algal species in the early stationary growth phase. Table S4: endogenous spectra and concentrations of tRNA-bound cytokinins (pmol mg^−1^ tRNA) during the *Scenedesmus obliquus* growth cycle. Table S5: endogenous spectra and concentrations of auxins (pmol g^−1^ FW) during the *Scenedesmus obliquus* growth cycle. Figure S1: metabolism of exogenously applied [^3^H]*trans*Z in the culture medium of *Klebsormidium flaccidum* in the early stationary growth phase. Figure S2: metabolic conversion of [^3^H]*N*^6^-(Δ^2^-isopentenyl)adenine incubated *in vitro* with cytokinin oxidase/dehydrogenase preparations extracted and partially purified from selected cyanobacterial and algal species in the early stationary growth phase. Figure S3: metabolic conversion of [^3^H]*N*^6^-(Δ^2^-isopentenyl)adenine incubated *in vitro* with cytokinin oxidase/dehydrogenase preparations extracted and partially purified from selected cyanobacterial and algal species in the early stationary growth phase.

Supplementary Data
