# Supplementary material for: Control of cytokinin and auxin homeostasis in cyanobacteria and algae
Source: Ann Bot. 2016 Oct 5;119(1):151–66. doi: 10.1093/aob/mcw194 (PMC5218379; doi:10.1093/aob/mcw194)
Supplement: Supplementary Data [file supp_119_1_151__index.html]

Control of cytokinin and auxin homeostasis in cyanobacteria and algae — Supplementary Data 

# Control of cytokinin and auxin homeostasis in cyanobacteria and algae

## Supplementary Data

files

- Supplementary Data - zip file
