# Supplementary material for: Control of cytokinin and auxin homeostasis in cyanobacteria and algae
Source: Ann Bot. 2016 Oct 5;119(1):151–66. doi: 10.1093/aob/mcw194 (PMC5218379; doi:10.1093/aob/mcw194)
Supplement: Supplementary Data [file supp_mcw194_suppl_data.zip › aob-16427-s06.docx]

„Original Article”

**Control of cytokinin and auxin homeostasis in cyanobacteria and algae**

**Eva Žižková^1^, Martin Kubeš^1,2^, Petre I. Dobrev^1^, Pavel Přibyl^3^, Jan Šimura^2^, Lenka Zahajská^4^, Lenka Záveská Drábková^5^, Ondřej Novák^6^, Václav Motyka^1,*^**

^1^ Laboratory of Hormonal Regulations in Plants, Institute of Experimental Botany CAS, Rozvojová 263, CZ-165 02 Prague 6, Czech Republic;

^2^ Department of Chemical Biology and Genetics, Centre of the Region Haná for Biotechnological and Agricultural Research, Faculty of Science, Palacký University, Šlechtitelů 27, CZ-783 71 Olomouc, Czech Republic;

^3^ Centre for Phycology and Biorefinery Research Centre of Competence, Institute of Botany CAS, Dukelská 135, CZ-379 82 Třeboň, Czech Republic;

^4^ Isotope Laboratory, Institute of Experimental Botany CAS, Vídeňská 1083, CZ-142 20 Prague 4, Czech Republic;

^5^ Department of Taxonomy and Biosystematics, Institute of Botany CAS, Zámek 1, CZ-252 43 Průhonice, Czech Republic;

^6^ Laboratory of Growth Regulators, Centre of the Region Haná for Biotechnological and Agricultural Research, Faculty of Science of Palacký University & Institute of Experimental Botany CAS, Šlechtitelů 27, CZ-783 71 Olomouc, Czech Republic

**Running title:** Phytohormone profiles and metabolism in cyanobacteria and algae

*^*^ For correspondence: E-mail* [*vmotyka@ueb.cas.cz*](mailto:vmotyka@ueb.cas.cz)

**ABSTRACT**

- *Background and Aims* Metabolism of cytokinins (CKs) and auxins in vascular plants is relatively well understood, however, data concerning their metabolic pathways in non-vascular plants are still rather rare. With the aim to fill this gap, twenty representatives of taxonomically major lineages of cyanobacteria and algae from Cyanophyceae, Xanthophyceae, Eustigmatophyceae, Porphyridiophyceae, Chlorophyceae, Ulvophyceae, Trebouxiophyceae, Zygnematophyceae and Klebsormidiophyceae were (*1*) analysed for endogenous profiles of CKs and auxins and some of them used for studies of (*2*) metabolic fate of exogenously applied radiolabelled CK, [^3^H]*trans*-zeatin (*trans*Z), and auxin, [^3^H]indole-3-acetic acid (IAA), and (*3*) dynamics in endogenous CK and auxin pools during algal growth and cell division.
- *Methods* Quantification of phytohormone levels was performed by high-performance or ultra-high-performance liquid chromatography-electrospray tandem mass spectrometry (HPLC-MS/MS, UHPLC-MS/MS). The dynamics of exogenously applied [^3^H]*trans*Z and [^3^H]IAA in cell cultures were monitored by HPLC with on-line radioactivity detection.
- *Key Results* The comprehensive screen of selected cyanobacteria and algae for endogenous CKs revealed a predominance of bioactive and phosphate CK forms while *O*- and *N*-glucosides evidently did not contribute greatly to the total CK pool. The abundance of *cis*-zeatin-type CKs and occurrence of CK 2-methylthioderivatives pointed out the tRNA pathway as a substantial source of CKs. The importance of tRNA biosynthetic pathway was proved by establishment of tRNA-bound CKs in the course of *Scenedesmus obliquus* growth. Among auxins, free IAA and its oxidation catabolite 2-oxindole-3-acetic acid represented prevailing endogenous forms. After treatment with [^3^H]IAA, IAA-aspartate and indole-3-acetyl-1-glucosyl ester were detected as major identified metabolites. Moreover, different dynamics of endogenous CKs and auxins profiles during *Scenedesmus obliquus* cultivation period clearly demonstrated diverse roles of both phytohormones in algal growth and cell division.
- *Conclusions* Our data suggest the existence and functioning of a complex network of metabolic pathways and activity control of CKs and auxins in cyanobacteria and algae that apparently differ from vascular plants.

Key words: cytokinin, auxin, cyanobacteria, algae, metabolism, cytokinin oxidase/dehydrogenase, cytokinin 2-methylthioderivatives, *trans*-zeatin, indole-3-acetic acid, tRNA

**INTRODUCTION**

Many aspects of plant growth and development are coordinated by plant hormones. Among them, cytokinins (CKs) represent one of the most important groups playing a key role in cytokinesis and regulation of cell cycle. In addition, CKs affect a number of other physiological processes such as e.g. morphogenesis, apical dominance, leaf senescence, chloroplast development and seed dormancy (Hwang *et al*., 2012; Miller *et al*., 1956). Naturally occurring CKs are *N^6^*-substituted adenine derivatives with isoprenoid or aromatic side chain functioning specifically at minute concentrations (10^-6^ M to 10^-9^ M) in plant tissues (Kieber and Schaller, 2014; Santner *et al*., 2009). While *N^6^*-(Δ^2^-isopentenyl)adenine (iP), *trans*-zeatin (*trans*Z), *cis*-zeatin (*cis*Z), dihydrozeatin (DHZ) and their derivatives are typical representatives of isoprenoid CKs, *N^6^*-benzyladenine (BA) and its hydroxylated forms *ortho*-topolin and *meta*-topolin represent common aromatic CKs. According to their structure and physiological activity, CKs are categorized into (I) bioactive forms including free bases and corresponding nucleosides and their precursors, nucleotides, and (II) non-active or weakly active forms, CK-*O*- and CK-*N*-glucosides (Sakakibara, 2006). In the plant kingdom, a wide spectrum of CK derivatives has been found to occur ubiquitously in vascular plants (Gajdošová *et al*., 2011; Spíchal, 2012) as well as in bryophytes (Záveská Drábková *et al.*, 2015) and fungi (Morrison *et al*., 2015). In contrast to vascular plants, none or only trace amounts of CK *N-*glucosylated forms and mostly rather low levels of CK *O*-glucosides have been reported in cyanobacteria or algae (Hussain *et al*., 2010; Őrdőg *et al.*, 2004; Stirk *et al*., 2003; Stirk *et al*., 2013; Tarakhovskaya *et al*., 2007).

Cyanobacteria as photosynthetic microorganisms exhibit beneficial effects on plant growth through their CK-like activity in processes of atmospheric nitrogen fixation and thus are successfully utilized in agriculture (Abdel-Raouf *et al*., 2012; Stirk *et al*., 1999; Stirk *et al*., 2002). A relatively simple CK metabolism in cyanobacteria was predicted based on a search of CK-related homologous genes involved in CK biosynthesis and degradation pathways. Frébort *et al*. (2011) and Kakimoto (2003) confirmed that isopentenytransferases (IPTs) catalysing the first step in CK biosynthesis in cyanobacteria have a high level of similarity with bacterial tRNA isopentenyltransferases (tRNA-IPTs) and adenylate isopentenyltransferases (AMP/ADP/ATP-IPT). Recently, function of gene encoded adenylate-IPT in the cyanobacterium *Nostoc* sp. PCC 7120 has been reported although it clusters to plant tRNA-IPT (Frébortová *et al*., 2015). Interestingly, a putative CK oxidase/dehydrogenase (CKX) homologous gene sequence involved in CK degradation was discovered in *Nostoc* sp. PCC 7120 (NsCKX1), but the predicted NsCKX1 similarity with the plant CKX proteins was very low (Schmülling *et al*., 2003). Moreover, functional analysis of the recombinant CKX protein named in the study as NoCKX1 revealed no detectable activity for CKs downregulation (Frébortová *et al.*, 2015). In addition, no matching CKX sequences were detected in *Synechocystis* sp. PCC 6803 and *Prochlorococcus marinus* oppositely to some other cyanobacteria species (Frébort *et al*., 2011; Schmülling *et al*., 2003). On the other hand, the regulatory effect of CKs on cyanobacteria metabolism has been reported for *Synechocystis* sp. PCC 6803 strain where BA and *trans*Z enhanced RNA synthesis *in vitro*. A strongly activated RNA transcription in the presence of *trans*Z and CK-binding protein suggested an existence of a potential system of CK signal recognition, which might be transferred to the plant cell in cyanobacteria (Selivankina *et al*., 2006). Although a gene sequence with high similarity to CK membrane receptor CRE1 in *Synechocystis* sp. PCC 6803 was found, more details concerning gene expression and function are still missing (Anantharaman and Aravind, 2001; Selivankina *et al*., 2006).

Algae are highly diverse, non-monophyletic group of photosynthetic eukaryotes occurring in marine, freshwater and land habitats, where sufficient photosynthetic light is available (Lewis and McCourt, 2004). Variable profiles of both isoprenoid and aromatic CKs have been assigned in various algae taxa with some general trends including *cis*Z-types prevalence and low or undetectable contents of DHZ forms and CK conjugates (Stirk *et al*., 2003; Stirk *et al*., 2013; Tarakhovskaya *et al*., 2007). In addition, variation of endogenous CK levels was demonstrated during the cell division cycle of *Chlorella minutissima* in response to light/dark treatment thus suggesting a potential requirement of CKs for algae growth (Stirk *et al*., 2011; Stirk *et al*., 2014). Even though genes coding for CK metabolic pathways have been identified in several algal species, most of them occur sporadically in comparison to vascular plants (Kiseleva *et al*., 2012; Lu *et al*., 2014; Pils and Heyl, 2009). Similarly to cyanobacteria, algal IPTs are rather related to tRNA-IPTs than to adenylate ones thus supporting the origin of CKs from tRNA (Lu and Xu, 2015). Taken together, the full set of proteins participating in CK metabolism has apparently been evolved in particular in green plants (Lu *et al*., 2014; Pils and Heyl, 2009), although as yet unknown mechanisms controlling CK homeostasis probably exist in evolutionary older organisms such as cyanobacteria and algae.

Phytohormone auxin is well known for its key role in regulation of plant growth and development, especially for its impact on cell polarity and cell patterning during embryogenesis and postembryonic development, plant tropic responses, phyllotaxis, floral organs, leaf and vascular tissue formation, root development and *de novo* organogenesis (Benková *et al.*, 2009; Blilou *et al.*, 2005; Cheng *et al.*, 2006; Cheng *et al.*, 2007; Friml *et al.*, 2003; Pernisová *et al*., 2011; Woodward and Bartel, 2005). During several recent years our knowledge about auxin biosynthetic pathways dramatically increased. Tryptophan (Trp) dependent biosynthesis believed to be the main route for IAA synthesis and currently four individual pathways are proposed, each named after the intermediate immediately downstream of Trp – the indole-3-acetaldoxime (IAOx), indole-3-acetamide (IAM), tryptamine (TRA) and indole-3-pyruvic acid (IPyA) (reviewed by Ljung, 2013; Tivendale *et al.*, 2014). As a Trp-independent, indole-3-glycerol phosphate (IGP) pathway has been described in *Arabidopsis* (Normanly *et al.*, 1993; Ouyang *et al.*, 2000; Tivendale *et al.*, 2014; Wang *et al.*, 2015).

Based on physiological activity and chemical structure naturally occuring auxins and its derivatives can be classified to: (I) biologically active forms such as indole-3-acetic acid (IAA), 4-chloroindole-3-acetic acid (4-Cl-IAA) and indole-3-butyric acid (IBA); (II) proposed precursors of IAA biosynthetic pathways such as IAOx, IAM, TRA, IPyA, indole-3-acetonitrile (IAN) and indole-3-acetaldehyde (IAAld); (III) auxin metabolites such as methyl-IAA (MeIAA) with proposed storage role, amino acid conjugates as proposed metabolites of a degradation pathway (indole-3-acetic acid-aspartate, IAA–Asp; indole-3-acetic acid-glutamate, IAA–Glu) or inhibitors of auxin action (indole-3-acetic acid-tryptophane, IAA–Trp). Moreover, some of auxin conjugates can also be hydrolysed back to free IAA *via* activity of amino acid conjugate hydrolases (indole-3-acetic acid-alanine, IAA–Ala; indole-3-acetic acid-leucine, IAA–Leu; indole-3-acetic acid-phenylalanine, IAA–Phe), auxin catabolite 2-oxindole-3-acetic acid (OxIAA) ensuring rapid inactivation of IAA *via* oxidation, and conjugates with sugars such as indole-3-acetyl-1-glucosyl ester (IAA-GE) and oxindole-3-acetic acid-glucosyl ester (oxIAA-GE) (Korasick *et al*., 2013; Ludwig-Müller, 2011).

Endogenous IAA has already been detected in cyanobacteria (Hussain *et al*., 2010; Mazhar *et al.*, 2013; Sergeeva *et al*., 2002) as well as in brown algae (Le Bail *et al*., 2010), red algae (Ashen *et al*., 1999; Yokoya *et al*., 2010) and green algae (Cooke *et al*., 2002; Mazur *et al*., 2001; Stirk *et al*., 2013). Likewise, other auxin precursors and metabolites represented by Trp, anthranilate, IAM, indole-3-ethanol and IAOx were reported in substantial amounts in algae (Stirk *et al*., 2014; Stirk *et al*., 2013; Yokoya *et al*., 2010) while in some cyanobacteria species IBA was found as a predominant metabolite (Hashtroudi *et al*., 2013). Similarly, presence of endogenous phenyl acetic acid (PAA) has been detected in red and green algae (Abe *et al*., 1974; Rocha *et al*., 2011) and the effect of PAA application in comparison with IAA on the levels of metabolically active compounds and growth of green algae *Chlorella vulgaris* has been described by Piotrowska-Niczyporuk and Bajguz (2014). Moreover, Sugawara *et al*. (2015) have recently shown basic characteristics of PAA transport, metabolism and its role in auxin signalling in vascular as well as non-vascular plants.

Positive effects of IAA exogenous application have been reported e.g. for improvement of algal growth rate (Park *et al*., 2013), oil content increase (Jusoh *et al*., 2015; Maor, 2010) and for induction of tolerance to higher salinity and temperatures (Nowak *et al*., 1988; Piotrowska-Niczyporuk and Bajguz, 2014). Additionally, the enhancement of growth parameters and biomass production throughout inoculation of several cyanobacterial strains for their auxin-like activity were observed in wheat (Mazhar *et al*., 2013) or sunflower (Varalakshmi and Malliga, 2012), for instance.

The goal of this study was to characterize CK and auxin metabolism in cyanobacteria and algae as the ancestors of vascular plants. In order to get insight into potential metabolic pathways involved in control of the two phytohormones homeostasis, quantification of endogenous profiles of CKs and auxins as well as determination of their levels following exogenous radiolabelled *trans*Z and IAA application have been performed in distinct cyanobacteria and algae taxa. Last but not least, endogenous CK and auxin pools have been determined during *Scenedesmus obliquus* cultivation period with the aim to demonstrate roles of both phytohormones in the algal growth and cell division.

**MATERIALS AND METHODS**

***Chemicals***

All CKs were supplied by Olchemim, Ltd. (Olomouc, Czech Republic); other chemicals were purchased from Sigma-Aldrich, Inc. (St. Louis, MO, USA). [2-^3^H]*trans*-zeatin ([^3^H]*trans*Z; specific radioactivity 29.7 Ci mmol^-1^), [2-^3^H]*cis*-zeatin ([^3^H]*cis*Z; specific radioactivity 29.7 Ci mmol^-1^) and [2-^3^H]*N^6^*-(Δ^2^-isopentenyl)adenine ([^3^H]iP; specific radioactivity 35.1 Ci mmol^-1^) were supplied by the Isotope Laboratory, Institute of Experimental Botany CAS (Prague, Czech Republic). [5-^3^H]indole-3-acetic acid ([^3^H]IAA), [5-^3^H]2,4-dichlorophenoxy acetic acid ([^3^H]2,4-D) and [4-^3^H]naphthalene-1-acetic acid ([^3^H]NAA) (speciﬁc radioactivity 20.0 Ci mmol^-1^ each) were supplied by the American Radiolabeled Chemicals, Inc. (St. Louis, MO, USA).

***Experimental material***

Twenty representatives of taxonomically major lineages of cyanobacteria and algae belonging to nine classes (Cyanophyceae, Xanthophyceae, Eustigmatophyceae, Porphyridiophyceae, Chlorophyceae, Ulvophyceae, Trebouxiophyceae, Zygnematophyceae and Klebsormidiophyceae) were provided by the Culture Collection of Autotrophic Organisms (CCALA, http://ccala.butbn.cas.cz/index.php). An overview of the selected species used in the study as well as their taxonomic classification is given in **Supplementary** **Table S1**, and their position within a simplified phylogenetic tree is shown in **Fig. 1**.

***Cultivation conditions of cyanobacterial and algal cultures***

In CCALA, cyanobacterial and algal strains have been maintained on agar slant under controlled light and temperature conditions, i.e. light intensity 23 μmol photons m^-2^ s^-1^ of photosynthetic active radiation (PAR), 12 h light/12h dark photoperiod and temperature 12-15°C. In order to become adapted for the cultivation experiments, the cells were transferred into flasks containing 100 ml of 1/2 SŠ medium (Přibyl *et al*., 2015) and pre-cultivated at 80 µmol m^-2^ s^-1^ PAR at room temperature for a few days until appropriate amounts of biomass were reached. Cultures were shaken manually several times a day. Strains for experiments with exogenously applied phytohormones were then transferred into bubble columns of 3.8 cm inner diameter (Kavalierglass, Prague, Czech Republic) at a continuous incident light intensity of 230 µmol m^-2^ s^-1^ PAR at room temperature and bubbled with 2% CO_2_ (v/v) in air. Cultures were collected within the early stationary growth phase (after 10-14 days) and subsequently used for further experiments. Strains for phytohormones profiling were cultivated in the same flasks and under the same cultivation conditions as given above for another 3-4 weeks to reach the early stationary growth phase. Before identification and quantification of CKs and auxins, cyanobacteria and algae suspension cultures were centrifuged for 20 min at 20 000 x *g* and 4°C (Beckman Coulter, Inc., Palo Alto, CA, USA). Subsequently, supernatants were removed by pipetting and pellets were immediately frozen in liquid nitrogen.

***Endogenous cytokinin and auxin profiles in cyanobacteria and algae species***

Endogenous CKs and auxins were extracted from homogenized cyanobacteria and algae samples (0.108-0.251 g fresh weight) according to previously described method (Dobrev and Kamínek, 2002). Determination and quantification of CKs and auxins was performed by a high-performance liquid chromatography (Ultimate 3000, Dionex) coupled to a hybrid triple quadrupole/linear ion trap mass spectrometer (3200 Q TRAP, Applied Biosystems) using a multilevel calibration graph with [^2^H]-labelled internal standards as described previously (Djilianov *et al*., 2013; Dobrev *et al*., 2005; Žižková *et al*., 2015). Detection of 2-methylthioderivatives of iP, *trans*Z and their ribosides was set up in a selected reaction-monitoring mode.

***Metabolism of exogenously applied [^3^H]*trans*Z, [^3^H]IAA, [^3^H]NAA and [^3^H]2,4-D in cyanobacteria and algae cells***

Metabolic profiles of exogenously applied radiolabelled *trans*Z ([^3^H]*trans*Z, 1.5 x 10^6^ dpm µL^-1^) added into the culture media at final concentration 1 µM or 20 nM, respectively, were determined in one cyanobacterium (*Chroococcus minutus*; CCALA 55) and three algae (*Chlorococcum elbense*, CCALA 282; *Klebsormidium flaccidum*, CCALA 786; *Scenedesmus obliquus*, CCALA 454) strains cultivated in liquid media as described previously and regenerated overnight in cultivation room (16 h light/ 8 h dark photoperiod, 20°C) under continuous shaking (120 rpm, orbital diameter 20 mm). Cells and media (200 mg fresh weight and 10 ml per sample) were collected separately by filtering through Whatman GF/C glass fiber filtres (5 cm diameter) at time points 0 h, 0.5 h, 1 h, 2 h, 4 h and 24 h.

Similarly, radiolabelled [^3^H]IAA, [^3^H]NAA or [^3^H]2,4-D were added into the culture media of four algae strains (*Chlorococcum ellipsoideum*, CCALA 283; *Stigeoclonium helveticum*, CCALA 868; *Scenedesmus obliquus*, CCALA 454 and *Microthamnion kuetzingianum*, CCALA 368) at final concentration 20 nM. Cells and media were collected separately by filtering through Whatman GF/C glass fiber filtres (5 cm diameter) at time points 0 h, 1 h, 2 h and 6 h.

Radiolabelled metabolites of *trans*Z and auxins were separately analysed by HPLC coupled to on-line radioactivity detector under the same analytical conditions, with exception of the different gradients. HPLC column Luna C18 (2), 150 × 4.6 mm, 3 µm (Phenomenex, Torrance, CA, USA) was used, mobile phase A was 40 mM CH_3_COONH_4_ (pH 4) and mobile phase B was CH_3_CN/CH_3_OH, 1/1 (v/v). Flow rate was 0.6 ml min^-1^. The linear gradient programme for *trans*Z metabolites was: 10–40 % B for 12 min, 40–100 % B for 1 min, 100 % B for 2 min, 100–10 % B for 1 min. The linear gradient programme for auxin metabolites was: 30–50 % B for 10 min, 50–100 % B for 1 min, 100 % B for 2 min, 100–30 % B for 1 min. The column eluate has been monitored by a Ramona 2000 on-line radioactivity detector (Raytest GmbH, Straubenhardt, Germany) after on-line mixing with three volumes (1.8 ml min^-1^) of liquid scintillation cocktail (Flo-Scint III, Perkin Elmer Life and Analytical Sciences, Shelton, CT, USA). The radioactive metabolites were identified on the basis of comparison of their retention times with authentic standards. Results of auxin metabolic profiles are presented as total integrated area of chromatogram plots normalized to the equalization of total accumulated radiolabel.

***Cytokinin oxidase/dehydrogenase in vitro assay***

The enzyme preparations were extracted and partially purified using the method described by Motyka *et al*. (2003). The CKX activity was determined by *in vitro* assays based on the conversion of [2-^3^H] labelled CKs ([^3^H]*trans*Z, [^3^H]*cis*Z and [^3^H]iP**)** to [^3^H]-adenine. Separation of the substrate from the product of the enzyme reaction was achieved by HPLC as described by Gaudinová *et al.* (2005). The CKX activity was determined in duplicates in two independent experiments.

***Growth of* Scenedesmus obliquus**

Pre-cultivated culture of microalgae *Scenedesmus obliquus* was diluted with 1/2 SŠ fresh medium (Přibyl *et al.*, 2015) to obtain the cell density of 1.0-1.5 x 10^6^ cells ml^−1^ (around 0.15-0.20 g l^-1^ dry weight). The cell density was determined using a Bürker counting chamber (Hecht-Assistent, Sondheim, Germany); at least 400 cells were counted. The culture was cultivated in bubble columns of 3.8 cm inner diameter (Kavalierglass, Prague, Czech Republic) at a continuous incident light intensity of 500 µmol m^-2^ s^-1^ PAR at 30 ± 0.5°C and bubbled with 2 % CO_2_ (v/v) in air. The dilution procedure was repeated twice each 24 h and resulting inoculum was used for batch-cultivation experiments in the initial volume of 150 ml under the same conditions as described above for 14 days. During cultivation, samples for analyses were taken regularly following the replenishment of water evaporated from the bubble columns. Growth was determined gravimetrically based on increased cell dry weight as follows: culture samples (1-5 ml) were centrifuged (10 000 x *g*, 8 min) in pre-weighed microtubes and the sediment was dried at 105°C. The cell density was quantified using a Bürker counting chamber (Hecht-Assistent, Sondheim, Germany); at least 600 cells were counted for each sample. Samples for CK analyses were collected in the same intervals; biomass was separated from the growth medium by centrifugation (10 000 x *g*, 8 min) and both biomass and medium were immediately frozen in liquid nitrogen.

***Endogenous cytokinin profiles during* Scenedesmus obliquus *growth***

The frozen pellets (as described above) used for determination of CKs were freeze dried (Scanvac CoolSafe 110-4, Fisher Scientific) in vacuum (Savant^TM^ SPD 121P SpeedVac^TM^ Concentrator, Thermo Scientific^TM^) for 7 h. For analysis of free CKs, *Scenedesmus obliquus* samples (5 mg dry weight of each) were homogenized under liquid nitrogen, extracted in modified Bieleski buffer (methanol/ water/formic acid, 15/4/1, v/v/v) containing 0.2 pmol of [^2^H]- or [^13^C]-labelled CK free-bases/ribosides/*N*-glucosides and 0.5 pmol of [^2^H]-labelled CK-*O*-glucosides/nucleotides (Novák *et al.*, 2003; Novák *et al.*, 2008), and then purified using two solid phase extraction columns, the C18 octadecylsilica-based column and the MCX column (Dobrev and Kamínek, 2002). Analytes were eluted by two-step elution using a 0.35 M NH_4_OH aqueous solution and 0.35 M NH_4_OH in 60% (v/v) MeOH. CK levels were determined by ultra-high-performance liquid chromatography-electrospray tandem mass spectrometry (UHPLC-MS/MS) with stable isotope-labelled internal standards as a reference (Svačinová *et al.*, 2012).

Extraction and purification of tRNA was performed according to a protocol described by Maass and Klämbt (1981) including modifications described by Stirk *et al*. (2011). Aliquots of extracted total tRNA were hydrolysed with 2 M KOH overnight and dephosphorylated by alkaline phosphatase. Addition of internal standards (0.2 pmol of each [^2^H]-labelled CK ribosides), samples purification on MCX column and tRNA-bound CKs quantification was performed by UHPLC-MS/MS as described above. 2-methylthio-derivatives of tRNA-bound isoprenoid CKs were analyzed by HPLC-MS/MS system as described previously (Tarkowski *et al*., 2010). The extraction and purification of *Scenedesmus obliquus* samples were carried out in two technical replicates for each biological replicate.

***Presentation of the results***

Each evaluation was carried out in duplicates in two or three independent experiments. The results are expressed as mean values including standard deviation (SD) of the means in the figures and/or tables.

**RESULTS**

***Selection of cyanobacteria and algae species***

In order to extend a current knowledge concerning CK and auxin metabolism in non-vascular organisms, the search for suitable cyanobacteria and algae candidates was performed with respect to their distinct evolutionary history, taxonomic position and habitat requirements. The complete list and abbreviations of all representatives analysed for CK and auxin profiles including cyanobacteria and the major lineages of algae belonging to brown algae (Ochrophyta), red algae (Rhodophyta) and green algae (Chlorophyta and Streptophyta) is shown in **Supplementary** **Table S1**.

The position of selected taxa within a simplified phylogenetic tree based on different data sources from the whole chloroplast genome and nuclear rDNA (Riisberg *et al.*, 2009; Ruhfel *et al.*, 2014) is demonstrated in **Fig. 1**. In addition to Prokaryota represented by three cyanobacteria species (*Chroococcus minutus*, *Phormidium animale* and *Nostoc microscopicum*), three ochrophytes (*Tribonema aequale*, *Bumilleriopsis filiformis* and *Vischeria helvetica*), two red algae (*Porphyridium purpureum* and *Rhodella violacea*) and two major lineages of green algae referred to chlorophyte and charophyte/streptophyte clade (see **Supplementary Table S1**) were involved within the analysed set of eukaryotic organisms. Nine Chlorophyta species belonging to three major groups (Chlorophyceae, Ulvophyceae and Trebouxiophyceae) and two Streptophyta species (*Actinotaenium curtum*, *Klebsormidium flaccidum*) as representatives of evolutionary more advanced organisms closely related to vascular plants were selected within the green algae for analyses of CK and auxin spectra (**Fig. 1**; **Supplementary Table S1**). Another Chlorophyta species, *Chlorococcum elbense*, was chosen for metabolic studies only. To summarize, the set of analysed samples has been representative enough to enable a very comprehensive survey of regulation of CK and auxin metabolism in photoautotrophic microorganisms of different phylogenetic origin.

***Cytokinin profiles in cyanobacteria and algae substantially differ from vascular plants***

In analogy to vascular plants, a wide spectrum of isoprenoid CKs was detected in both cyanobacteria and algae. Total CK levels in different species varied from picomols per g FW (e.g. *Porphyridium purpureum*, 2.53 pmol g^-1^ FW) to hundreds of picomols (e.g. *Phormidium animale*, 178.55 pmol g^-1^ FW) (**Fig. 2**; **Supplemetary Table S2**). Bioactive CKs (free bases and ribosides) and CK phosphates were found as the prevalent CK forms being present in concentrations from 1.01 pmol g^-1^ FW (*Porphyridium purpureum*) to 100.19 pmol g^-1^ FW (*Chlamydomonas segnis*) and from 0.56 pmol g^-1^ FW (*Chlamydomonas segnis*) to 65.36 pmol g^-1^ FW (*Phormidium animale*), respectively. On the other hand, none or only trace amounts of CK-*N*-glucosides were detected throughout the whole set of analysed species. Similarly, CK-*O*-glucosides occurred only in minute concentrations or were absent in the tested samples (**Fig. 2A**; **Supplementary Table S2**). In general, the iP-, *cis*Z- and *trans*Z-type CKs predominated in all analysed cyanobacteria and algae, while DHZ-types contributed only insignificantly (with concentrations ranging from 0.35 to 3.95 pmol g^-1^ FW) to the total CK pool. Interestingly, whereas monophosphate forms of iP, *trans*Z and DHZ were relatively abundant in most of the species, *cis*Z was found only in biologically active free-base and riboside forms in all of the analysed taxa. In thirteen (out of nineteen) analysed taxa, the levels of *cis*Z and its riboside exceeded those of corresponding *trans*Z counterparts, in most of them being more than at least threefold higher (**Fig. 2B**; **Supplemetary Table S2**). Interestingly, 2-methylthio-*N^6^*-(Δ^2^-isopentenyl)adenosine (2MeSiPR) was detected in moderate or high concentrations in almost all of the analysed samples representing a predominant metabolite in some Cyanobacteria (*Chroococcus minutus, Phormidium animale*) and Chlorophyta (*Chlorococcum ellipsoideum, Pseudendoclonium basiliense*, *Scenedesmus obliquus*, *Microthamnion kuetzingianum)* species (**Fig. 2B**; **Supplementary Table S2**). In summary, the profiles of endogenous CKs in selected cyanobacteria and algae revealed a predominance of biologically active and phosphate CK forms and a relative irrelevance of CK-*O*- and *N*-glucosides. Moreover, it is demonstrated that *cis*Z-type CKs and 2MeSiPR substantially contribute to the overall CK pool, indicating an existence of diverse metabolic pathways in cyanobacteria and algae compared to vascular plants.

***Spectra of auxin metabolites in cyanobacteria and algae are rather narrow***

In all of the selected cyanobacteria and algae, the screen of endogenous CKs was supplemented by analysis of endogenous indole auxin levels. In the whole spectrum of analysed species and biological samples, the auxins IAA, its primary catabolite OxIAA and an amino acid conjugate, IAA-Asp, were detected. The total auxin concentrations ranged from 10.93 to 290.69 pmol g^-1^ FW, with the minimal as well as the maximal endogenous levels in Chlorophyta species *Pseudeclonium basiliense* and *Stigeoclonium helveticum*, respectively (**Fig. 2C**; **Supplementary Table S3**). The main auxins were represented by free IAA (occurring in concentrations from 3.26 to 287.57 pmol g^-1^ FW; *Porphyridium purpureum* and *Stigeoclonium helveticum,* respectively) and OxIAA (ranging from 1.78 to 43.54 pmol g^-1^ FW; *Bumilleriopsis filiformis* and *Protosiphon* botryoides, respectively) whereas concentrations of IAA-Asp were close to the detection limit in all of the tested species (**Fig. 2C**; **Supplementary Table S3**). Taken together, endogenous free IAA and OxIAA evidently represent predominant indole auxin forms in the selected cyanobacteria and algae samples.

*Cyanobacteria and algae metabolize exogenously applied [^3^H]*trans*Z*

In order to study regulation of CK levels in non-vascular plants and to compare it with vascular plants, radiolabelled *trans*Z was exogenously applied into the cultures of cyanobacteria (*Chroococus minutus*) and selected algae (*Chlorococcum elbense, Klebsormidium flaccidum and Scenedesmus obliquus*). Following [^3^H]*trans*Z treatment, some relatively rapid metabolic changes in cultured cells were observed as early as after 1 h incubation (Fig. 3A-D). After 4 h incubation, the degradation products adenine (Ade; RT=4.15 min) and adenosine (Ado; RT=7.12 min) together with other substances such as AMP/ADP/ATP (RT=4.0 min), DHZ (RT=16.5 min) and its riboside (RT=18.5 min) were found based on their retention times on HPLC. After 24 h, almost complete conversion of [^3^H]*trans*Z was apparent in *Chroococcus minutus*, *Scenedesmus obliquus* and *Chlorococcum elbense* (Fig. 3A-C), which was in contrast to *Klebsormidium flaciduum* where a relatively high amount of [^3^H]*trans*Z still persisted (about one fourth of the initial; Fig. 3D). Interestingly, relatively high concentrations of fastly formed unknown metabolites were detected after [^3^H]*trans*Z treatment in all of the four analysed species (Fig. 3A-D).

In addition, metabolism of [^3^H]*trans*Z was also followed in the cultivation medium of *Chroococcus minutus* and *Klebsormidium flaciduum.* After incubation (4 h and 24 h), [^3^H]*trans*Z was largely converted in the *Klebsormidium flaciduum* medium with subsequent accumulation of DHZ and several unidentified metabolites (Supplementary Data Fig. S1). Similarly, a decline of [^3^H]*trans*Z 24 h after its supply was recorded and some unidentified metabolites were detected in the medium of *Chlorococcus minutus* (data not shown). Altogether, our data strongly suggest a potential intra- as well as extracellular regulation of the CK status in both cyanobacteria and algae cells.

The intense *in vivo* formation of adenine and adenosine as products of [^3^H]*trans*Z metabolism in selected cultures rises a question of a potential involvement of CKX activity in degradation of CKs in cyanobacteria and algae. To investigate this subject, degradation of [^3^H]*trans*Z, [^3^H]*cis*Z and [^3^H]iP by the CKX activity isolated from crude protein preparations of eight species including Cyanobacteria, Rhodophyta, Ochrophyta and Chlorophyta was determined. The *in vitro* enzymatic assays performed at two pH values, pH 7.0 and pH 8.5 (Supplementary Data Fig. S2, S3 for [3H]iP; data not shown for ^3^[H]*trans*Z, [^3^H]*cis*Z) revealed no CKX activity for any of the tested samples. Interestingly, an unknown metabolite (RT=2.5 min) was formed *in vitro* as a product of radiolabelled CK substrates in all of analysed samples (Supplementary Data Fig. S2, S3; data not shown). To summarize our finding, in spite of the intense *in vivo* conversion of [^3^H]*trans*Z to adenine and/or adenosine, the assumed CKX activity was not detected.

***Exogenously applied [^3^H]IAA is gradually metabolized in algae cell cultures***

In order to characterize more precisely the auxin metabolism in non-vascular plants, radiolabelled [^3^H]IAA was exogenously applied into the cultures of four Chlorophyta species including *Stigeoclonium helveticum,* *Chlorella vulgaris*, *Microthamnion kuetzingianum* and *Scenedesmus obliquus.* In all of the analysed green algae, exogenously applied [^3^H]IAA was gradually metabolized in both cells (**Fig. 4**) and media (**Fig. 5)**. During 6 h of [^3^H]IAA treatments, formation of IAA-Asp (RT=6.6 min), IAA-GE (RT=8.12 min) and eight other unidentified metabolites with a major product in retention time 14.23 min have been detected. To compare auxin metabolic profiles in algae cells after addition of synthetic radiolabelled auxin compounds, [^3^H]NAA and [^3^H]2,4-D have been applied. Surprisingly, in comparison to numerous conversions of exogenously applied IAA, no significant effect on substrate metabolization in selected algae cells and media was observed (data not shown), probably due to missing metabolic pathway(s) for such unnaturally occurring substrates or an inability to transport them inside the cells.

***Cytokinin and auxin metabolite profiles are differently affected during* Scenedesmus obliquus *growth***

With the aim to decipher regulation of CK and auxin homeostasis in non-vascular organisms, *Scenedesmus obliquus* was used as a representative culture of green algae from Chlorophyte clade with respect to its relatively facile cultivation. The cellular growth and concentrations of CKs (both free and tRNA-bound) and auxins were recorded in the course of *S. obliquus* culturing (**Fig. 6**).

After a short lag phase, a rapid increase in cell number was detected, reaching the stationary growth phase already after 2-3 days of cultivation. The biomass dry weight showed a similar pattern, however, with a substantial delay in entering the stationary growth phase (**Fig. 6A**). Within the exponential and linear phases of cellular growth (0-4 d), the total free CK concentration was at least three times as high as in the stationary phase (7-14 d). Among CK groups, CK phosphates represented the predominant CK forms (67-81% of total) whereas the rest of CK pool was particularly comprised of bioactive free bases and ribosides. CK-*O*-glucosides were found at very low concentrations close to the detection limit (ranging from 0.09-0.58 pmol g^-1^ FW) and CK-*N*-glucosides were not detected at all (**Fig. 6B**). Remarkably, the highest levels of CK phosphates and bioactive CKs were reached up at day 2 (168.48 ± 12.56 pmol g^-1^ FW and 50.50 ± 3.06 pmol g^-1^ FW, respectively) while at least 9-fold decreased contents of both CK groups (12.38 ± 1.90 pmol g^-1^ FW and 5.29 ± 0.26 pmol g^-1^ FW, respectively) have been observed in the stationary phase of *Scenedesmus obliquus* growth (**Fig. 6B).** The CK profiling also revealed that endogenous levels of *cis*Z and its metabolites considerably exceeded concentrations of other CK-types during *S. obliquus* growth and that iP-types represented the second most abundant CK forms (**Fig. 6C**).

To characterize contribution of tRNA biosynthetic pathway on CK pool in the course of *S. obliquus* growth, tRNA-bound CKs were further assessed. The profile of individual CK ribosides derived from tRNA revealed dominant proportion of *cis*Z-9-riboside (*cis*ZR) and *N^6^*-(Δ^2^-isopentenyl)adenosine (iPR) during the first 7 days of *S. obliquus* cultivation (**Fig. 6D**). The highest concentration of *cis*ZR (52.45 ± 2.42 pmol g^-1^ DW) was reached at day 1 of the growth exponential phase while its lowest level was found at the beginning of the stationary phase (day 9; 2.11 ± 0.56 pmol g^-1^ DW). Interestingly, very low concentrations of *trans*Z-9-riboside (*trans*ZR) and DHZ-9-riboside (DHZR) varying from 0.07 ± 0.03 to 0.75 ± 0.10 pmol g^-1^ DW were detected during the whole *S. obliquus* growth cycle (**Fig. 6D).** Consistently, a prevailance of *cis*ZR and iPR was found out also when concentrations of tRNA-bound CKs were recalculated to milligrams of isolated tRNA instead of to DW (**Supplementary Data** **Table S4**). In addition, determination of tRNA-bound 2-methylthioderivatives revealed a presence of 2-methylthio-*cis*Z-9-riboside (2MeS*cis*ZR) and 2MeSiPR during the whole *Scenedesmus obliquus* growth cycle (**Fig. 6E**). Both 2MeS*cis*ZR and 2MeSiPR were detected at the highest concentrations after day 1 of algal cell growth cycle (reaching 26.10 ± 1.84 pmol g^-1^ DW and 16.02 ± 0.55 pmol g^-1^ DW, respectively) followed by continuous decline until day 9 and subsequently by obvious increase at the end of stationary phase (**Fig. 6E**). When related to pmol mg^-1^ tRNA, the contents of 2MeS*cis*ZR and 2MeSiPR have indicated variations especially during the exponential phase of growth (**Supplementary Data** **Table S4**).

To get insight into the complexity of phytohomorne regulation during 14 days of *S. obliquus* cells growth, auxin metabolite profile was also determined (**Supplementary Data** **Table S5**). From the experimental setup, the content of IAA gradually increased reaching its maximum after 13 days of cultivation (84.9 ± 0.96 pmol g^-1^ FW) and then decreased rapidly and noticeably to ca. one half (14 d; 41.4 ± 11.65 pmol g^-1^ FW) (**Fig. 6F**). Concentrations of auxin precursors IAM and IAN were considerably lower than those of free IAA exhibiting moderate variation of their levels during *S. obliquus* growth. Auxin catabolite OxIAA reached its maximum similarly as IAA (at 13 d) whereas the endogenous conjugated forms, IAA-Asp and OxIAA-GE, have been detected at very low concentrations within the exponential and linear growth phases (**Supplementary Data** **Table S5**). Surprisingly, the main auxin metabolite was PAA with about one order of magnitude higher amount than IAA, however, without showing any significant dynamic changes during cultivation period (**Fig. 6F**).

Taken together, our data demonstrate a dynamic regulation of CK and auxin homeostasis during *S. obliquus* cell growth cycle. Enhanced concentration of CKs in cells during the phase of their intensive growth is especially due to an enormous amount of CK phosphates, especially *cis*ZRMP and iPRMP. Substantial amounts of *cis*ZR, iPR and their 2-methylthioderivatives seem to be delivered mainly through tRNA biosynthetic pathway (**Fig. 6**). In contrast, concentrations of auxins (mainly of biologically active IAA) gradually increase from exponential to linear growth phase. In summary, distinct proportions between CK and auxin profiles during *S. obliquus* growth may point out their different functioning and physiological consequences in algal cells.

**DISCUSSION**

It has been postulated that physiological and structural changes in metabolism of vascular plants were progressively developed with the transition from an aqueous to a gaseous environment (Kenrick and Crane, 1997). Thus the components of CK and auxin metabolic pathways identified in vascular plants probably arose from pre-existing elements of bacteria primary metabolism *via* endosymbiosis and horizontal gene transfer (Spíchal, 2012; Yue *et al*. 2014). However, in contrast to relatively well-characterized regulation of CK and auxin homeostasis *via* different metabolic pathways in vascular plants (e.g. Kieber and Schaller, 2014; Korasick *et al*., 2013; Normanly, 2010), data concerning regulatory mechanisms of both phytohormone levels in evolutionary older non-vascular organisms are rather limited. In order to attempt to fill this gap, selected cyanobacteria and algae species belonging to divergent evolutionary lineages (**Fig. 1**; **Supplementary Data** **Table S1**) were screened and analysed for CK and auxin profiles and metabolic pathways involved in their homeostasis control.

For all cyanobacteria and algae species used in this study, some common traits regarding the profiles of isoprenoid CK derivatives were found. In general, bioactive CKs and CK phosphates represented the most prevailing CK forms in contrast to CK-*O*- and *N*-glucosides occurring only in moderate or hardly detectable concentrations (**Fig. 2A**; **Supplementary Data Table S2**). Similar CK spectra as found here have been reported previously by Stirk *et al*. (2013) and Yokoya *et al*. (2010) but there are also few exceptions demonstrating higher proportion of CK-*O*-glucosides in some microalgal and macroalgal strains than shown in our study (Lu *et al.*, 2014; Ördög *et al*., 2004; Stirk *et al.*, 2003). Among the CK derivatives detected, 2MeSiPR was present in most of tested species (**Supplementary Data** **Table S2**) suggesting its tRNA origin as reported by e.g. Prinsen *et al*. (1997). Additionaly, the enzyme catalysing methiolation of CK derivatives was discovered in chloroplast of light-grown photosynthetic protozoan *Euglena gracilis* (Swaminathan and Bock, 1977). Thus it can be assumed that tRNA degradation is an essential source of CKs representing a predominant biosynthetic pathway of CKs in evolutionary older non-vascular organisms such as cyanobacteria and algae.

Among the bioactive CKs, free-bases of iP, *cis*Z and in some species also *trans*Z were found as major CK forms. Their ribosides were detected mostly in lower amounts, and *trans*ZR was completely missing in cyanobacteria and just sporadically detected in algae (**Supplementary Data** **Table S2**). Regarding zeatins, *cis* forms were more common than *trans* forms; the contents of *cis*Z and its riboside exceeded those of *trans*Z counterparts in thirteen (out of nineteen) cyanobacterial and algal samples. This prevalence of *cis*Z over *trans*Z types corresponds well with findings reported by other authors (Ördög *et al*., 2004; Stirk *et al*., 2002; Stirk *et al*., 2003 and Stirk *et al*., 2013). Based on these data, we suggest that function of CK-*N*-glucosides (i.e. deactivation or reduction of biological activity) in cyanobacteria and algae may be, at least partially, substituted by *cis*Z types, that represent prevailing and generally less active forms compared to *trans*Z types (Gajdošová *et al*., 2011).

As expected, the CK status in cyanobacteria and algae was not steady and could be dramatically affected by internal as well as external factors. In our study, substantially lower concentrations of isoprenoid CKs than those published by Stirk *et al*. (2013) were found in green algae *Chlorococcum ellipsoideum*, *Protosiphon botryoides* and *Klebsormidium flaccidum*. The differences could be mainly due to distinct growth phases of the analysed species; while the measurements of our samples were performed in the early stationary growth phase, the analyses by Stirk’s group were done in the exponential phase of growth. Additionally, environmental factors might affect the CK profiles in cyanobacteria and algae as well. For instance, the CK spectra were found to be dependent on water temperature in Chlorophyta seaweed *Ulva* sp. (Stirk *et al*., 2003) as well as the effect of light during cell cycle of *Chlorella minutissima* (Stirk *et al*., 2011) and nitrogen depletion in *Nannochloropsis oceanica* (Lu *et al*., 2014) on CK profiles were reported.

The present study also showed a wide concentration range of auxins (free IAA, OxIAA and IAA-Asp) in selected cyanobacterial and algal species (**Supplementary Data** **Table S3**). This is in consistence with previously published results demonstrating a wide variation in concentrations of IAA and other auxin metabolites such as IAA-Glu, IAA-Leu and IAM in cyanobacteria and algae (Hussain *et al*., 2010; Tarakhovskaya *et al*., 2007; Yokoya *et al*., 2010; Yokoya and Yoneshigue-Valentin, 2011). Auxin derivatives detected in our study, OxIAA and IAA-Asp, represent metabolic products of two major catabolic pathways of IAA in plants. The first is an IAA oxidation into the primary catabolite OxIAA (Kai *et al*., 2007a), the second is an amino acid conjugation of IAA with aspartate leading to formation of IAA-Asp (Ljung *et al*., 2002, Ludwig-Müller 2011). Our data revealed that free IAA and OxIAA were the main auxins while IAA-Asp occurred only in concentrations close to the detection limit if present at all (**Supplementary Data** **Table S3**) indicating a higher relevance of oxidative than conjugative pathway in IAA catabolism in cyanobacteria and algae.

Fast regulation of bioactive CK and auxin pools after [^3^H]*trans*Z and [^3^H]IAA respective treatments strongly suggests that cyanobacteria and algae possess effective mechanism(s) controlling CK and auxin homeostasis in cells. Using exogenously applied [^3^H]*trans*Z, a swift conversion within 1 h incubation, especially in *Chroococcus minutus* and *Chlorococcum elbense*, was observed (**Fig. 3A; Fig. 3C**). In spite of an extensive formation of radiolabelled adenine and/or adenosine following [^3^H]*trans*Z treatment, no CKX activity has been detected *in vitro* for any of the species analysed (**Supplementary Data** **Fig. S2, S3**). This is in accordance with recently published data demonstrating only a very sporadic determination of CKX homologous sequences in algae (Lu *et al*., 2014) and no detectable enzymatic activity of NoCKX1 in cyanobacteria *Nostoc* sp. PCC 7120 (Frébortová *et al*., 2015). Notably, the occurrence of [^3^H]*trans*Z metabolite peaks with retention times corresponding to DHZ and DHZR (**Fig. 3**) suggests a potential involvement of zeatin reductase activity detected so far only rather scarcely in some vascular plant species (Martin *et al*., 1989; Gaudinová *et al*., 2005).

It was previously found that feeding of *Arabidopsis* seedlings with IAA led to increased accumulation of IAA-Asp, IAA-Glu, IAA-GE, OxIAA and OxIAA-GE (Kai *et al*., 2007b), most likely due to the activation of multiple metabolic pathways in response to the exogenous IAA supply. Consistently with these results, our findings in selected algal species (*Scenedesmus obliquus*, *Chloroccocum elbense*, *Stigeoclonium helveticum* and *Microthamnion kuetzingianum)* revealed a gradual metabolization of exogenously applied [^3^H]IAA to IAA-Asp and IAA-GE as two major products. Detection of eight more unidentified metabolites in both cells and media may indicate an existence of other probably unknown IAA metabolic pathway(s) in green algae (**Fig. 4; Fig. 5**). Surprisingly, exogenous application of [^3^H]NAA or [^3^H]2,4-D did not cause any significant effect on substrate metabolization (data not shown) although both of these synthetic auxins were shown to stimulate cell growth and to increase cell fresh and dry weight in *Chlorella pyrenoidosa* cultures (Czerpak *et al*., 1994). It can just be speculated that a missing metabolization of [^3^H]NAA or [^3^H]2,4-D is due to non-functional transport mechanisms or absent metabolic pathways for rapid inactivation of these unnatural auxins.

The apparent correlation between *Scenedesmus obliquus* growth and both CKs and auxins levels as demonstrated in **Fig. 6** suggests an indispensable role of the two phytohormones in algal cell division. Correspondingly to the data by Stirk *et al*. (2014), *cis*Z- and iP-types mainly contributed to the total CK pool. Enhanced content of CK phosphates, particularly *cis*ZRMP and iPRMP, during the exponential and linear growth phase (0-3 days) indicated higher CK biosynthetic rates of algal cells in comparison to the stationary phase (**Fig. 6B**). Our data are fully compatible with the assumed roles of *cis*Z and iP nucleotides as immediate products in CK biosynthesis in contrast to *trans*Z and DHZ nucleotides formed particularly by the side chain modification (Hwang and Sakakibara, 2006; Takei *et al*., 2004; Kieber and Schaller, 2014). Screening of tRNA-bound CKs during *S. obliquus* growth also revealed a prevalence of *cis*ZR and iPR contrary to *trans*ZR a DHZR (**Fig. 6E**). iPR and *cis*ZR have also been reported previously as predominant tRNA-bound as well as free CK forms in several cyanobacteria and microalgae species by Šimura *et al*. (2014). The significance of *cis*Z-type CKs in *S. obliquus* growth cycle demonstrated in our observations and their potential origin from tRNA pathway suggested by recent phylogenetic studies, in which higher similarity of IPTs from cyanobacteria and microalgae related to tRNA-IPTs was reported (Frébortová *et al*., 2015; Lu and Xu, 2015), are thus a likelihood. The prevalence of tRNA-bound *cis*ZR and iPR indicating an important involvement of tRNA-dependent CK biosynthetic pathway was described as well in the moss *Physcomitrella patens* (Yevdakova *et al*., 2008). However, a comparison between total concentrations of free CK forms and tRNA-bound CKs during *S. obliquus* growth pointed out a more pronounced production of CKs through *de novo* biosynthesis (**Fig. 6C and 6D**). Likewise, free *cis*Z- and iP-type CK forms were found in considerably higher concentrations than tRNA-bound CKs in vascular plants such as oat, lucerne and maize during their germination and early seedling establishement (Stirk *et al*., 2012).

A subsequent metabolization of CK nucleotides in *S. obliquus* culture is not clear because there was no increase in production of CK metabolites in the cells and the medium after stopping the cell divisions (**Fig. 6B** and data not shown). Moreover, the genes involved in CK conjugation (UGT) and degradation (CKX) have not been functionally characterized in algae yet (Frébortová *et al*., 2015; Lu *et al*., 2014). Thus it can be hypothesized that CK homeostasis during the algal growth is controlled basically by modulation of the pool of CK ribotides functioning at least partially as storage metabolites, however, a more detailed characterization of this type of regulation needs to be further investigated.

In contrast to CK profile, a gradual enhancement of IAA levels during subcultivation period of *S. obliquus* correlated with an increase of cell division during exponential growth phase (2-8 day). After that IAA concentrations reached their maxima at the stationary phase (13 days) and then they declined (**Fig. 6F**). These findings are in consistence with Mähonen *et al*. (2014) who showed that higher auxin levels inhibit cell division and expansion, but not cell differentiation in Arabidopsis. The presence of IAM, a precursor of auxin biosynthesis (reviewed by Woodward and Bartel, 2005; Korasick *et al*., 2013), indicated an involvement of Trp dependent pathway(s) in *S. obliquus*. Detection of OxIAA in *S. obliquus* in our expertiment pointed to the importance of this auxin metabolic product (Östin *et al*., 1998) in regulating bioactive IAA levels in non-vascular organisms in addition to the vascular plants. Although biological activity of PAA is generally lower comparing to IAA (Sugawara *et al*., 2015), the endogenous levels of PAA exceeded considerably those of IAA in *S. obliquus* cells, which corresponds to the data reported for numerous vascular plants (e.g. *Avena sativa* coleoptiles - Wightman and Lighty, 1982; *Pisum sativum* roots - Schneider *et al.*, 1985). Moreover, both PAA and IAA seem to function similarly by regulating auxin-responsive genes through the TIR1/AFB pathway as demonstrated for Arabidopsis (Löbler and Klämbt, 1985), although the direct interaction of PAA with TIR1/AFB and Aux/IAA proteins has yet to be investigated both in vascular (Shimizu-Mitao and Kakimoto, 2014) as well as in non-vascular plants. A relatively steady PAA contents without any significant dynamic changes during *S. obliquus* growth cycle, however, raises a question of its spatiotemporal regulation in algae. To conclude, our results clearly demonstrate a diverse involvement of CKs and auxins during the algae cell growth (**Fig. 6**) indicating specificities of their functioning in analogy to processes known for vascular plants (Coenen and Lomax, 1997).

***Conclusions***

In summary, we present here an insight into the control of CK and auxin homeostasis in evolutionary older non-vascular organisms such as cyanobacteria and algae. The comprehensive screen of selected representatives for endogenous CK and auxin profiles reveals a prevalence of CK phosphates and *cis*Z-type CKs in the total CK pool while in the auxinome free IAA and its primary catabolite OxIAA predominate. For both CKs and auxins, their conjugated forms were not found or were detected only in very low concentrations in cyanobacteria and algae. In contrast to vascular plants, CK down-regulation by the CKX activity was not observed in any of the tested species. Our data also demonstrate an occurrence and significance of CK methylthioderivatives, which points out the importance of tRNA pathway as a substantial source of CKs in cyanobacteria and algae. In addition, we show here a metabolic fate of exogenously applied [^3^H]*trans*Z and [^3^H]IAA in the selected taxa as well as changes in endogenous CK and auxin pools in the course of *Scenedesmus obliquus* cultivation period, during which high concentrations of non-indole PAA exceeding those of indole auxins were detected. Our results suggest the existence and operation of a complex network of metabolic pathways and regulation of activities of CKs and auxins in cyanobacteria and algae apparently differing from vascular plants and reveal a whole range of not yet answered questions regarding control of both phytohormone homeostasis in non-vascular plants.

**FIGURE LEGENDS**

**Fig. 1**

**The position of selected cyanobacteria and algae taxa within a simplified phylogenetic tree.** The phylogenetic tree was built based on different data sources from the whole chloroplast genome and nuclear rDNA (Riisberg *et al.*, 2009; Ruhfel *et al.*, 2014). For complete list of analysed species see Table S1.

**Fig. 2**

**Endogenous cytokinin and auxin profiles in selected cyanobacteria and algae species.** The cytokinin profiles were determined in the early stationary growth phase and are presented based on the conjugation status/physiological function (Fig. 2A) and the chemical structure of the purine ring (Fig. 2B). Endogenous levels of both cytokinins and auxins (Fig. 2C) are expressed in pmol g^-1^ FW. Abbreviations of selected cyanobacteria and algae representatives are given in Table S1.

*trans*Z = *trans*-zeatin; DHZ = dihydrozeatin; *cis*Z = *cis-*zeatin; iP = *N^6^*-(Δ^2^-isopentenyl)adenine; 2MeSiPR = 2-methylthio-*N^6^*-(Δ^2^-isopentenyl)adenosine; IAA = indole-3-acetic acid; IAA-Asp = indole-3-acetic acid aspartate; OxIAA = 2-oxindole-3-acetic acid (abbreviations of cytokinins adopted and modified according to Kamínek *et al*., 2000).

**Fig. 3**

**Metabolism of exogenously applied [^3^H]*trans*Z in the cells of selected cyanobacteria and algae species.** Radiolabelled [^3^H]*trans*Z was exogenously applied to the cultures of cyanobacteria *Chroococcus minutus* (Fig. 3A) and algae *Scenedesmus obliquus* (Fig. 3B), *Chlororcoccum elbense* (Fig. 3C) and *Klebsormidium flaccidum* (Fig. 3D) in the early stationary growth phase. The peaks represent distribution of radioactivity associated with individual metabolites in the cells 0, 1, 4 and 24 h after [^3^H]*trans*Z application. The products of [^3^H]*trans*Z metabolism were analysed by HPLC coupled to on-line radioactivity detector.

*trans*Z = *trans*-zeatin; DHZ = dihydrozeatin; DHZR = dihydrozeatin 9-riboside; Ado = adenosine (abbreviations of cytokinins adopted and modified according to Kamínek *et al*., 2000).

**Fig. 4**

**Metabolism of exogenously applied [^3^H]IAA in the cells of selected algae species.** Radiolabelled [^3^H]IAA was exogenously applied to the cultures of Chlorophyta species *Stigeoclonium helveticum* (Fig. 4A), *Chlorella vulgaris* (Fig. 4B), *Microthamnion kuetzingianum* (Fig. 4C) and *Scenedesmus obliquus* (Fig. 4D) in the early stationary growth phase. The bars represent distribution of radioactivity associated with individual metabolites in the cells 0, 1, 2 and 6 h after [^3^H]IAA application. The products of [^3^H]IAA metabolism were analysed by HPLC coupled to on-line radioactivity detector. Values are expressed as percentage of the total extracted radioactivity in the cells.

IAA = indole-3-acetic acid; IAA-Asp = indole-3-acetic acid aspartate; IAA-GE = indole-3-acetyl-1-glucosyl ester.

**Fig. 5**

**Metabolism of exogenously applied [^3^H]IAA in the culture media of selected algae species.** Radiolabelled [^3^H]IAA was exogenously applied to the cultures of Chlorophyta species *Stigeoclonium helveticum* (Fig. 5A), *Chlorella vulgaris* (Fig. 5B), *Microthamnion kuetzingianum* (Fig. 5C) and *Scenedesmus obliquus* (Fig. 5D) in the early stationary growth phase. The bars represent distribution of radioactivity associated with individual metabolites in the media 0, 1, 2 and 6 h after [^3^H]IAA application. The products of [^3^H]IAA metabolism were analysed by HPLC coupled to on-line radioactivity detector. Values are expressed as percentage of the total extracted radioactivity in the media.

IAA = indole-3-acetic acid; IAA-Asp = indole-3-acetic acid aspartate; IAA-GE = indole-3-acetyl-1-glucosyl ester.

**Fig. 6**

**Growth characteristics and endogenous cytokinin and auxin profiles in the course of *Scenedesmus obliquus* growth cycle.** The *S. obliquus* growth cycle was characterised by the dry weight (DW) and the cell number (CN) increase (Fig. 6A), variations in the profiles of endogenous free cytokinins presented based on the conjugation status/physiological function (Fig. 6B) and the chemical structure of the purine ring (Fig. 6C) as well as tRNA-bound cytokinins (Fig. 6D) including methylthioderivatives (Fig. 6E) and changes in the spectra and concentrations of endogenous auxins (Fig. 6F). The levels of both free and tRNA-bound cytokinins and auxins are expressed in pmol g^-1^ DW.

*cis*Z = *cis-*zeatin; *trans*Z = *trans*-zeatin; iP = *N^6^*-(Δ^2^-isopentenyl)adenine; DHZ = dihydrozeatin; *cis*ZR = *cis-*zeatin 9-riboside; *trans*ZR = *trans*-zeatin 9-riboside; iPR = *N^6^*-(Δ^2^-isopentenyl)adenosine; DHZR = dihydrozeatin 9-riboside; 2MeS*cis*ZR = 2-methylthio-*cis-*zeatin 9-riboside; 2MeSiPR = 2-methylthio-*N^6^*-(Δ^2^-isopentenyl)adenosine (abbreviations of cytokinins adopted and modified according to Kamínek *et al*., 2000); IAA = indole-3-acetic acid; OxIAA = 2-oxindole-3-acetic acid; PAA = phenyl acetic acid; IAM = indole-3-acetamide.

**SUPPLEMENTARY DATA**

Supplementary data are available in the online version of this article at [www.aob.oxfordjournals.org](http://www.aob.oxfordjournals.org).

**Table S1**:

The list and abbreviations of cyanobacteria and algae species analysed for endogenous cytokinin and auxin profiles in this study.

**Table S2**:

Endogenous cytokinin spectra and concentrations (in pmol g^-1^ FW) in selected cyanobacteria and algae species in the early stationary growth phase. Abbreviations of selected representatives as given in Table S1; abbreviations of cytokinins adopted and modified according to Kamínek *et al*. (2000).

**Table S3**:

Endogenous auxin spectra and concentrations (in pmol g^-1^ FW) in selected cyanobacteria and algae species in the early stationary growth phase. Abbreviations of selected representatives as given in Table S1.

**Table S4**:

Endogenous spectra and concentrations of tRNA-bound cytokinins (related to pmol mg^-1^ tRNA) during *Scenedesmus obliquus* growth cycle. Abbreviations of cytokinins adopted and modified according to Kamínek *et al*. (2000).

**Table S5**:

Endogenous spectra and concentrations of auxins (in pmol g^-1^ FW) during *Scenedesmus obliquus* growth cycle.

**Figure S1**:

Metabolism of exogenously applied [^3^H]*trans*Z in the culture medium of *Klebsormidium flaccidum* in the early stationary growth phase. The peaks represent distribution of radioactivity associated with individual metabolites in the medium 4 and 24 h after [^3^H]*trans*Z application. The products of [^3^H]*trans*Z metabolism were analysed by HPLC coupled to on-line radioactivity detector.

*trans*Z = *trans*-zeatin; DHZ = dihydrozeatin.

**Figure S2**:

Metabolic conversion of [^3^H]*N^6^*-(Δ^2^-isopentenyl)adenine incubated *in vitro* with enzyme cytokinin oxidase/dehydrogenase preparations extracted and partially purified from selected cyanobacteria and algae species in the early stationary growth phase. The *in vitro* assays were performed in 100 mM MOPS-NaOH buffer containing 75 μM 2,6-dichloroindophenol at pH 7.0.

**Figure S3**: Metabolic conversion of [^3^H]*N^6^*-(Δ^2^-isopentenyl)adenine incubated *in vitro* with enzyme cytokinin oxidase/dehydrogenase preparations extracted and partially purified from selected cyanobacteria and algae species in the early stationary growth phase. The *in vitro* assays were performed in 100 mM TAPS-NaOH buffer containing 75 μM 2,6-dichloroindophenol at pH 8.5.

**FUNDING**

This work was supported by the Czech Science Foundation (16-14649S and GA15-22322S); by the Program of Postdoctoral Fellowship from the Czech Academy of Sciences (EŽ; MK) and by the long-term research development project [RVO 67985939]. Part of this work was also funded by the Ministry of Education, Youth and Sports of the Czech Republic through the National Program of Sustainability (grant no. LO1204) and by the internal Grant Agency of Palacký University (IGA PrF 2016 011).

**ACKNOWLEDGEMENTS**

The authors wish to thank Dr Miroslav Kamínek for critical reading of the manuscript, BSc Andrew Leppard for language editing and Bc Marie Korecká for her excellent and invaluable technical support.

**LITERATURE CITED**

**Abdel-Raouf N, Al-Homaidan AA, Ibraheem IBM. 2012.** Agriculture importance of algae. *African Journal of Biotechnology* **11**: 11648-11658.

**Abe H, Uchiyama M, Sato R. 1974.** Isolation of phenylacetic acid and its p-hydroxy derivative as auxin-like substances from *Undaria pinnatifida*. *Agricultural and Biological Chemistry* **38**: 897–898.

**Anantharaman V, Aravind L. 2001.** The CHASE domain: a predicted ligand-binding module in plant cytokinin receptors and other eukaryotic and bacterial receptors. *Trends in Biochemical Sciences* **26**: 579-582.

**Ashen, JB, Cohen JD, Goff LJ. 1999.** GC-SIM-MS detection and quantiﬁcation offree indole-3-acetic acid in bacterial galls on the marine alga Prionitislanceolata (Rhodophyta). *Journal of Phycology* **35**: 493-500.

**Benková E, Ivanchenko MG, Friml J, Shishkova S, Dubrovsky JG. 2009.** A morphogenetic trigger: is there an emerging concept in plant developmental biology? *Trends in Plant Science* **14**: 189-193.

Blilou I, Xu J, Wildwater M, Willemsen V, Paponov I, Friml J, Heidstra R, Aida M, Palme K, Scheres B. 2005. The PIN auxin efflux facilitator network controls growth and patterning in Arabidopsis roots. *Nature* 433: 39-44.

**Coenen C, Lomax TL.** **1997.** Auxin-cytokinin interactions in higher plants: old problems and new tools. *Trends in Plant Science* **2**: 351-356.

**Cooke TJ, Poli DB, Sztein AE, Cohen JD. 2002.** Evolutionary patterns in auxin action. *Plant Molecular Biology* **49**: 319-338.

**Cheng Y, Dai X, Zhao Y.** **2006.** Auxin biosynthesis by the YUCCA flavin monooxygenases controls the formation of floral organs and vascular tissues in Arabidopsis. *Genes Development* **20**: 1790-1799.

**Cheng Y, Dai X, Zhao Y.** **2007.** Auxin synthesized by the YUCCA flavin monooxygenases is essential for embryogenesis and leaf formation in Arabidopsis. *Plant Cell* **19**: 2430-2439.

**Czerpak R, Bajguz A, Bialecka B, Wierzcholowska LE, Wolanska MM. 1994.** Effect of auxin precursors and chemical analogs on the growth and chemical-composition in Chlorella-pyrenoidosa chick. *Acta Societatis Botanicorum Poloniae* **6**: 279-286.

**Djilianov DL, Dobrev PI, Moyankova DP, Vaňková R, Georgieva DT, Gajdošová S, Motyka V. 2013.** Dynamics of endogenous phytohormones during dessication and recovery of the resurrection plant species *Haberlea rhodopensis*. *Journal of Plant Growth Regulation* **32**: 564-574.

**Dobrev PI, Kamínek M. 2002.** Fast and efficient separation of cytokinins from auxin and abscisic acid and their purification using mixed-mode solid-phase extraction. *Journal of Chromatography A* **950**: 21-29.

**Dobrev PI, Havlíček L, Vágner M, Malbeck J, Kamínek M. 2005.** Purification and determination of plant hormones auxin and abscisic acid using solid phase extraction and two-dimensional high performance liquid chromatography. *Journal of Chromatography A* **1075**: 159-166.

**Frébort I, Kowalska M, Hluska T, Frébortová J, Galuszka P. 2011.** Evolution of cytokinin biosynthesis and degradation. *Journal of Experimental Botany* **62**:2431-2452.

**Frébortová J, Greplová M, Seidl MF, Heyl A, Frébort I.** **2015.** Biochemical characterization of puative adenylate dimethylallyltransferase and cytokinin dehydrogenase from Nostoc sp. PCC 7120. *PLoS ONE* **10**: e0138468.

**Friml J, Vieten A, Sauer M, Weijers D, Schwarz H, Hamann T, Offriga R, Jürgen G. 2003.** Efflux-dependent auxin gradients establish the apical-basal axis of Arabidopsis. *Nature* **426**: 147-153.

**Gajdošová S, Spíchal L, Kamínek M, Hoyerová K, Novák O, Dobrev PI, Galuszka P, Klíma P, Gaudinová A, Žižková E, Hanuš J, Dančák M, Trávníček B, Pešek B, Krupička M, Vaňková R, Strnad M, Motyka V. 2011.** Distribution, biological activities, metabolism, and the conceivable function of *cis-*zeatin-type cytokinins in plants. *Journal of Experimental Botany* **62**: 2827-2840.

**Gaudinová A, Dobrev PI, Šolcová B, Novák O, Strnad M, Friedecký D, Motyka V. 2005.** The involvement of cytokinin oxidase/dehydrogenase and zeatin reductase in regulation of cytokinin levels in pea (*Pisum sativum* L.) leaves. *Journal of Plant Growth Regulation* **24**: 188-200.

**Hashtroudi MS, Ghassempour A, Riahi H, Shariatmadari Z, Khanjir M.** **2013.** Endogenous auxins in plant growth-promoting Cyanobacteria – *Anabaena vaginicola* and *Nostoc calcicola*. *Journal of Applied Phycology* **25**: 379-386.

**Hussain A, Krischke M, Roitsch T, Hasnain S. 2010.** Rapid determination of cytokinins and auxin in cyanobacteria. *Current Microbiology* **61**: 361-369.

**Hwang I, Sakakibara H. 2006.** Cytokinin biosynthesis and perception. *Physiologia Plantarum* **126**: 528-538.

**Hwang I, Sheen J, Müller B. 2012.** Cytokinin signaling networks. *Annual Review of Plant Biology* **63**: 353-380.

**Jusoh M, Loh SH, Chuah TS, Aziz A, Cha TS.** **2015.** Indole-3-acetic acid (IAA) induced changes in iol content, fatty acid profiles and expression of four fatty acid biosynthetic genes in Chlorella vulgaris at early stationary growth phase. *Phytochemistry* **111**: 65-71.

**Kai K, Horita J, Wakasa K, Miyagawa H. 2007a.** Three oxidative metabolites of indole-3-acetic acid from *Arabidopsis thaliana.* *Phytochemistry* **68**: 1651-1663.

**Kai K, Nakamura S, Wakasa K, Miyagawa H. 2007b.** Facile preparation of deuterium-labeled standards of indole-3-acetic acid (IAA) and its metabolites to quantitatively analyze the disposition of exogenous IAA in *Arabidopsis thaliana. Bioscience Biotechnology and Biochemistry* **71**: 1946-1954.

**Kakimoto T.** **2003.** Biosynthesis of cytokinins. *Journal of Plant Research* 116: 233-239.

**Kamínek M, Březinová A, Gaudinová A, Motyka V, Vaňková R, Zažímalová E.** **2000.** Purine cytokinins: a proposal of abbreviations. *Plant Growth Regulation* **32:** 253-256.

**Kenrick P, Crane PR. 1997.** The origin and early evolution of plants on land. *Nature* **389**: 33-39.

**Kieber JJ, Schaller GE. 2014.** Cytokinins. *The Arabidopsis Book* 11:e0168. doi:10.1199/tab.0168.

**Kiseleva AA, Tarachvskaya ER, Shishova MF. 2012.** Biosynthesis of phytohormones in algae. *Russian Journal of Plant Physiology* **59**: 595-610.

**Korasick DA, Enders TA, Strader LC.** **2013.** Auxin biosynthesis and storage forms. *Journal of Experimental Botany* **64**: 2541-2555.

**Le Bail A, Billoud B, Kowalczyk N, Kowalczyk M, Gicquel M, Le Panse S, Stewart S, Scornet D, Cock JM, Ljung K, Charrier B**. **2010.** Auxin metabolism and function in the multicellular brown alga *Ectocarpus siliculosus*. *American Society of Plant Biologists* **153**: 128-144.

**Lewis LA, McCourt RM. 2004.** Green algae and the origin of land plants. *American Journal of Botany* **91**: 1535–1556.

**Ljung K, Hull AK, Kowalczyk M, Marchant A, Celenza J, Cohen JD, Sandberg G. 2002.** Biosynthesis, conjugation, catabolism and homeostasis of indole-3-acetic acid in *Arabidopsis thaliana. Plant Molecular Biology* **49**: 249-272.

**Ljung K.** **2013.** Auxin metabolism and homeostasis during plant development. *Development* **140**: 943-950.

**Löbler M, Klämbt D. 1985.** Auxin-binding protein from coleoptile membranes of corn (*Zea mays* L.). I. Purification by immunological methods and characterization. *Journal of Biological Chemistry* **260**: 9848-9853.

**Lu Y, Tarkowská D, Turečková V, Luo T, Xin Y, Li J, Wang Q, Jiao N, Strnad M, Xu J. 2014.** Antagonistic roles of abscisic acid and cytokinin during response to nitrogen depletion in oleaginous microalga *Nannochloropsis oceanica* expand the evolutionary breadth of phytohormone function. *The Plant Journal* **80**: 52-68.

**Lu Y, Xu J. 2015.** Phytohormones in microalgae: a new opportunity for microalgal biotechnology? *Trends in Plant Science* **20**: 273-282.

**Ludwig-Müller J**. **2011.** Auxin conjugates: their role for plant development and in the evolution of land plants. *Journal of Experimental Botany* **62**: 1757-1773.

**Maass H**, **Klämbt D**. **1981.** On the biogenesis of cytokinins in roots of *Phaseolus vulgaris*. *Planta* **151:** 353–358.

**Mähönen AP, Tusscher K, Siligato R, Smetana O, Díaz-Triviño S, Salojärvi J, Wachsman G, Prasad K, Heidstra R, Scheres B. 2014.** PLETHORA gradient formation mechanism separates auxin responses. *Nature* **515**: 125-129.

**Maor R. 2010.** Compositions and methods for increasing oil content in algae. European Patent Application WO2010IL00247 20100324.

**Martin RC, Mok MC, Shaw G, Mok DWS. 1989.** An enzyme mediating the conversion of zeatin to dihydrozeatin in phaseolus embryos. *Plant Physiology* **90**: 1630-1635.

**Mazhar S, Cohen JD, Hasnain S. 2013.** Auxin producing non-heterocystous cyanobacteria and their impact on the growth and endogenous auxin homeostasis of wheat. *Journal of Basic Microbiology* **53**: 996–1003.

**Mazur H., Konop A, Synak R. 2001.** Indole-3-acetic acid in the culture medium of two axenic green microalgae. *Journal of Applied Phycology* **13**: 35–42.

**Miller CO, Skoog F, von Saltza MH, Strong FM. 1956.** Isolation, structure and synthesis of kinetin, a substance promoting cell division. *Journal of American Chemical Society* **78**: 1375-1380.

**Morrison EN, Knowles S, Hayward A, Thorn RG, Saville BJ, Emery RJN. 2015.** Detection of phytohormones in temperate forest fungi predicts consistent abscisic acid production and a common pathway for cytokinin biosynthesis. *Mycologia* **107**: 245-257.

**Motyka V, Vaňková R, Čapková V, Petrášek J, Kamínek M, Schmülling T. 2003.** Cytokinin-induced upregulation of cytokinin oxidase activity in tobacco includes changes in enzyme glycosylation and secretion. *Physiologia Plantarum* **117**: 11-21.

**Normanly J.** 2010. Approaching cellular and molecular resolution of auxin biosynthesis and metabolism. *Cold Spring Harbor Perspectives in Biology* **2**: a001594.

**Normanly J, Cohen JD, Fink GR.** 1993. *Arabidopsis thaliana* auxotrophs reveal a tryptophan-independent biosynthetic pathway for indole-3-acetic acid. *Proceedins of the National Academy of Sciences of the United States of America* **21**: 10355-10359.

**Novák O**, **Tarkowski P**, **Tarkowská D**, **Doležal K**, **Lenobel R**, **Strnad M. 2003.** Quantitative analysis of cytokinins in plants by liquid chromatography–single-quadrupole mass spectrometry. *Analytica Chimica Acta* **480:** 207–218.

**Novák O**, **Hauserová E**, **Amakorová P**, **Doležal K**, **Strnad M. 2008.** Cytokinin profiling in plant tissues using ultra-performance liquid chromatography-electrospray tandem mass spectrometry. *Phytochemistry* **69:** 2214-2224.

**Nowak J., Sonaike B, Lawson GW. 1988.** Auxin induced stress tolerance in algae. *Environmental Pollution* **51**: 213–218.

**Ouyang J, Shao X, Li J.** **2000.** Indole-3-glycerol phosphate, a branchpoint of indole-3-acetic acid biosynthesis from the tryptophan biosynthetic pathway in *Arabidopsis thaliana*. *Plant Journal* **24**: 327-334.

**Őrdőg V, Stirk WA, van Staden J, Novák O, Strnad M. 2004.** Endogenous cytokinins in three genera of microalgae from the Chlorophyta. *Journal of Phycology* **40**: 88-95.

**Östin A, Kowalyczk M, Bhalerao RP, Sandberg G. 1998.** Metabolism of indole-3-acetic acid in Arabidopsis. *Plant Physiology* **118**: 285–296.

**Park W, Yoo G, Moon M, Kim C, Choi YE, Yang JW. 2013.** Phytohormone supplementation signiﬁcantly increases growth of Chlamydomonas reinhardtii cultivated for biodiesel production. *Applied Biochemistry and Biotechnology* **171**: 1128–1142.

**Pernisová M, Kuderová A, Hejátko J.** **2011.** Cytokinin and auxin interactions in plant development: metabolism, signalling, transport and gene expression. *Current Protein and Peptide Science* **12**: 137-147.

**Pils B, Heyl A. 2009.** [Unraveling the Evolution of Cytokinin Signaling. *Plant Physiology* **151**: 782-791.](http://apps.webofknowledge.com/full_record.do?product=UA&search_mode=GeneralSearch&qid=101&SID=W2ixOZkySocgBBLTJ15&page=1&doc=1)

**Piotrowska-Niczyporuk A, Bajguz A. 2014.** The effect of natural and synthetic auxins on the growth, metabolite content and antioxidant response of green alga *Chlorella vulgaris* (Trebouxiophyceae). *Plant Growth Regulation* **73**: 57–66.

**Přibyl P, Cepák V, Kaštánek P, Zachleder V. 2015.** Elevated production of carotenoids by a new isolate of *Scenedesmus* sp. *Algal Research* **11**: 22-27.

**Prinsen E, Kamínek M, van Onckelen HA. 1997.** [Cytokinin biosynthesis: a black box?](http://apps.webofknowledge.com/full_record.do?product=UA&search_mode=GeneralSearch&qid=103&SID=W2ixOZkySocgBBLTJ15&page=1&doc=3) *Plant Growth Regulation* **23**: 3-15.

**Rocha OP, Felício R, Rodrigues AHB, Ambrósio DL, Cicarelli RMB, Albuquerque S, Young MCM, Yokoya NS, Debonsi HM. 2011.** Chemical Profile and Biological Potential of Non-Polar Fractions from *Centroceras clavulatum* (C. Agardh) Montagne (Ceramiales, Rhodophyta). *Molecules* **16**: 7105-7114.

**Riisberg I, Orr RJS, Kluge R, Shalchian-Tabrizi K, Bowers HA, Patil V, Edvardsen B, Jakobsen KS. 2009.** Seven gene phylogeny of heterokonts. *Protist* **160**: 191–204.

**Ruhfel BR, Gitzendanner MA, Soltis PS, Soltis DE, Burleigh JG. 2014.** From algae to angiosperms –inferring the phylogeny of green plants (Viridiplantae) from 360 plastid genomes. *BMC Evolutionary Biology* **14**: doi: 10.1186/1471-2148-14-23.

**Sakakibara H. 2006.** Cytokinins: Activity, biosynthesis, and translocation. *Annual Review of Plant Biology* **57**: 431-49.

**Santner A, Calderon-Villalobos LIA, Estelle M. 2009.** Plant hormones are versatile chemical regulators of plant growth. *Nature Chemical Biology* **5**: 301-307.

**Schmülling T, Werner T, Riefler M, Krupková E, Bartriba y Manns I. 2003.** [Structure and function of cytokinin oxidase/dehydrogenase genes of maize, rice, Arabidopsis and other species. *Journal of Plant Research* **116**: 241-252.](http://apps.webofknowledge.com/full_record.do?product=UA&search_mode=GeneralSearch&qid=88&SID=W2ixOZkySocgBBLTJ15&page=1&doc=4)

**Schneider EA, Kazakoff CW, Wightman F. 1985.** Gas chromatography–mass spectrometry evidence for several endogenous auxins in pea seedling organs. *Planta* **165**: 232–241.

**Selivankina SY, Zubkova NK, Kupriyanova EV, Lyukevich TV, Kusnetsov VV, Los DA, Lulaeva ON. 2006.** Cyanobacteria respond to cytokinin. *Russian Journal of Plant Physiology* **53**: 751-755.

**Sergeeva E, Liaimer A, Bergman B. 2002.** Evidence for production of the phytohormoneindole-3-acetic acid by cyanobacteria. *Planta* **215**: 229–238.

**Shimizu-Mitao Y, Kakimoto T. 2014.** Auxin sensitivities of all Arabidopsis Aux/IAAs for degradation in the presence of every TIR1/AFB. *Plant Cell Physiology* **55**: 1450-1459.

**Spíchal L. 2012.** Cytokinins – recent news and views of evolutionally old molecules. *Functional Plant Biology* **39**: 267-284.

**Stirk WA, Ördög V, van Staden J. 1999.** Identification of the cytokinin isopentenyladenine in a strain of *Arthronema africanum* (Cyanobacteria). *Journal of Phycology* **35**: 89-92.

**Stirk WA, Ördög V, van Staden J, Jäger K. 2002.** Cytokinin- and auxin-like activity in Cyanophyta and microalgae. *Journal of Applied Phycology* **14**: 215-221.

**Stirk WA, Novák O, Strnad M, van Staden J. 2003.** Cytokinins in macroalgae. *Plant Growth Regulation* **41**: 13-24.

**Stirk WA, van Staden J, Novák O, Doležal K, Strnad M, Dobrev PI, Sipos G, Ordog V, Balint P. 2011.** Changes in endogenous cytokinin concentrations in *Chlorella* (Chlorophyceae) in regulation to light and the cell cycle. *Journal of Phycology* **47**: 291-301.

**Stirk WA, Václavíková K, Novák O, Gajdošová S, Kotland O, Motyka V, Strnad M, Van Staden J.** **2012.** Involvement of *cis*-zeatin, dihydrozeatin, and aromatic cytokinins in germination and seedling establishement of maize, oats and lucerne. *Journal of Plant Growth Regulation* **31**: 392-405.

**Stirk WA, Ördög V, Novák O, Rolčík J, Strnad M, Van Staden J. 2013.** Auxin and cytokinin relationships in 24 microalgal strains. *Journal of Phycology* **49**: 459-467.

**Stirk WA, Tarkowska D, Turecova V, Strnad M, van Staden J. 2014.** Abscisic acid, gibberellins and brassinosteroids in Kelpak (R), a commercial seaweed extract made from *Ecklonia maxima*. *Journal of Applied Phycology* **26**: 561-567.

**Sugawara S, Mashiguchi K, Tanaka K, Hishiyama S, Sakai T, Hanada K, Kinoshita-Tsujimura K, Yu H, Dai X, Takebayashi Y, Takeda-Kamiya N, Kakimoto T, Kawaide H, Natsume M, Estelle M, Zhao Y, Hayashi K, Kamiya Y , Kasahara H. 2015.** Distinct Characteristics of Indole-3-Acetic Acid and Phenylacetic Acid, Two Common Auxins in Plants. *Plant Cell Physiology* **56**: 1641-1654.

**Svačinová J**, **Novák O**, **Plačková L**, **Lenobel R**, **Holík J**, **Strnad M**, **Dolezal K.** **2012.** A new approach for cytokinin isolation from Arabidopsis tissues using miniaturized purification: pipette tip solid-phase extraction. *Plant Methods* **8**: 17.

**Swaminathan S, Bock RM. 1977.** Isolation and identification of cytokinins from *Euglena gracilis* transfer ribonucleic acid. *Biochemistry* **16**: 1355-1360.

**Šimura J, Novák O, Strnad M, Nedbal L.** **2014.** Cytokinin profiling in Cyanobacteria and microalgae species using UHPLC-MS/MS. In: Book of Abstracts of the International Symposium 2014 “Auxins and Cytokinins in Plant Development….and Interactions with Other Phytohormones”, Prague, Czech Republic, p. 31 (P1-15).

**Takei K, Yamaya T, Sakakibara H.** **2004.** [Arabidopsis CYP735A1 and CYP735A2 encode cytokinin hydroxylases that catalyze the biosynthesis of trans-Zeatin. *Journal of* *Biological Chemistry* **279**: 41866-41872.](http://apps.webofknowledge.com/full_record.do?product=UA&search_mode=GeneralSearch&qid=106&SID=W2ixOZkySocgBBLTJ15&page=1&doc=1)

**Tarakhovskaya ER, Maslov YI, Shishova MF. 2007.** Phytohormones in algae. *Russian Journal of Plant Physiology* **54**: 186-194.

**Tarkowski P, Václavíková K, Novák O, Pertry I, Hanuš J, Whenham R, Vereecke D, Šebela M, Strnad M. 2010.** Analysis of 2-methylthio-derivatives of isoprenoid cytokinins by liquid chromatography-tandem mass spectrometry. *Analytica Chimica Acta* **680**: 86-91.

**Tivendale ND, Ross JJ, Cohen JD.** **2014.** The shifting paradigms of auxin biosynthesis. *Trends in Plant Science* **19**: 44-51.

**Yevdakova NA, Motyka V, Malbeck J, Trávníčková A, Novák O, Strnad M, von Schwartzenberg K. 2008.** Evidence for importance of tRNA-dependent cytokininbiosynthetic pathway in the moss *Physcomitrella patens*. *Journal of Plant Growth Regulation* **27:** 271-281.

**Yokoya NS, Stirk WA, van Staden J, Novák O, Turečková V, Pěnčík A, Strnad M. 2010.** Endogenous cytokinins, auxins and abscisic acid in red algae from Brazil. *Journal of Phycology* **46**: 1198-1205.

**Yokoya NS, Yoneshigue-Valentin Y. 2011.** Micropropagation as a tool for sustainable utilization and conservation of populations of Rhodophyta. *Revista Brasileira De Farmacognosia-Brazilian Journal of Pharmacognosy* **21**: 334-339.

**Yue J, Xiangyang H, Huang J.** **2014.** Origin of plant auxin biosynthesis. *Trends in Plant Science* **19:** 764-770.

**Varalakshmi P, Malliga P.** **2012.** Evidence for production of indole-3-acetic acid from a fresh water cyanobacteria (*Oscillatoria annae*) on the growth of *H. annus*. *International Journal of Scientific and Research Publications* **3**: 1-15.

**Wang B, Chu J, Yu T, Xu Q, Sun X, Yuan J, Xiong G, Wang G, Wang Y, Li J.** **2015.** Tryptophan-independent auxin biosnthesis contributes to early embryogenesis in Arabidopsis. *Proceedins of the National Academy of Sciences of the United States of America* **112**: 4821-4826.

**Wightman F, Lighty DL. 1982.** Identification of phenylacetic acid as natural auxin in the shoots of higher plants. *Physiologia Plantarum* **55**: 17-24.

**Woodward AW, Bartel B. 2005.** Auxin: regulation, action, and interaction. *Annals of Botany* **95**: 707–735.

**Záveská Drábková L, Dobrev PI, Motyka V. 2015.** Phytohormone profiling across the bryophytes. *PLoS ONE* **10**: e0125411.

**Žižková E, Dobrev PI, Muhovski Y, Hošek P, Hoyerová K, Lutts S, Motyka V. 2015.** Tomato (*Solanum lycopersicum* L.) SlIPT3 and SlIPT4 isopentenyltransferases mediate salt stress response in tomato. *BMC Plant Biology* **15**: 85, doi: 10.1186/s12870-015-0415-7.

**TABLE S1**

| **Phyllum** | **Order** | **Class** | **CCALA #** | | **Species, authority** | **Abb.** |
| --- | --- | --- | --- | --- | --- | --- |
| Cyanobacteria | Chroococcales | Cyanophyceae | 55 | *Chroococcus minutus* (Kuetzing) Naegeli | | **CM** |
|  | Nostocales | Cyanophyceae | 139 | *Phormidium animale* (C. Agardh ex Gomont) Anagnostidis et Komárek | | **PA** |
|  | Nostocales | Cyanophyceae | 124 | *Nostoc microscopicum* Carmichael | | **NM** |
| Ochrophyta | Tribonematales | Xanthophyceae | 512 | *Tribonema aequale* Pascher | | **TA** |
|  | Mischococcales | Xanthophyceae | 223 | *Bumilleriopsis filiformis* Vischer | | **BF** |
|  | Eustigmatales | Eustigmatophyceae | 514 | *Vischeria helvetica* (Vischer et Pascher) Hibberd | | **VH** |
| Rhodophyta | Porphyridiales | Porphyridiophyceae | 416 | *Porphyridium purpureum* (Bory de Saint-Vincent) K. M. Drew & R. Ross | | **PP** |
|  | Rhodellales | Porphyridiophyceae | 925 | *Rhodella violacea* (Kornmann) Wehrmeyer | | **RV** |
| Chlorophyta | Chlamydomonadales | Chlorophyceae | 283 | *Chlorococcum ellipsoideum* Deason et Bold | | **CE** |
|  | Chlamydomonadales | Chlorophyceae | 282 | *Chlorococcum elbense* Archibald | | **ChE** |
|  | Chlamydomonadales | Chlorophyceae | 421 | *Protosiphon botryoides* (Kuetzing) Klebs | | **PB** |
|  | Chlamydomonadales | Chlorophyceae | 248 | *Chlamydomonas segnis* Ettl | | **CS** |
|  | Chaetophorales | Chlorophyceae | 868 | *Stigeoclonium helveticum* Vischer | | **SH** |
|  | Sphaeropleales | Chlorophyceae | 454 | *Scenedesmus obliquus* (Turpin) Kuetzing | | **SO** |
|  | Ulotrichales | Ulvophyceae | 926 | *Ulothrix crenulata* Kuetzing | | **UC** |
|  | Ulvales | Ulvophyceae | 423 | *Pseudendoclonium basiliense* Vischer | | **PsB** |
|  | Prasiolales | Trebouxiophyceae | 420 | *Prasiolopsis ramosa* Vischer | | **PR** |
|  | Microthamniales | Trebouxiophyceae | 368 | *Microthamnion kuetzingianum* Naegeli | | **MK** |
| Streptophyta | Desmidiales | Zygnematophyceae | 836 | *Actinotaenium curtum* (Ralfs) Teiling ex Ruzicka et Pouzar | | **AC** |
|  | Klebsormidiales | Klebsormidiophyceae | 786 | *Klebsormidium flaccidum* (Kützing) P.C. Silva, K.R. Mattox et W.H. Blackwell | | **KF** |

**TABLE S2**

| **Phyllum** | **Species** | ***trans*Z** | ***trans*ZR** | ***trans*Z7G** | ***trans*Z9G** | ***trans*ZOG** | ***trans*ZROG** | ***trans*ZRMP** | **Σ*trans*Z-types** |
| --- | --- | --- | --- | --- | --- | --- | --- | --- | --- |
| Cyanobacteria | *Chroococcus minutus* (CM) | 0.51 ± 0.18 | - | - | - | - | - | 1.36 ± 0.28 | **1.87** |
|  | *Phormidium animale* (PA) | 0.43 ± 0.10 | - | - | - | - | - | 1.69 ± 0.68 | **2.12** |
|  | *Nostoc microscopicum* (NM) | 4.95 ± 3.06 | - | - | - | - | - | - | **4.95** |
| Ochrophyta | *Tribonema aequale* (TA) | 0.18 ± 0.05 | - | - | - | 0.02 ± 0.01 | - | 0.49 ± 0.36 | **2.81** |
|  | *Bumilleriopsis filiformis* (BF) | 0.79 ± 0.27 | 0.37 ± 0.30 | - | 0.38 ± 0.29 | - | 0.09 ± 0.02 | 1.33 ± 0.43 | **2.96** |
|  | *Vischeria helvetica* (VH) | 2.28 ± 0.73 | - | - | - | - | - | 30.34 ± 1.30 | **32.62** |
| Rhodophyta | *Porphyridium purpureum* (PP) | 0.55 ± 0.40 | 0.02 ± 0.00 | - | - | - | - | 0.98 ± 0.11 | **1.55** |
|  | *Rhodella violacea* (RV) | 23.93 ± 9.57 | - | - | - | - | - | 1.44 ± 0.78 | **25.37** |
| Chlorophyta | *Chlorococcum ellipsoideum* (CE) | 0.27 ± 0.07 | - | - | - | 0.06 ± 0.03 | 0.03 ± 0.00 | 1.17 ± 0.00 | **1.53** |
|  | *Protosiphon botryoides* (PB) | 0.30 ± 0.03 | - | - | - | - | - | - | **0.30** |
|  | *Chlamydomonas segnis* (CS) | 1.38 ± 0.23 | - | - | - | - | - | 0.46 ± 0.10 | **1.84** |
|  | *Stigeoclonium helveticum* (SH) | - | - | - | - | - | - | 6.89 ± 0.24 | **6.89** |
|  | *Scenedesmus obliquus* (SO) | 0.29 ± 0.08 | - | 0.02 ± 0.01 | 0.18 ± 0.03 | - | - | - | **0.49** |
|  | *Ulothrix crenulata* (UC) | 0.13 ± 0.02 | - | - | - | - | - | 1.65 ± 0.22 | **1.78** |
|  | *Pseudendoclonium basiliense* (PsB) | 1.02 ± 0.34 | - | - | - | - | - | 0.49 ± 0.08 | **1.51** |
|  | *Prasiolopsis ramosa* (PR) | 0.63 ± 0.41 | - | - | 0.25 ± 0.13 | - | 0.44 ± 0.38 | 1.33 ± 0.61 | **2.65** |
|  | *Microthamnion kuetzingianum* (MK) | 1.35 ± 0.03 | - | - | - | 0.02 ± 0.01 | - | - | **1.37** |
| Streptophyta | *Actinotaenium curtum* (AC) | - | - | - | - | - | - | 1.29 ± 0.01 | **1.29** |
|  | *Klebsormidium flaccidum* (KF) | 0.22 ± 0.06 | - | - | - | - | - | 13.08 ± 0.45 | **13.30** |

| **Species** | **DHZ** | **DHZR** | **DHZ9G** | **DHZOG** | **DHZRMP** | **ΣDHZ-types** | ***cis*Z** | ***cis*ZR** | **Σ*cis*Z-types** |
| --- | --- | --- | --- | --- | --- | --- | --- | --- | --- |
| **CM** | 0.36 ± 0.15 | 0.54 ± 0.15 | - | - | 0.24 ± 0.11 | **1.14** | 2.42 ± 0.37 | - | **2.42** |
| **PA** | 0.55 ± 0.26 | 0.63 ± 0.50 | - | - | - | **1.18** | 0.39 ± 0.06 | - | **0.39** |
| **NM** | 0.50 ± 0.36 | - | - | - | - | **0.50** | 0.98 ± 0.16 | 0.49 ± 0.35 | **1.47** |
| **TA** | 0.32 ± 0.07 | 0.52 ± 0.01 | 0.07 ± 0.02 | - | 1.07 ± 0.45 | **1.98** | - | - | **-** |
| **BF** | 0.41 ± 0.16 | - | - | - | 1.00 ± 0.05 | **1.41** | 7.77 ± 0.54 | 0.66 ± 0.11 | **8.43** |
| **VH** | 0.35 ± 0.03 | 0.31 ± 0.10 | - | - | 2.78 ± 0.10 | **3.44** | 3.69 ± 0.38 | 7.77 ± 1.06 | **11.46** |
| **PP** | 0.34 ± 0.10 | - | 0.04 ± 0.03 | - | 0.15 ± 0.05 | **0.53** | - | - | **-** |
| **RV** | 0.35 ± 0.09 | - | - | - | - | **0.35** | 0.32 ± 0.12 | 0.77 ± 0.17 | **1.09** |
| **VCE** | 0.19 ± 0.06 | - | - | - | 0.61 ± 0.16 | **0.80** | 0.46 ± 0.05 | 0.32 ± 0.02 | **0.78** |
| **PB** | 0.24 ± 0.11 | 0.14 ± 0.07 | - | - | 1.13 ± 0.19 | **1.51** | 0.74 ± 0.18 | 0.19 ± 0.06 | **0.93** |
| **CS** | 0.37 ± 0.01 | 0.16 ± 0.04 | 0.20 ± 0.03 | 0.06 ± 0.01 | - | **0.79** | 55.91 ± 5.61 | - | **55.91** |
| **SH** | 0.24 ± 0.02 | 0.26 ± 0.19 | - | - | 3.45 ± 0.75 | **3.95** | 0.99 ± 0.05 | 1.74 ± 0.31 | **2.73** |
| **SO** | 0.31 ± 0.00 | 0.34 ± 0.01 | - | - | 0.71 ± 0.21 | **1.36** | 3.65 ± 0.87 | 2.30 ± 1.00 | **5.95** |
| **UC** | 0.37 ± 0.11 | 0.37 ± 0.10 | 0.10 ± 0.06 | - | 1.20 ± 0.06 | **2.04** | - | 0.37 ± 0.03 | **0.37** |
| **PsB** | 0.24 ± 0.03 | 0.45 ± 0.06 | - | - | 0.41 ± 0.02 | **1.10** | 10.93 ± 1.24 | - | **10.93** |
| **PR** | 0.31 ± 0.04 | 0.21 ± 0.15 | - | - | 3.00 ± 0.10 | **3.52** | 0.54 ± 0.08 | - | **0.54** |
| **MK** | 0.46 ± 0.12 | 0.20 ± 0.04 | - | - | - | **0.66** | 9.69 ± 1.22 | - | **9.69** |
| **AC** | - | 0.83 ± 0.19 | - | - | - | **0.83** | 0.68 ± 0.22 | 0.22 ± 0.00 | **0.90** |
| **KF** | 0.53 ± 0.17 | 0.51 ± 0.13 | 0.07 ± 0.05 | - | 2.74 ± 1.12 | **3.85** | 1.13 ± 0.12 | 4.13 ± 0.95 | **5.26** |

| **Species** | **iP** | **iPR** | **iP7G** | **iP9G** | **iPRMP** | **ΣiP-types** | **2MeSiPR** | **Total CKs** |
| --- | --- | --- | --- | --- | --- | --- | --- | --- |
| **CM** | 1.37 ± 0.14 | 0.67 ± 0.16 | - | - | 2.89 ± 0.15 | **4.93** | 3.43 ± 0.16 | **13.79** |
| **PA** | 12.21 ± 0.48 | 7.77 ± 0.19 | - | - | 63.67 ± 7.50 | **83.65** | 91.21 ± 1.94 | **178.55** |
| **NM** | - | - | - | - | 2.19 ± 1.70 | **2.19** | - | **9.11** |
| **TA** | 0.76 ± 0.03 | 0.17 ± 0.03 | - | 0.14 ± 0.10 | 7.37 ± 0.74 | **8.44** | 0.80 ± 0.10 | **14.03** |
| **BF** | 78.35 ± 2.31 | 11.71 ± 0.94 | - | 0.99 ± 0.05 | 7.73 ± 0.30 | **98.78** | 7.44 ± 0.18 | **119.02** |
| **VH** | 0.61 ± 0.05 | 2.99 ± 0.46 | - | - | 6.77 ± 0.61 | **10.37** | 9.14 ± 0.14 | **67.03** |
| **PP** | 0.10 ± 0.03 | - | - | - | 0.35 ± 0.02 | **0.45** | - | **2.53** |
| **RV** | 0.11 ± 0.09 | - | - | - | 0.54 ± 0.15 | **0.65** | 2.11 ± 0.98 | **29.57** |
| **VCE** | 0.28 ± 0.01 | - | - | - | 0.84 ± 0.21 | **1.12** | 1.41 ± 0.05 | **5.64** |
| **PB** | 20.50 ± 1.19 | 0.40 ± 0.19 | - | - | 0.25 ± 0.05 | **21.15** | 3.34 ± 1.54 | **27.23** |
| **CS** | 42.37 ± 1.18 | - | 0.01 ± 0.00 | - | 0.10 ± 0.02 | **42.48** | 0.98 ± 0.28 | **102** |
| **SH** | 0.36 ± 0.00 | - | 1.96 ± 0.24 | - | 2.79 ± 0.04 | **5.11** | 1.41 ± 0.01 | **20.09** |
| **SO** | 1.31 ± 0.29 | 1.44 ± 0.08 | - | 0.08 ± 0.02 | - | **2.83** | 7.04 ± 0.80 | **17.67** |
| **UC** | 0.04 ± 0.01 | 0.20 ± 0.05 | - | - | 0.27 ± 0.10 | **0.51** | 0.28 ± 0.21 | **4.98** |
| **PsB** | 12.26 ± 1.41 | 0.95 ± 0.17 | - | - | 0.32 ± 0.15 | **13.53** | 42.76 ± 8.47 | **69.83** |
| **PR** | 1.17 ± 0.17 | 0.86 ± 0.02 | - | - | 0.42 ± 0.05 | **2.45** | 0.70 ± 0.26 | **9.86** |
| **MK** | 3.25 ± 0.11 | - | - | - | 0.82 ± 0.04 | **4.07** | 8.10 ± 1.80 | **23.89** |
| **AC** | 1.08 ± 0.03 | - | - | - | 0.24 ± 0.07 | **1.32** | - | **4.34** |
| **KF** | 2.25 ± 0.24 | 0.28 ± 0.21 | - | 0.09 ± 0.05 | 1.70 ± 0.03 | **4.32** | 0.29 ± 0.00 | **27.02** |

**TABLE S3**

| **Phyllum** | **Species** | **IAA** | **IAA-Asp** | **OxIAA** | **Σauxins** |
| --- | --- | --- | --- | --- | --- |
| Cyanobacteria | *Chroococcus minutus* (CM) | 192.95 ± 5.90 | 0.03 ± 0.02 | 5.47 ± 0.12 | **198.45** |
|  | *Phormidium animale* (PA) | 250.17 ± 12.32 | 0.09 ± 0.05 | 9.51 ± 0.09 | **259.77** |
|  | *Nostoc microscopicum* (NM) | 9.40 ± 2.82 | 0.05 ± 0.03 | 5.24 ± 2.00 | **14.69** |
| Ochrophyta | *Tribonema aequale* (TA) | 86.36 ± 7.68 | - | 3.37 ± 0.72 | **89.73** |
|  | *Bumilleriopsis filiformis* (BF) | 11.00 ± 0.51 | - | 1.78 ± 0.09 | **12.78** |
|  | *Vischeria helvetica* (VH) | 29.82 ± 1.32 | 0.04 ± 0.01 | 3.16 ± 0.21 | **33.02** |
| Rhodophyta | *Porphyridium purpureum* (PP) | 3.26 ± 1.22 | 0.05 ± 0.03 | 13.19 ± 0.77 | **16.5** |
|  | *Rhodella violacea* (RV) | 11.18 ± 1.96 | - | 5.90 ± 0.59 | **17.8** |
| Chlorophyta | *Chlorococcum ellipsoideum* (CE) | 56.19 ± 3.91 | 0.22 ± 0.09 | 3.78 ± 0.26 | **60.19** |
|  | *Protosiphon botryoides* (PB) | 23.47 ± 2.30 | 0.10 ± 0.02 | 43.54 ± 4.77 | **67.11** |
|  | *Chlamydomonas segnis* (CS) | 13.39 ± 1.52 | - | 10.83 ± 1.65 | **91.34** |
|  | *Stigeoclonium helveticum* (SH) | 287.57 ± 19.89 | 0.03 ± 0.01 | 3.09 ± 0.45 | **290.69** |
|  | *Scenedesmus obliquus* (SO) | 19.59 ± 1.73 | 0.70 ± 0.17 | 27.32 ± 9.75 | **47.61** |
|  | *Ulothrix crenulata* (UC) | 27.17 ± 0.51 | - | 3.18 ± 0.30 | **30.35** |
|  | *Pseudendoclonium basiliense* (PsB) | 6.43 ± 0.52 | - | 4.50 ± 1.12 | **10.93** |
|  | *Prasiolopsis ramosa* (PR) | 148.72 ± 9.48 | 0.15 ± 0.04 | 4.53 ± 0.06 | **153.4** |
|  | *Microthamnion kuetzingianum* (MK) | 45.70 ± 1.29 | 0.21 ± 0.02 | 19.16 ± 3.74 | **65.07** |
| Streptophyta | *Actinotaenium curtum* (AC) | 113.34 ± 4.56 | 0.06 ± 0.03 | 2.76 ± 0.15 | **116.16** |
|  | *Klebsormidium flaccidum* (KF) | 13.61 ± 0.35 | - | 7.61 ± 0.66 | **21.22** |

**TABLE S4**

|  |  |  | pmol mg^-1^ tRNA | | | | pmol mg^-1^ tRNA | |
| --- | --- | --- | --- | --- | --- | --- | --- | --- |
| **Time (d)** | **ng tRNA/g DW** | **tRNA (ng/ul)** | ***trans*ZR** | ***cis*ZR** | **DHZR** | **iPR** | **2MeS*cis*ZR** | **2MeSiPR** |
| **0** | 0.50 ± 0.04 | 9.10 ± 1.38 | 0.97 ± 0.03 | 51.45 ± 0.44 | 0.49 ± 0.00 | 24.92 ± 0.63 | 19.26 ± 1.45 | 12.77 ± 0.55 |
| **1** | 0.91 ± 0.10 | 12.94 ± 0.82 | 0.69 ± 0.02 | 58.32 ± 4.00 | 0.56 ± 0.07 | 24.87 ± 1.03 | 28.94 ± 1.28 | 17.84 ± 1.44 |
| **2** | 0.70 ± 0.17 | 13.78 ± 3.70 | 0.81 ± 0.17 | 61.05 ± 1.85 | 0.59 ± 0.04 | 34.92 ± 4.07 | 21.12 ± 1.35 | 21.45 ± 3.52 |
| **4** | 0.25 ± 0.04 | 5.12 ± 1.13 | 3.00 ± 0.36 | 74.36 ± 5.83 | 0.85 ± 0.10 | 31.20 ± 2.98 | 38.85 ± 3.94 | 21.58 ± 3.02 |
| **7** | 0.24 ± 0.04 | 8.58 ± 1.93 | 2.12 ± 0.26 | 33.96 ± 2.66 | 0.38 ± 0.04 | 26.18 ± 2.50 | 27.13 ± 2.75 | 12.74 ± 1.78 |
| **9** | 0.15 ± 0.06 | 3.89 ± 0.78 | 0.70 ± 0.12 | 15.06 ± 2.24 | 0.45 ± 0.01 | 15.70 ± 2.36 | 13.98 ± 4.60 | 8.40 ± 3.80 |
| **13** | 0.21 ± 0.00 | 7.24 ± 4.50 | 1.07 ± 0.30 | 35.12 ± 5.31 | 0.85 ± 0.41 | 46.29 ± 3.21 | 42.89 ± 0.71 | 23.38 ± 0.06 |
| **14** | 0.23 ± 0.01 | 7.56 ± 0.24 | 0.71 ± 0.01 | 34.40 ± 2.14 | 0.46 ± 0.01 | 44.51 ± 7.54 | 39.30 ± 3.15 | 19.89 ± 1.92 |

**TABLE S5**

| **Time (d)** | **IAA** | **OxIAA** | **IAM** | **PAA** | **IAA-Asp** | **OxIAA-GE** | **IAN** |
| --- | --- | --- | --- | --- | --- | --- | --- |
| **0** | 4.33 ± 0.03 | 8.07 ± 0.48 | 15.27 ± 1.68 | 668.91 ± 106.69 | 0.26 ± 0.10 | 0.04 ± 0.05 | 0.17 ± 0.04 |
| **1** | 7.37 ± 0.15 | 0.63 ± 0.13 | 4.74 ± 0.95 | 495.28 ± 99.06 | 0.30 ± 0.06 | - | 0.11 ± 0.02 |
| **2** | 10.13 ± 1.34 | 2.17 ± 2.54 | 1.72 ± 2.25 | 608.36 ± 77.72 | 0.28 ± 0.33 | 0.11 ± 0.15 | 1.73 ± 0.01 |
| **4** | 21.58 ± 0.98 | 1.77 ± 2.39 | 1.97 ± 0.19 | 757.79 ± 274.09 | 0.08 ± 0.12 | 0.31 ± 0.22 | 0.36 ± 0.30 |
| **7** | 57.13 ± 1.78 | 6.23 ± 0.56 | 3.51 ± 1.10 | 669.93 ± 159.02 | 0.32 ± 0.07 | 0.31 ± 0.01 | 1.37 ± 0.89 |
| **9** | 71.62 ± 3.23 | 9.18 ± 1.31 | 2.31 ± 1.17 | 662.70 ± 41.37 | 0.22 ± 0.08 | 0.10 ± 0.14 | 2.10 ± 1.73 |
| **13** | 84.90 ± 0.96 | 12.18 ± 0.47 | 1.50 ± 0.47 | 724.37 ± 123.24 | 0.05 ± 0.08 | 0.11 ± 0.15 | 0.79 ± 0.16 |
| **14** | 41.42 ± 11.65 | 7.93 ± 2.22 | 0.87 ± 0.97 | 625.74 ± 92.14 | 0.21 ± 0.10 | 0.35 ± 0.24 | 0.41 ± 0.08 |
