# Supplementary material for: Control of cytokinin and auxin homeostasis in cyanobacteria and algae
Source: Ann Bot. 2016 Oct 5;119(1):151–66. doi: 10.1093/aob/mcw194 (PMC5218379; doi:10.1093/aob/mcw194)
Supplement: Supplementary Data [file supp_mcw194_suppl_data.zip › aob-16427-s01.docx]

**SUPPLEMENTARY DATA**

Supplementary data are available in the online version of this article at [www.aob.oxfordjournals.org](http://www.aob.oxfordjournals.org).

**Table S1**:

The list and abbreviations of cyanobacteria and algae species analysed for endogenous cytokinin and auxin profiles in this study.

**Table S2**:

Endogenous cytokinin spectra and concentrations (in pmol g^-1^ FW) in selected cyanobacteria and algae species in the early stationary growth phase. Abbreviations of selected representatives as given in Table S1; abbreviations of cytokinins adopted and modified according to Kamínek *et al*. (2000).

**Table S3**:

Endogenous auxin spectra and concentrations (in pmol g^-1^ FW) in selected cyanobacteria and algae species in the early stationary growth phase. Abbreviations of selected representatives as given in Table S1.

**Table S4**:

Endogenous spectra and concentrations of tRNA-bound cytokinins (related to pmol mg^-1^ tRNA) during *Scenedesmus obliquus* growth cycle. Abbreviations of cytokinins adopted and modified according to Kamínek *et al*. (2000).

**Table S5**:

Endogenous spectra and concentrations of auxins (in pmol g^-1^ FW) during *Scenedesmus obliquus* growth cycle.

**Figure S1**:

Metabolism of exogenously applied [^3^H]*trans*Z in the culture medium of *Klebsormidium flaccidum* in the early stationary growth phase. The peaks represent distribution of radioactivity associated with individual metabolites in the medium 4 and 24 h after [^3^H]*trans*Z application. The products of [^3^H]*trans*Z metabolism were analysed by HPLC coupled to on-line radioactivity detector.

*trans*Z = *trans*-zeatin; DHZ = dihydrozeatin.

**Figure S2**:

Metabolic conversion of [^3^H]*N^6^*-(Δ^2^-isopentenyl)adenine incubated *in vitro* with enzyme cytokinin oxidase/dehydrogenase preparations extracted and partially purified from selected cyanobacteria and algae species in the early stationary growth phase. The *in vitro* assays were performed in 100 mM MOPS-NaOH buffer containing 75 μM 2,6-dichloroindophenol at pH 7.0.

**Figure S3**: Metabolic conversion of [^3^H]*N^6^*-(Δ^2^-isopentenyl)adenine incubated *in vitro* with enzyme cytokinin oxidase/dehydrogenase preparations extracted and partially purified from selected cyanobacteria and algae species in the early stationary growth phase. The *in vitro* assays were performed in 100 mM TAPS-NaOH buffer containing 75 μM 2,6-dichloroindophenol at pH 8.5.
