# Supplementary material for: Control of cytokinin and auxin homeostasis in cyanobacteria and algae
Source: Ann Bot. 2016 Oct 5;119(1):151–66. doi: 10.1093/aob/mcw194 (PMC5218379; doi:10.1093/aob/mcw194)
Supplement: Supplementary Data [file supp_mcw194_suppl_data.zip › aob-16427-s02.docx]

**TABLE S1**

| **Phyllum** | **Order** | **Class** | **CCALA #** | | **Species, authority** | **Abb.** |
| --- | --- | --- | --- | --- | --- | --- |
| Cyanobacteria | Chroococcales | Cyanophyceae | 55 | *Chroococcus minutus* (Kuetzing) Naegeli | | **CM** |
|  | Nostocales | Cyanophyceae | 139 | *Phormidium animale* (C. Agardh ex Gomont) Anagnostidis et Komárek | | **PA** |
|  | Nostocales | Cyanophyceae | 124 | *Nostoc microscopicum* Carmichael | | **NM** |
| Ochrophyta | Tribonematales | Xanthophyceae | 512 | *Tribonema aequale* Pascher | | **TA** |
|  | Mischococcales | Xanthophyceae | 223 | *Bumilleriopsis filiformis* Vischer | | **BF** |
|  | Eustigmatales | Eustigmatophyceae | 514 | *Vischeria helvetica* (Vischer et Pascher) Hibberd | | **VH** |
| Rhodophyta | Porphyridiales | Porphyridiophyceae | 416 | *Porphyridium purpureum* (Bory de Saint-Vincent) K. M. Drew & R. Ross | | **PP** |
|  | Rhodellales | Porphyridiophyceae | 925 | *Rhodella violacea* (Kornmann) Wehrmeyer | | **RV** |
| Chlorophyta | Chlamydomonadales | Chlorophyceae | 283 | *Chlorococcum ellipsoideum* Deason et Bold | | **CE** |
|  | Chlamydomonadales | Chlorophyceae | 282 | *Chlorococcum elbense* Archibald | | **ChE** |
|  | Chlamydomonadales | Chlorophyceae | 421 | *Protosiphon botryoides* (Kuetzing) Klebs | | **PB** |
|  | Chlamydomonadales | Chlorophyceae | 248 | *Chlamydomonas segnis* Ettl | | **CS** |
|  | Chaetophorales | Chlorophyceae | 868 | *Stigeoclonium helveticum* Vischer | | **SH** |
|  | Sphaeropleales | Chlorophyceae | 454 | *Scenedesmus obliquus* (Turpin) Kuetzing | | **SO** |
|  | Ulotrichales | Ulvophyceae | 926 | *Ulothrix crenulata* Kuetzing | | **UC** |
|  | Ulvales | Ulvophyceae | 423 | *Pseudendoclonium basiliense* Vischer | | **PsB** |
|  | Prasiolales | Trebouxiophyceae | 420 | *Prasiolopsis ramosa* Vischer | | **PR** |
|  | Microthamniales | Trebouxiophyceae | 368 | *Microthamnion kuetzingianum* Naegeli | | **MK** |
| Streptophyta | Desmidiales | Zygnematophyceae | 836 | *Actinotaenium curtum* (Ralfs) Teiling ex Ruzicka et Pouzar | | **AC** |
|  | Klebsormidiales | Klebsormidiophyceae | 786 | *Klebsormidium flaccidum* (Kützing) P.C. Silva, K.R. Mattox et W.H. Blackwell | | **KF** |

**TABLE S2**

| **Phyllum** | **Species** | ***trans*Z** | ***trans*ZR** | ***trans*Z7G** | ***trans*Z9G** | ***trans*ZOG** | ***trans*ZROG** | ***trans*ZRMP** | **Σ*trans*Z-types** |
| --- | --- | --- | --- | --- | --- | --- | --- | --- | --- |
| Cyanobacteria | *Chroococcus minutus* (CM) | 0.51 ± 0.18 | - | - | - | - | - | 1.36 ± 0.28 | **1.87** |
|  | *Phormidium animale* (PA) | 0.43 ± 0.10 | - | - | - | - | - | 1.69 ± 0.68 | **2.12** |
|  | *Nostoc microscopicum* (NM) | 4.95 ± 3.06 | - | - | - | - | - | - | **4.95** |
| Ochrophyta | *Tribonema aequale* (TA) | 0.18 ± 0.05 | - | - | - | 0.02 ± 0.01 | - | 0.49 ± 0.36 | **2.81** |
|  | *Bumilleriopsis filiformis* (BF) | 0.79 ± 0.27 | 0.37 ± 0.30 | - | 0.38 ± 0.29 | - | 0.09 ± 0.02 | 1.33 ± 0.43 | **2.96** |
|  | *Vischeria helvetica* (VH) | 2.28 ± 0.73 | - | - | - | - | - | 30.34 ± 1.30 | **32.62** |
| Rhodophyta | *Porphyridium purpureum* (PP) | 0.55 ± 0.40 | 0.02 ± 0.00 | - | - | - | - | 0.98 ± 0.11 | **1.55** |
|  | *Rhodella violacea* (RV) | 23.93 ± 9.57 | - | - | - | - | - | 1.44 ± 0.78 | **25.37** |
| Chlorophyta | *Chlorococcum ellipsoideum* (CE) | 0.27 ± 0.07 | - | - | - | 0.06 ± 0.03 | 0.03 ± 0.00 | 1.17 ± 0.00 | **1.53** |
|  | *Protosiphon botryoides* (PB) | 0.30 ± 0.03 | - | - | - | - | - | - | **0.30** |
|  | *Chlamydomonas segnis* (CS) | 1.38 ± 0.23 | - | - | - | - | - | 0.46 ± 0.10 | **1.84** |
|  | *Stigeoclonium helveticum* (SH) | - | - | - | - | - | - | 6.89 ± 0.24 | **6.89** |
|  | *Scenedesmus obliquus* (SO) | 0.29 ± 0.08 | - | 0.02 ± 0.01 | 0.18 ± 0.03 | - | - | - | **0.49** |
|  | *Ulothrix crenulata* (UC) | 0.13 ± 0.02 | - | - | - | - | - | 1.65 ± 0.22 | **1.78** |
|  | *Pseudendoclonium basiliense* (PsB) | 1.02 ± 0.34 | - | - | - | - | - | 0.49 ± 0.08 | **1.51** |
|  | *Prasiolopsis ramosa* (PR) | 0.63 ± 0.41 | - | - | 0.25 ± 0.13 | - | 0.44 ± 0.38 | 1.33 ± 0.61 | **2.65** |
|  | *Microthamnion kuetzingianum* (MK) | 1.35 ± 0.03 | - | - | - | 0.02 ± 0.01 | - | - | **1.37** |
| Streptophyta | *Actinotaenium curtum* (AC) | - | - | - | - | - | - | 1.29 ± 0.01 | **1.29** |
|  | *Klebsormidium flaccidum* (KF) | 0.22 ± 0.06 | - | - | - | - | - | 13.08 ± 0.45 | **13.30** |

| **Species** | **DHZ** | **DHZR** | **DHZ9G** | **DHZOG** | **DHZRMP** | **ΣDHZ-types** | ***cis*Z** | ***cis*ZR** | **Σ*cis*Z-types** |
| --- | --- | --- | --- | --- | --- | --- | --- | --- | --- |
| **CM** | 0.36 ± 0.15 | 0.54 ± 0.15 | - | - | 0.24 ± 0.11 | **1.14** | 2.42 ± 0.37 | - | **2.42** |
| **PA** | 0.55 ± 0.26 | 0.63 ± 0.50 | - | - | - | **1.18** | 0.39 ± 0.06 | - | **0.39** |
| **NM** | 0.50 ± 0.36 | - | - | - | - | **0.50** | 0.98 ± 0.16 | 0.49 ± 0.35 | **1.47** |
| **TA** | 0.32 ± 0.07 | 0.52 ± 0.01 | 0.07 ± 0.02 | - | 1.07 ± 0.45 | **1.98** | - | - | **-** |
| **BF** | 0.41 ± 0.16 | - | - | - | 1.00 ± 0.05 | **1.41** | 7.77 ± 0.54 | 0.66 ± 0.11 | **8.43** |
| **VH** | 0.35 ± 0.03 | 0.31 ± 0.10 | - | - | 2.78 ± 0.10 | **3.44** | 3.69 ± 0.38 | 7.77 ± 1.06 | **11.46** |
| **PP** | 0.34 ± 0.10 | - | 0.04 ± 0.03 | - | 0.15 ± 0.05 | **0.53** | - | - | **-** |
| **RV** | 0.35 ± 0.09 | - | - | - | - | **0.35** | 0.32 ± 0.12 | 0.77 ± 0.17 | **1.09** |
| **VCE** | 0.19 ± 0.06 | - | - | - | 0.61 ± 0.16 | **0.80** | 0.46 ± 0.05 | 0.32 ± 0.02 | **0.78** |
| **PB** | 0.24 ± 0.11 | 0.14 ± 0.07 | - | - | 1.13 ± 0.19 | **1.51** | 0.74 ± 0.18 | 0.19 ± 0.06 | **0.93** |
| **CS** | 0.37 ± 0.01 | 0.16 ± 0.04 | 0.20 ± 0.03 | 0.06 ± 0.01 | - | **0.79** | 55.91 ± 5.61 | - | **55.91** |
| **SH** | 0.24 ± 0.02 | 0.26 ± 0.19 | - | - | 3.45 ± 0.75 | **3.95** | 0.99 ± 0.05 | 1.74 ± 0.31 | **2.73** |
| **SO** | 0.31 ± 0.00 | 0.34 ± 0.01 | - | - | 0.71 ± 0.21 | **1.36** | 3.65 ± 0.87 | 2.30 ± 1.00 | **5.95** |
| **UC** | 0.37 ± 0.11 | 0.37 ± 0.10 | 0.10 ± 0.06 | - | 1.20 ± 0.06 | **2.04** | - | 0.37 ± 0.03 | **0.37** |
| **PsB** | 0.24 ± 0.03 | 0.45 ± 0.06 | - | - | 0.41 ± 0.02 | **1.10** | 10.93 ± 1.24 | - | **10.93** |
| **PR** | 0.31 ± 0.04 | 0.21 ± 0.15 | - | - | 3.00 ± 0.10 | **3.52** | 0.54 ± 0.08 | - | **0.54** |
| **MK** | 0.46 ± 0.12 | 0.20 ± 0.04 | - | - | - | **0.66** | 9.69 ± 1.22 | - | **9.69** |
| **AC** | - | 0.83 ± 0.19 | - | - | - | **0.83** | 0.68 ± 0.22 | 0.22 ± 0.00 | **0.90** |
| **KF** | 0.53 ± 0.17 | 0.51 ± 0.13 | 0.07 ± 0.05 | - | 2.74 ± 1.12 | **3.85** | 1.13 ± 0.12 | 4.13 ± 0.95 | **5.26** |

| **Species** | **iP** | **iPR** | **iP7G** | **iP9G** | **iPRMP** | **ΣiP-types** | **2MeSiPR** | **Total CKs** |
| --- | --- | --- | --- | --- | --- | --- | --- | --- |
| **CM** | 1.37 ± 0.14 | 0.67 ± 0.16 | - | - | 2.89 ± 0.15 | **4.93** | 3.43 ± 0.16 | **13.79** |
| **PA** | 12.21 ± 0.48 | 7.77 ± 0.19 | - | - | 63.67 ± 7.50 | **83.65** | 91.21 ± 1.94 | **178.55** |
| **NM** | - | - | - | - | 2.19 ± 1.70 | **2.19** | - | **9.11** |
| **TA** | 0.76 ± 0.03 | 0.17 ± 0.03 | - | 0.14 ± 0.10 | 7.37 ± 0.74 | **8.44** | 0.80 ± 0.10 | **14.03** |
| **BF** | 78.35 ± 2.31 | 11.71 ± 0.94 | - | 0.99 ± 0.05 | 7.73 ± 0.30 | **98.78** | 7.44 ± 0.18 | **119.02** |
| **VH** | 0.61 ± 0.05 | 2.99 ± 0.46 | - | - | 6.77 ± 0.61 | **10.37** | 9.14 ± 0.14 | **67.03** |
| **PP** | 0.10 ± 0.03 | - | - | - | 0.35 ± 0.02 | **0.45** | - | **2.53** |
| **RV** | 0.11 ± 0.09 | - | - | - | 0.54 ± 0.15 | **0.65** | 2.11 ± 0.98 | **29.57** |
| **VCE** | 0.28 ± 0.01 | - | - | - | 0.84 ± 0.21 | **1.12** | 1.41 ± 0.05 | **5.64** |
| **PB** | 20.50 ± 1.19 | 0.40 ± 0.19 | - | - | 0.25 ± 0.05 | **21.15** | 3.34 ± 1.54 | **27.23** |
| **CS** | 42.37 ± 1.18 | - | 0.01 ± 0.00 | - | 0.10 ± 0.02 | **42.48** | 0.98 ± 0.28 | **102** |
| **SH** | 0.36 ± 0.00 | - | 1.96 ± 0.24 | - | 2.79 ± 0.04 | **5.11** | 1.41 ± 0.01 | **20.09** |
| **SO** | 1.31 ± 0.29 | 1.44 ± 0.08 | - | 0.08 ± 0.02 | - | **2.83** | 7.04 ± 0.80 | **17.67** |
| **UC** | 0.04 ± 0.01 | 0.20 ± 0.05 | - | - | 0.27 ± 0.10 | **0.51** | 0.28 ± 0.21 | **4.98** |
| **PsB** | 12.26 ± 1.41 | 0.95 ± 0.17 | - | - | 0.32 ± 0.15 | **13.53** | 42.76 ± 8.47 | **69.83** |
| **PR** | 1.17 ± 0.17 | 0.86 ± 0.02 | - | - | 0.42 ± 0.05 | **2.45** | 0.70 ± 0.26 | **9.86** |
| **MK** | 3.25 ± 0.11 | - | - | - | 0.82 ± 0.04 | **4.07** | 8.10 ± 1.80 | **23.89** |
| **AC** | 1.08 ± 0.03 | - | - | - | 0.24 ± 0.07 | **1.32** | - | **4.34** |
| **KF** | 2.25 ± 0.24 | 0.28 ± 0.21 | - | 0.09 ± 0.05 | 1.70 ± 0.03 | **4.32** | 0.29 ± 0.00 | **27.02** |

**TABLE S3**

| **Phyllum** | **Species** | **IAA** | **IAA-Asp** | **OxIAA** | **Σauxins** |
| --- | --- | --- | --- | --- | --- |
| Cyanobacteria | *Chroococcus minutus* (CM) | 192.95 ± 5.90 | 0.03 ± 0.02 | 5.47 ± 0.12 | **198.45** |
|  | *Phormidium animale* (PA) | 250.17 ± 12.32 | 0.09 ± 0.05 | 9.51 ± 0.09 | **259.77** |
|  | *Nostoc microscopicum* (NM) | 9.40 ± 2.82 | 0.05 ± 0.03 | 5.24 ± 2.00 | **14.69** |
| Ochrophyta | *Tribonema aequale* (TA) | 86.36 ± 7.68 | - | 3.37 ± 0.72 | **89.73** |
|  | *Bumilleriopsis filiformis* (BF) | 11.00 ± 0.51 | - | 1.78 ± 0.09 | **12.78** |
|  | *Vischeria helvetica* (VH) | 29.82 ± 1.32 | 0.04 ± 0.01 | 3.16 ± 0.21 | **33.02** |
| Rhodophyta | *Porphyridium purpureum* (PP) | 3.26 ± 1.22 | 0.05 ± 0.03 | 13.19 ± 0.77 | **16.5** |
|  | *Rhodella violacea* (RV) | 11.18 ± 1.96 | - | 5.90 ± 0.59 | **17.8** |
| Chlorophyta | *Chlorococcum ellipsoideum* (CE) | 56.19 ± 3.91 | 0.22 ± 0.09 | 3.78 ± 0.26 | **60.19** |
|  | *Protosiphon botryoides* (PB) | 23.47 ± 2.30 | 0.10 ± 0.02 | 43.54 ± 4.77 | **67.11** |
|  | *Chlamydomonas segnis* (CS) | 13.39 ± 1.52 | - | 10.83 ± 1.65 | **91.34** |
|  | *Stigeoclonium helveticum* (SH) | 287.57 ± 19.89 | 0.03 ± 0.01 | 3.09 ± 0.45 | **290.69** |
|  | *Scenedesmus obliquus* (SO) | 19.59 ± 1.73 | 0.70 ± 0.17 | 27.32 ± 9.75 | **47.61** |
|  | *Ulothrix crenulata* (UC) | 27.17 ± 0.51 | - | 3.18 ± 0.30 | **30.35** |
|  | *Pseudendoclonium basiliense* (PsB) | 6.43 ± 0.52 | - | 4.50 ± 1.12 | **10.93** |
|  | *Prasiolopsis ramosa* (PR) | 148.72 ± 9.48 | 0.15 ± 0.04 | 4.53 ± 0.06 | **153.4** |
|  | *Microthamnion kuetzingianum* (MK) | 45.70 ± 1.29 | 0.21 ± 0.02 | 19.16 ± 3.74 | **65.07** |
| Streptophyta | *Actinotaenium curtum* (AC) | 113.34 ± 4.56 | 0.06 ± 0.03 | 2.76 ± 0.15 | **116.16** |
|  | *Klebsormidium flaccidum* (KF) | 13.61 ± 0.35 | - | 7.61 ± 0.66 | **21.22** |

**TABLE S4**

|  |  |  | pmol mg^-1^ tRNA | | | | pmol mg^-1^ tRNA | |
| --- | --- | --- | --- | --- | --- | --- | --- | --- |
| **Time (d)** | **ng tRNA/g DW** | **tRNA (ng/ul)** | ***trans*ZR** | ***cis*ZR** | **DHZR** | **iPR** | **2MeS*cis*ZR** | **2MeSiPR** |
| **0** | 0.50 ± 0.04 | 9.10 ± 1.38 | 0.97 ± 0.03 | 51.45 ± 0.44 | 0.49 ± 0.00 | 24.92 ± 0.63 | 19.26 ± 1.45 | 12.77 ± 0.55 |
| **1** | 0.91 ± 0.10 | 12.94 ± 0.82 | 0.69 ± 0.02 | 58.32 ± 4.00 | 0.56 ± 0.07 | 24.87 ± 1.03 | 28.94 ± 1.28 | 17.84 ± 1.44 |
| **2** | 0.70 ± 0.17 | 13.78 ± 3.70 | 0.81 ± 0.17 | 61.05 ± 1.85 | 0.59 ± 0.04 | 34.92 ± 4.07 | 21.12 ± 1.35 | 21.45 ± 3.52 |
| **4** | 0.25 ± 0.04 | 5.12 ± 1.13 | 3.00 ± 0.36 | 74.36 ± 5.83 | 0.85 ± 0.10 | 31.20 ± 2.98 | 38.85 ± 3.94 | 21.58 ± 3.02 |
| **7** | 0.24 ± 0.04 | 8.58 ± 1.93 | 2.12 ± 0.26 | 33.96 ± 2.66 | 0.38 ± 0.04 | 26.18 ± 2.50 | 27.13 ± 2.75 | 12.74 ± 1.78 |
| **9** | 0.15 ± 0.06 | 3.89 ± 0.78 | 0.70 ± 0.12 | 15.06 ± 2.24 | 0.45 ± 0.01 | 15.70 ± 2.36 | 13.98 ± 4.60 | 8.40 ± 3.80 |
| **13** | 0.21 ± 0.00 | 7.24 ± 4.50 | 1.07 ± 0.30 | 35.12 ± 5.31 | 0.85 ± 0.41 | 46.29 ± 3.21 | 42.89 ± 0.71 | 23.38 ± 0.06 |
| **14** | 0.23 ± 0.01 | 7.56 ± 0.24 | 0.71 ± 0.01 | 34.40 ± 2.14 | 0.46 ± 0.01 | 44.51 ± 7.54 | 39.30 ± 3.15 | 19.89 ± 1.92 |

**TABLE S5**

| **Time (d)** | **IAA** | **OxIAA** | **IAM** | **PAA** | **IAA-Asp** | **OxIAA-GE** | **IAN** |
| --- | --- | --- | --- | --- | --- | --- | --- |
| **0** | 4.33 ± 0.03 | 8.07 ± 0.48 | 15.27 ± 1.68 | 668.91 ± 106.69 | 0.26 ± 0.10 | 0.04 ± 0.05 | 0.17 ± 0.04 |
| **1** | 7.37 ± 0.15 | 0.63 ± 0.13 | 4.74 ± 0.95 | 495.28 ± 99.06 | 0.30 ± 0.06 | - | 0.11 ± 0.02 |
| **2** | 10.13 ± 1.34 | 2.17 ± 2.54 | 1.72 ± 2.25 | 608.36 ± 77.72 | 0.28 ± 0.33 | 0.11 ± 0.15 | 1.73 ± 0.01 |
| **4** | 21.58 ± 0.98 | 1.77 ± 2.39 | 1.97 ± 0.19 | 757.79 ± 274.09 | 0.08 ± 0.12 | 0.31 ± 0.22 | 0.36 ± 0.30 |
| **7** | 57.13 ± 1.78 | 6.23 ± 0.56 | 3.51 ± 1.10 | 669.93 ± 159.02 | 0.32 ± 0.07 | 0.31 ± 0.01 | 1.37 ± 0.89 |
| **9** | 71.62 ± 3.23 | 9.18 ± 1.31 | 2.31 ± 1.17 | 662.70 ± 41.37 | 0.22 ± 0.08 | 0.10 ± 0.14 | 2.10 ± 1.73 |
| **13** | 84.90 ± 0.96 | 12.18 ± 0.47 | 1.50 ± 0.47 | 724.37 ± 123.24 | 0.05 ± 0.08 | 0.11 ± 0.15 | 0.79 ± 0.16 |
| **14** | 41.42 ± 11.65 | 7.93 ± 2.22 | 0.87 ± 0.97 | 625.74 ± 92.14 | 0.21 ± 0.10 | 0.35 ± 0.24 | 0.41 ± 0.08 |
