# Supplementary figures and images for: Control of cytokinin and auxin homeostasis in cyanobacteria and algae
Source: Ann Bot. 2016 Oct 5;119(1):151–66. doi: 10.1093/aob/mcw194 (PMC5218379; doi:10.1093/aob/mcw194)

## Slide 1
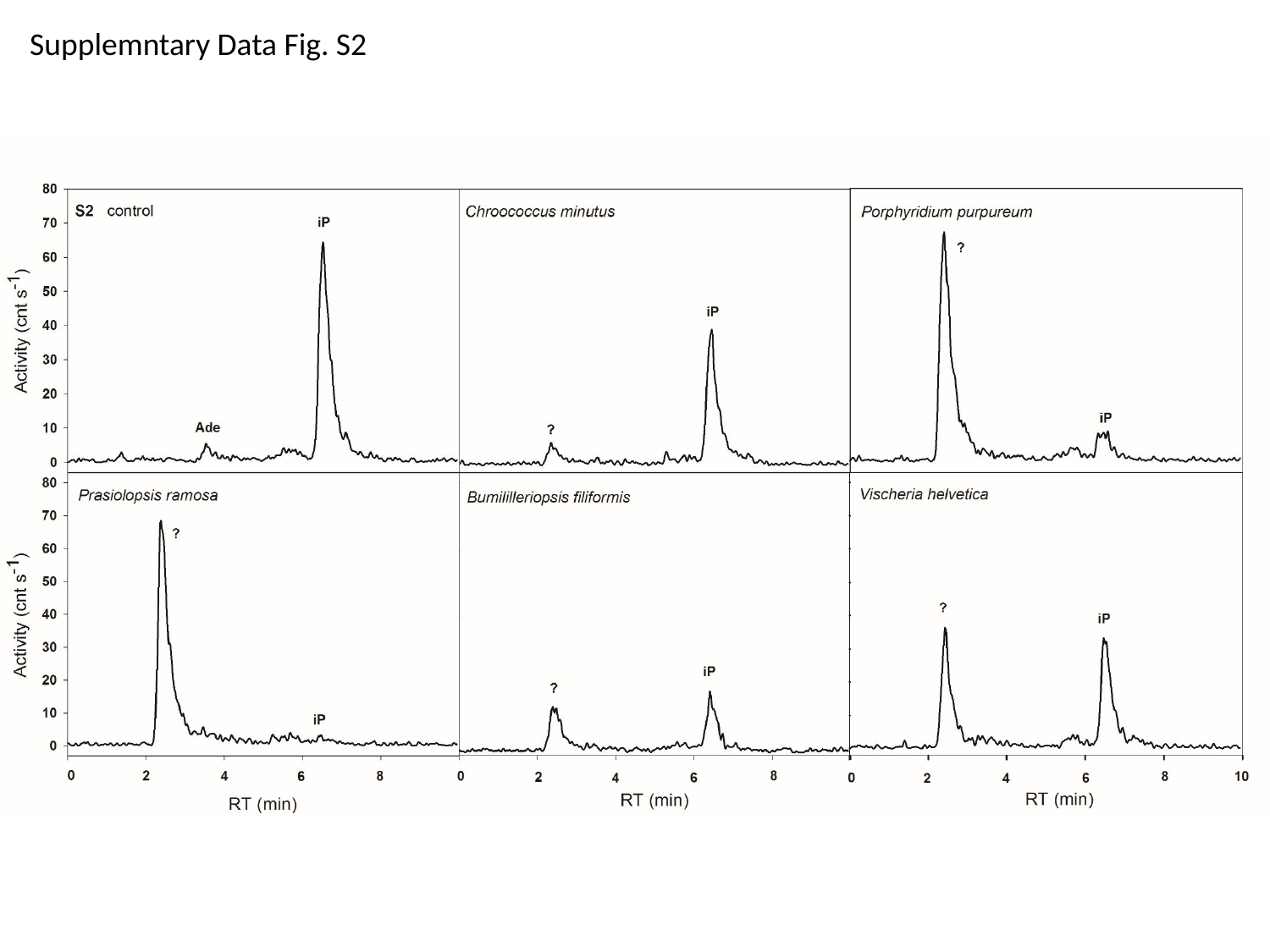

Supplemntary Data Fig. S2

Supplement: Supplementary Data [file supp_mcw194_suppl_data.zip › aob-16427-s04.pptx]

## Slide 1
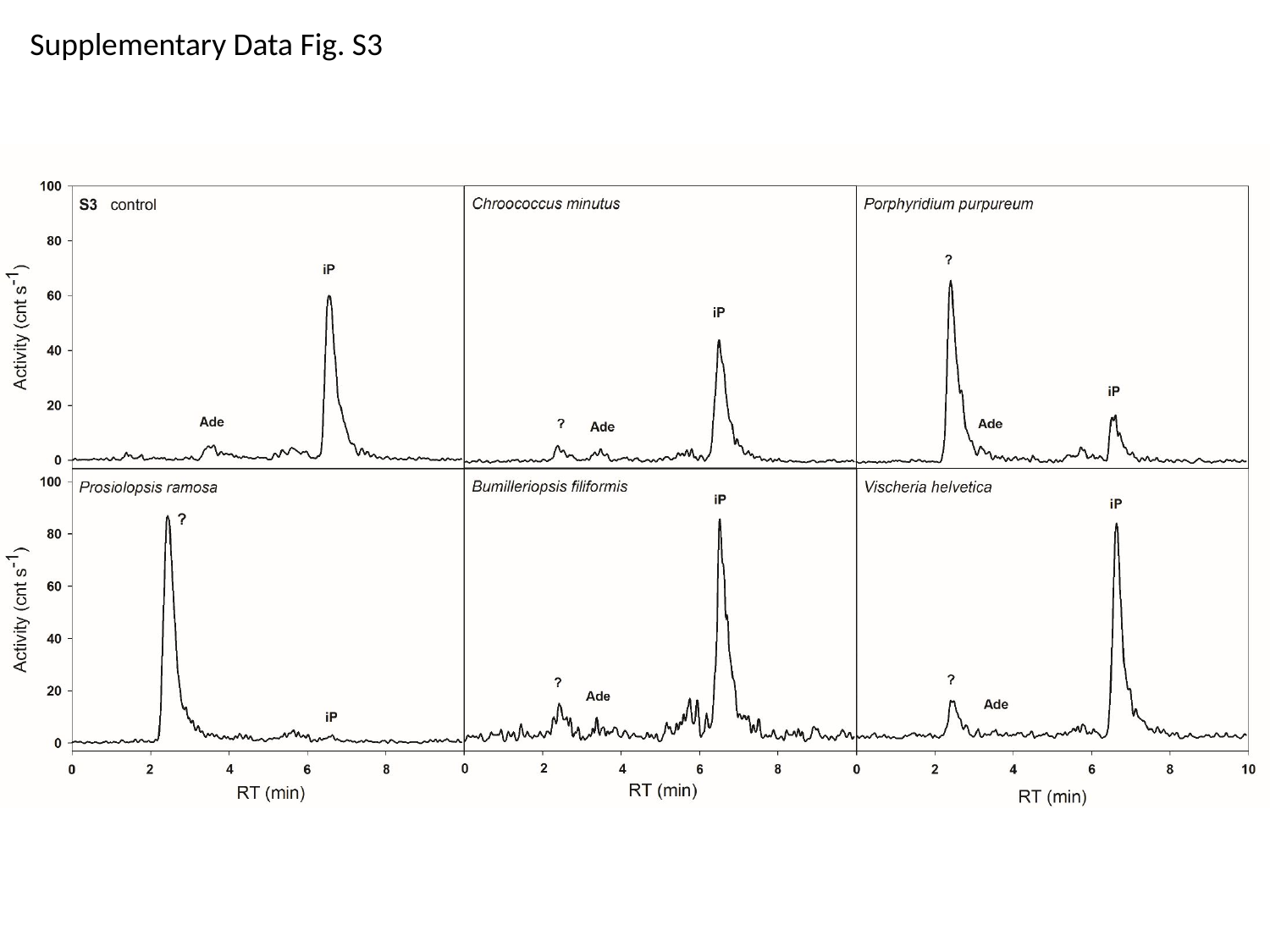

Supplementary Data Fig. S3

Supplement: Supplementary Data [file supp_mcw194_suppl_data.zip › aob-16427-s05.pptx]

## Slide 1
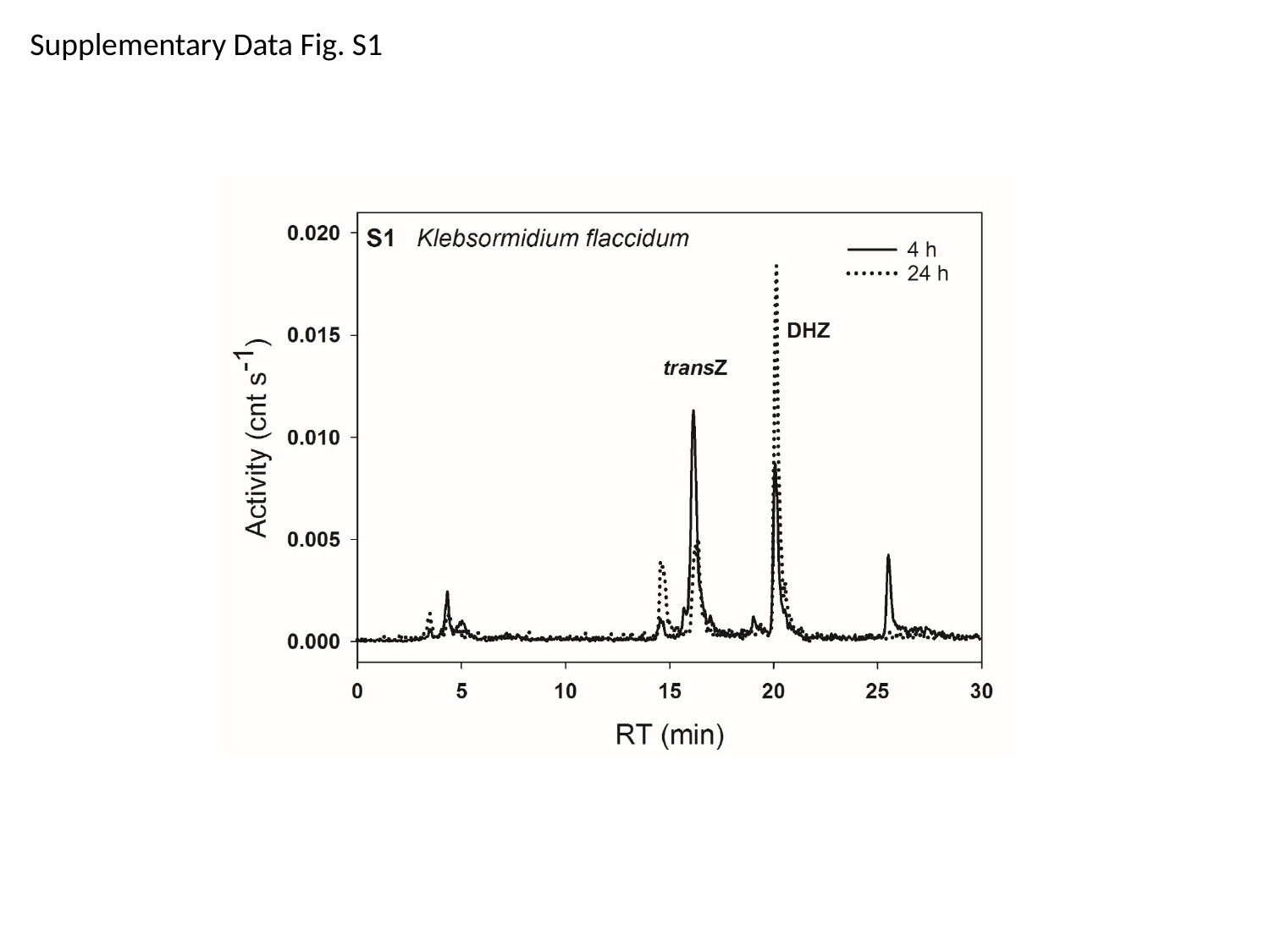

Supplementary Data Fig. S1

Supplement: Supplementary Data [file supp_mcw194_suppl_data.zip › aob-16427-s03.pptx]
